# Mannose-binding lectin 2 secreted by hepatocellular carcinoma cells recruits and activates natural killer cells to reshape an immune-activated microenvironment

**DOI:** 10.1371/journal.pbio.3003793

**Published:** 2026-05-20

**Authors:** Hangyu Liao, Jun Yang, Lei Cai, Luhao Chi, Chunming Wang, Yuyan Xu, Juncheng Xie, Kunling Chen, Jingyuan Pei, Zesheng Jiang, Mingxin Pan, Liang Zhao

**Affiliations:** 1 General Surgery Center, Department of Hepatobiliary Surgery II, Guangdong Provincial Research Center for Artificial Organ and Tissue Engineering, Guangzhou Clinical Research and Transformation Center for Artificial Liver, Institute of Regenerative Medicine, Zhujiang Hospital, Southern Medical University, Guangzhou, China; 2 Department of Pathology, Shunde Hospital of Southern Medical University, Foshan, China; 3 Department of Pathology & Guangdong Province Key Laboratory of Molecular Tumor Pathology, School of Basic Medical Sciences, Southern Medical University, Guangzhou, China; The University of Sheffield, UNITED KINGDOM OF GREAT BRITAIN AND NORTHERN IRELAND

## Abstract

Crosstalk between hepatocellular carcinoma (HCC) and the tumor microenvironment (TME) is pivotal for the initiation and management of HCC. The infiltration and function of natural killer (NK) cells in the TME are frequently hindered. However, it is unclear whether a crucial regulatory factor originating from HCC cells directly modulates NK cell activity to evade immune surveillance. In this study, we found that mannose-binding lectin 2 (MBL2) expression was markedly decreased in HCC and positively correlated with HCC prognosis. MBL2 inhibited the proliferation and migration of HCC cells intracellularly. Human and murine co-culture systems of HCC and NK cells were established to demonstrate that secreted MBL2 recruited and activated NK cells in the TME, particularly upregulating the infiltration of NKp46^+^ NK cells. Furthermore, secreted MBL2 promoted the production of IL-13 and IL-25 by NK cells, resulting in a decrease in exhausted cytotoxic T lymphocytes. Mechanistically, MBL2 interacts with the integrin β1 receptor, activating the FAK/AKT pathway and increasing PD-L1 expression on NK cells. Our discovery identifies MBL2 as an NK cell–activating cytokine, initiating the integrin β1/FAK/AKT pathway in NK cells and reshaping an immune-activated microenvironment of HCC. Strategies to up-regulate MBL2 may enhance the anti-PD-L1 immunotherapy efficacy and serve as a potential therapeutic approach for HCC.

## Abstract 2

Natural killer (NK) cells have a main role in the tumor microenvironment, but the mechanisms underlying their activity in hepatocellular carcinoma remain unclear. This study shows that mannose-binding lectin 2 (MBL2) secreted by hepatocellular carcinoma cells activates NK cells via integrin β1–FAK–AKT signaling and reshapes an immune-activated microenvironment.

## 1. Introduction

Hepatocellular carcinoma (HCC) is one of the most common malignant tumors and the third leading cause of cancer-related death worldwide [1]. Interactions between HCC cells and the immune system play a crucial role in the development and treatment of HCC [2]. Natural killer (NK) cells in the liver play a pivotal role in innate immunity, serving as the first line of defense in host immune surveillance and a major checkpoint in the immune evasion process of HCC cells [3]. NK cells possess intrinsic anti-tumor functions, whereby activated NK cells kill tumor cells by releasing cytotoxic granules and inducing apoptosis, thereby inhibiting tumor growth [4]. Moreover, NK cells produce immunomodulatory molecules, such as interferons (IFN), which activate other immune cells, such as macrophages and T cells, to enhance immune responses [5,6]. The activating receptors on NK cells (NKG2D and NKp46), along with the cytotoxic proteins granzyme B (GZMB) and perforin, play critical roles in the recognition and elimination of tumors by NK cells [7]. In addition to directly killing HCC cells through cell-to-cell contact, activated NK cells produce IFN-γ, which positively influences anti-tumor immunity against HCC [8]. However, owing to tumor antigen alterations and an increase in immunosuppressive cells within the tumor microenvironment (TME), NK cells can fail to properly recognize and eliminate target cells. Myeloid-derived suppressor cells in patients with HCC directly inhibit the cytotoxic effects of NK cells in a contact-dependent manner, whereas regulatory T cells suppress IFN-γ production in tumor-infiltrating NK cells and diminish their cytotoxic capabilities [9]. Therefore, elucidating the alterations in NK cell functional status during tumor initiation and progression is crucial.

Cytokines in the TME play a critical regulatory role in influencing the quantity and activity of NK cells [10,11]. IL-12 and IL-18 are crucial factors for NK cell activation and enhanced cytotoxicity, [12–14], whereas excessive IL-10 production in HCC is linked to impaired NK cell function [15]. However, whether cytokines derived from HCC cells directly regulate NK cell activity to evade immune surveillance remains unclear.

Mannose-binding lectin 2 (MBL2) is primarily synthesized by hepatocytes and then secreted into the extracellular matrix and plasma [16]. MBL2 either activates the complement activation lectin pathway, exerting an indirect immunoregulatory effect, or binds to phagocytic cell lectin receptors, acting as a direct modulator [17]. Thus, deficiency or dysfunction of MBL2 increases the risk of infection and autoimmune diseases [18]. Previous research on liver diseases has shown that low MBL2 expression is associated with chronic hepatitis B virus (HBV) infection [19]. Moreover, polymorphisms in *MBL2* are associated with susceptibility to HCC and can influence its progression and prognosis [20,21]. Although MBL2 is a crucial regulatory factor in HCC, its role in modulating innate immunity within HCC microenvironment, as well as the specific immune cell types involved in tumor immune regulation, has yet to be fully elucidated.

In this study, we aimed to identify cytokines derived from HCC cells that are associated with HBV infection and that possess the potential to reshape an immunotherapy-activated TME. We investigated the intracellular and extracellular regulatory role of MBL2 in HCC proliferation and invasion. Further, we elucidated the molecular mechanisms by which MBL2 recruits and activates NK cells in the HCC microenvironment. Our results demonstrate that MBL2 can up-regulate the expression of PD-L1 in both HCC and NK cells. These findings suggest that strategies to up-regulate MBL2 can enhance the effectiveness of PD-L1 immunotherapy and serve as a therapeutic approach for HCC.

## 2. Results

### 2.1. Endogenous MBL2 inhibits the proliferation and migration of HCC cells

**Fig 1:**
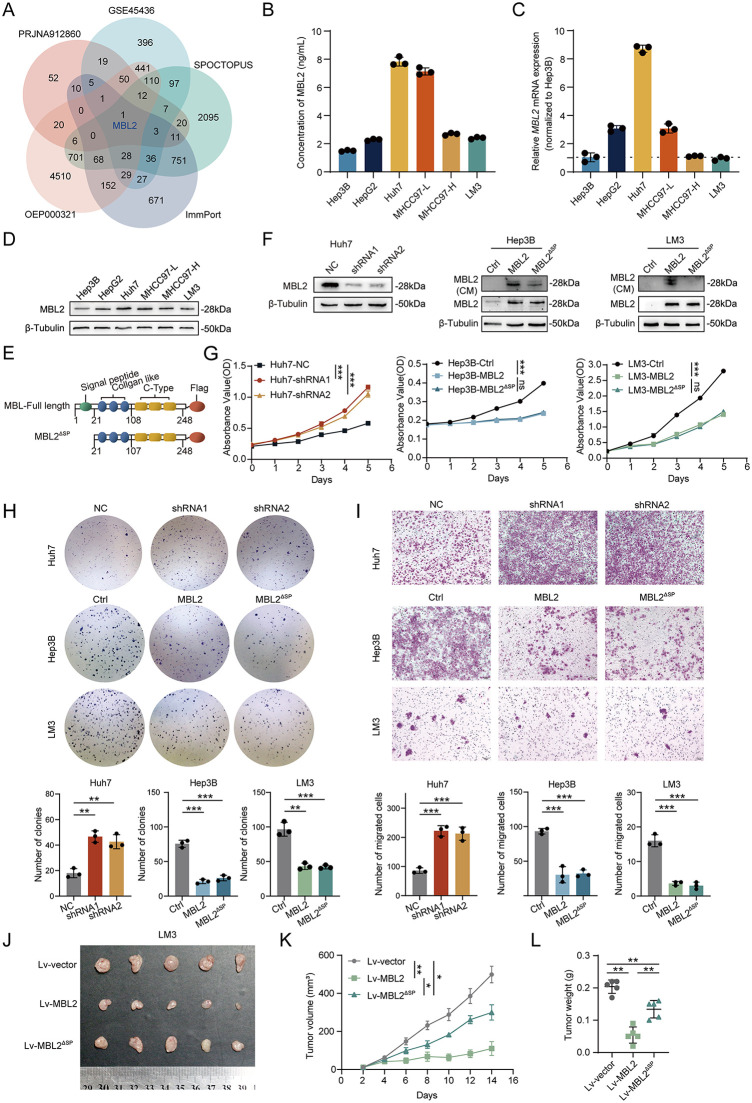
Endogenous MBL2 inhibits HCC proliferation and migration intracellularly. **(A)** Venn diagram of the PRJNA912860 dataset, GSE45436 dataset, SPOCTOPUS, OEP000321 dataset and ImmPort. **(B)** ELISA was used to measure extracellular MBL2 in the culture supernatants of HCC cell lines. (C, D) qPCR and WB were used to detect MBL2 at the transcriptional and translational levels. **(E)** Schematic illustration of MBL2 constructs. **(F)** WB was used to assess the knockdown efficiency of MBL2 in human Huh7 cells and the MBL2 expression levels in human Hep3B and LM3 cells. **(G, H)** Impact of endogenous MBL2 on HCC proliferation was assessed using CCK-8 (G) and colony formation assays **(H)**. **(I)** Transwell assays were employed to assess the impact of endogenous MBL2 on HCC cell migration. **(J)** Nude mouse subcutaneous HCC models of the vector group, MBL2 group, and MBL2^ΔSP^ group. **(K, L)** Growth curve and tumor weight of the subcutaneous tumor model in nude mice. All values are shown as mean ± SD. *p < 0.05, **p < 0.01, ***p < 0.001. ns indicates no significance. Source data are available in S1 Data. Abbreviations: MBL2, Mannose-binding lectin 2; MBL2^ΔSP^, MBL2 protein lacking the signal peptide.

Recent studies have identified CTCs in the bloodstream as a marker of HCC early metastasis. We performed noninvasive quantification of CTCs using liquid biopsy techniques. High-throughput transcriptome sequencing between HCC tissues with CTC-High level and CTC-Low levels (SRA data: PRJNA912860) revealed differential genes promoting metastasis and immune escape in hepatocellular carcinoma. The above sequencing results were combined with The Human Secretome and Membrane Proteome (HPA SPOCTOPUS), HBV-related HCC dataset (OEP000321), The Immunology Database and Analysis Portal (ImmPort) and GSE45436 dataset to identify the differential cytokines derived from HCC cells with potential for TME regulation. The intersection result showed MBL2 has the potential to modulate the HCC immune microenvironment, which is secreted into the extracellular matrix of HCC and correlates with tumor proliferation and metastasis (Fig 1A). MBL2 is predominantly synthesized by hepatocytes and secreted into the circulation in humans [16]. To determine the basal secretion level of MBL2 in human HCC cell lines, culture medium (CM) supernatants of Hep3B, HepG2, Huh7, MHCC97-L (97L), MHCC97-H (97H), and LM3 cells were collected. ELISA assays were utilized to assess the secretion of MBL2 in HCC cell lines. The results indicated that the MBL2 secretion levels of Huh7 and 97L cells were relatively high, whereas the MBL2 secretion of Hep3B, HepG2 and LM3 cells was relatively weak (Fig 1B). We used q-PCR and WB to clarify the expression of MBL2 in HCC cells at the transcriptional and translational levels, respectively. q-PCR highlighted that the *MBL2* mRNA expression was relatively high in Huh7 and relatively low in Hep3B, 97H, and LM3 cells, after normalizing the mRNA of MBL2 in Hep3B (Fig 1C). The Human Protein Atlas database showed that the expression of MBL2 was highest in Huh7 cells, whereas the expression in Hep3B cells was relatively low (S1A Fig), which was consistent with our results. In the WB results, Huh7 and 97L cell lines showed higher MBL2 levels than other cell lines, whereas Hep3B and LM3 cells showed lower MBL2 expression (Fig 1D). Therefore, Huh7 cells were employed for *MBL2* knockdown, and Hep3B and LM3 cells were adopted to construct cell lines stably overexpressing the *MBL2* gene. Secretion of MBL2 to the extracellular compartment is blocked by the absence of signal peptides in HCC. To discern both the intracellular and extracellular impacts of endogenous MBL2 on HCC progression, full-length MBL2 molecules and truncated MBL2 proteins lacking the signal peptide (MBL2^ΔSP^) were generated following the human genome sequence (Fig 1E). After employing lentiviral transfection, qPCR (S1B Fig) and WB (Fig 1F) confirmed the stable construction of MBL2-knockdown cell lines (Huh7-MBL2-shRNA1&2) and the establishment of full-length MBL2 and MBL2^ΔSP^ overexpression cell lines (Hep3B-MBL2, Hep3B-MBL2^ΔSP^, LM3-MBL2, and LM3-MBL2^ΔSP^) at the transcriptional and translational levels, respectively.

To explore the regulation of endogenous MBL2 on the proliferation of HCC cells, we used CCK-8 and colony formation assays to detect the proliferative ability of HCC after MBL2 interference and MBL2 overexpression. The CCK-8 assays showed that the OD values of the Huh7-MBL2-shRNA1 group and Huh7-MBL2-shRNA2 group increased significantly relative to the Huh7-NC group. In contrast, the OD values of the Hep3B-MBL2 and LM3-MBL2 groups were significantly decreased relative to the Hep3B-Ctrl and LM3-Ctrl groups (Fig 1G). To investigate whether endogenous MBL2 inhibits HCC proliferation intracellularly or extracellularly, the differences in OD values between MBL2 and MBL2^ΔSP^ groups in HCC cells were addressed. CCK-8 assays demonstrated no differences between the MBL2 group and the MBL2^ΔSP^ group in Hep3B and LM3 cells. Similarly, there was no significant difference between the LM3-MBL2 group and LM3-MBL2^ΔSP^ group (Fig 1G). Moreover, stimulation of Hep3B and LM3 cells with different concentrations of recombinant MBL2 (rMBL2) did not suppress HCC cell proliferation (S1C Fig). Colony formation assays exhibited an increase in clonal clusters in the Huh7-MBL2-shRNA1 and Huh7-MBL2-shRNA2 groups compared to the Huh7-NC group. The number of clones was reduced in both the MBL2 group and the MBL2^ΔSP^ group compared to the Ctrl group in both Hep3B and LM3 cells, whereas no difference was observed in the number of clones between the MBL2 group and MBL2^ΔSP^ group (Fig 1H). Likewise, Transwell assays showed a significant increase in the number of migrated cells in the Huh7-MBL2-shRNA1 and Huh7-MBL2-shRNA2 groups relative to the Huh7-NC group. The number of migrated cells in Hep3B and LM3 cells was significantly decreased in the MBL2 and MBL2^ΔSP^ groups relative to the Ctrl group, without a significant difference between the MBL2 group and MBL2^ΔSP^ group (Fig 1I). These findings elucidated that MBL2 inhibits HCC proliferation intracellularly and does not regulate HCC cells after secretion.

To verify the regulatory role of secreted MBL2 in HCC progression, we established a subcutaneous tumor model by injecting LM3-MBL2, LM3-MBL2^ΔSP^, and LM3-vector cells into five-week-old male nude mice, given the high structural conservation of MBL2 between humans and mice (Fig 1J). Analysis of the subcutaneous tumor growth curve showed that the tumor volume in the LM3-MBL2 group increased more slowly than that in the LM3-vector group. Notably, the tumor volume in the LM3-MBL2^ΔSP^ group grew more rapidly than that in the LM3-MBL2 group (Fig 1K). At the tumor harvesting time point in the third week, a comparison of tumor masses revealed that the LM3-MBL2 group had a significantly smaller tumor mass than the control group, whereas the tumor mass in the LM3-MBL2^ΔSP^ group rebounded relative to that in the LM3-MBL2 group (Fig 1L). These findings suggest that blocking MBL2 secretion weakens its inhibitory effect on HCC progression, indicating that secreted MBL2 may regulate the TME and indirectly inhibit HCC progression.

### 2.2. Secreted MBL2 recruits and activates NK cells in the TME

**Fig 2:**
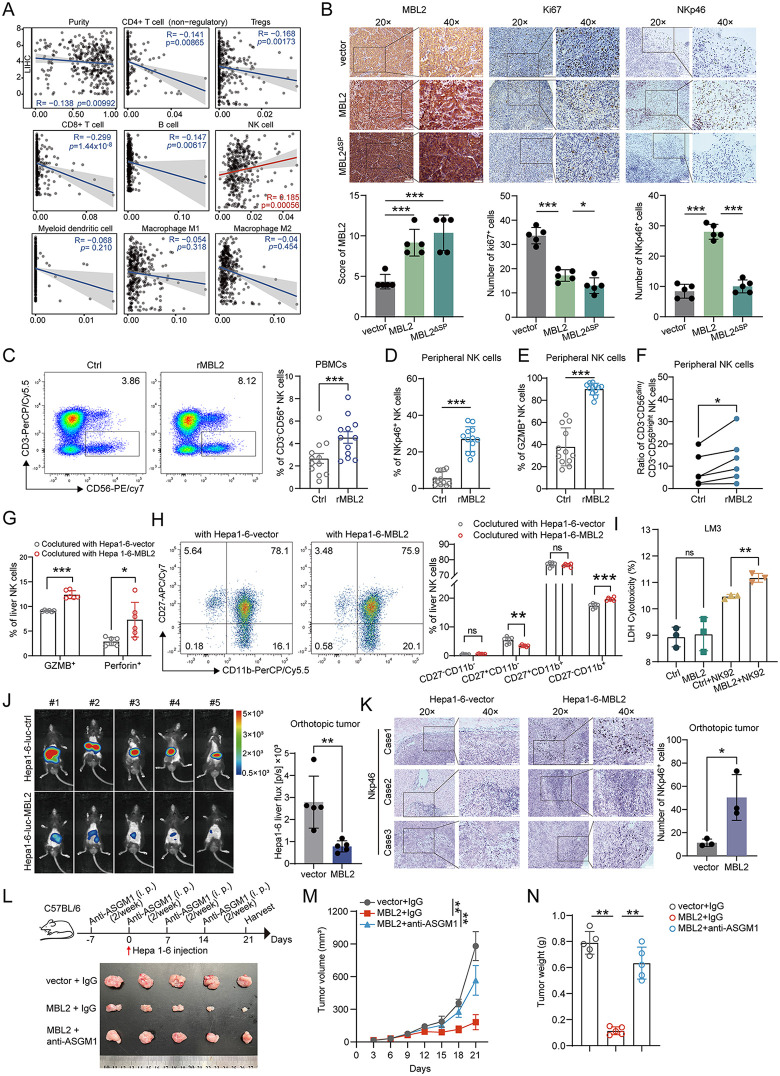
Secreted MBL2 recruits and activates NK cells in HCC. **(A)** Correlation between MBL2 and infiltrating lymphocytes in HCC was analyzed using the TIMER 2.0 database. **(B)** IHC was employed to assess MBL2, Ki67, and NK cell infiltration in the subcutaneous HCC model. The statistical charts are shown on the bottom. Scale bars represent 100 μm at 20× magnification and 50 μm at 40× magnification. **(C)** Flow cytometry was used to assess the proportion of CD3^–^CD56^+^ NK cells in PBMCs. **(D, E)** The expression of NKp46 (D) and GZMB (E) on peripheral NK cells was determined by flow cytometry after MBL2 stimulation. **(F)** The ratio of CD3^–^CD56^dim^ and CD3^–^CD56^bright^ NK cell subsets in peripheral NK cells. **(G, H)** Flow cytometry was used to assess GZMB and perforin expression (G) in mouse hepatic NK cells after co-culture with Hepa1-6-MBL2 cells; and the proportions of CD27^–^CD11b^–^, CD27^+^CD11b^–^, CD27^+^CD11b^+^, and CD27^–^CD11b^+^ subsets (H) in hepatic NK cells. **(I)** The LDH assay was employed to evaluate the cytotoxicity of NK92 cells after co-culture with LM3-MBL2 cells. **(J)** In vivo live animal imaging was used to compare the size of orthotopic HCC between Hepa1-6-luc-MBL2 group and Hepa1-6-luc-vector group. **(K)** IHC was employed to assess the infiltration of NKp46^+^ NK cells in the orthotopic HCC model. Scale bars represent 100 μm at 20× magnification and 50 μm at 40× magnification. **(L)** Subcutaneous HCC models were established in immunocompetent mice for the following groups: vector + IgG, MBL2 + IgG, and MBL2 + anti-ASGM1. A schematic representation of the experimental procedure is shown at the top. **(M, N)** Tumor growth curve and final tumor weight in the subcutaneous HCC model. All values are shown as mean ± SD. *p < 0.05, **p < 0.01, ***p < 0.001. ns indicates no significance. Source data are available in S1 Data. Abbreviations: PBMCs, peripheral blood mononuclear cells; rMBL2, recombinant MBL2; LDH, lactate dehydrogenase.

The correlation between MBL2 expression and the infiltration of lymphocytes within HCC was investigated using the TIMER analysis platform [22]. TIMER analysis revealed that the expression of MBL2 decreased with elevated purity of HCC cells in the microenvironment. There was no significant correlation between the expression of MBL2 and the level of infiltration of dendritic cells and macrophages, and a negative correlation was observed between MBL2 and CD4^+^ T cells, CD8^+^ T cells, Tregs, and B cells. Notably, there was a statistically significant positive correlation (R = 0.185) between MBL2 and NK cell infiltration levels (Fig 2A). TIMER analysis suggested that secreted MBL2 may regulate infiltrating NK cells and CD8^+^ T cells in HCC. Flow cytometry was employed to assess the proportions of NK cells and CD8^+^ T cells within the peripheral blood mononuclear cells (PBMCs) cultured with rMBL2. The results demonstrated that rMBL2 exerted a direct upregulatory effect on the proportion of NK cells (Fig 2C), whereas CD8^+^ T cells were not directly regulated (S2A Fig). To assess the impact of secreted MBL2 on NK cell recruitment in HCC, we used immunohistochemistry (IHC) to reveal the infiltration of NKp46^+^ NK cells in the subcutaneous tumors of BALB/c nude mice. The results showed a lower number of Ki67^+^ HCC cells in the LM3-MBL2 group compared with the LM3-vector group, and similarly showed a lower Ki67^+^ cell count in the LM3-MBL2^ΔSP^ group. Intriguingly, NKp46^+^ NK cell infiltration was significantly increased in the LM3-MBL2 group compared to the control group, whereas the infiltration of NKp46^+^ NK cells was reduced in the LM3-MBL2^ΔSP^ group relative to the LM3-MBL2 group (Fig 2B). This finding suggested that MBL2 downregulated the number of Ki67^+^ HCC cells in the subcutaneous tumors, indicating that high MBL2 expression inhibits HCC proliferation. Furthermore, the inhibition of MBL2 secretion from HCC cells was associated with a reduced abundance of infiltrating NK cells.

To investigate the activation of NK cells by MBL2, we collected PBMCs from 12 healthy donors to obtain peripheral NK cells (S2B Fig), which were then cultured with rMBL2 or an equal volume of PBS. The proportion of NKG2D^+^ NK cells and NKp46^+^ NK cells among peripheral CD3^–^CD56^+^ NK cells was detected by flow cytometry after 72-hour cultivation. The MBL2 group showed significantly elevated NKG2D (S2C Fig) and NKp46 receptor (Figs 2D and S2D) expression in peripheral CD3^–^CD56^+^ NK cells, suggesting that MBL2 boosted the activation of NK cells. We further assessed CD107a surface expression and GZMB production by NK cells. CD107a expression (S3A–S3D Fig) and GZMB production (Figs 2E and S3E) were increased in the rMBL2 group relative to the PBS group. These results suggested that MBL2 directly boosted the activation and cytotoxicity of NK cells. CD3^–^CD56^bright^ NK cells and CD3^–^CD56^dim^ NK cells are the two main subpopulations of functional NK cells. An increased proportion of CD3^–^CD56^dim^ NK cells within the CD3^–^CD56^+^ NK cell population, along with an elevated CD3^–^CD56^dim^/CD3^–^CD56^bright^ ratio, was observed following co-culture with rMBL2 (Figs 2F and S3F). Flow cytometry indicated amplified NKG2D expression in both CD3^–^CD56^dim^ and CD3^–^CD56^bright^ NK cell subpopulations in the MBL2 group (S3G, S3H Fig).

To comprehensively address the modulation of infiltrating NK cells by secreted MBL2 in TME, mouse hepatic NK cells were extracted (S3I Fig) and then co-cultured with Hepa1–6 cells, a mouse HCC cell line, in a mouse co-culture system (S3J Fig). Compared to the Hepa1–6-vector group, the expression of both GZMB and perforin was upregulated in mouse liver NK cells after co-culture with Hepa1–6-MBL2 cells (Fig 2G). The proportion of CD27^+^CD11b^–^ NK cells was also decreased after co-culture with Hepa1–6-MBL2 compared to the Hepa1–6-vector group, whereas the proportion of CD27^–^CD11b^+^ NK cells increased markedly (Fig 2H). Subsequently, we established an in vitro human HCC–NK cell co-culture model using LM3 and Hep3B cells together with human NK92 cells (S3K Fig). Transwell assays, followed by lactate dehydrogenase (LDH) cytotoxicity assays, revealed that MBL2 significantly promoted NK92 cell recruitment (S3L Fig). LDH assays demonstrated that MBL2 did not significantly induce HCC apoptosis in the absence of NK cells. However, upon co-culture with NK cells, MBL2 treatment significantly enhanced HCC apoptosis compared to the control group (Figs 2I and S3M). These results showed that MBL2 directly enhanced the cytotoxicity of HCC-infiltrating NK cells and had the potential to promote the recruitment and maturation of NK cells.

To verify the MBL2-mediated recruitment and activation of HCC-infiltrating NK cells in vivo, Hepa1–6-luc-MBL2 cells and Hepa1–6-luc-vector cells were inoculated into the left lateral lobe of the liver in male C57BL/6 mice to establish a murine orthotopic HCC model. Two weeks after inoculation, in vivo bioluminescence imaging was performed to assess tumor burden in mice (Fig 2J). A significant reduction in tumor burden was observed in the Hepa1–6-luc-MBL2 group compared to the Hepa1–6-luc-vector group. Notably, IHC showed increased infiltration of NKp46^+^ NK cells at the tumor margins in the Hepa1–6-luc-MBL2 group compared to that in the Hepa1–6-luc-vector group (Fig 2K). To assess the role of NK cell activation in the MBL2-mediated suppression of HCC growth, a subcutaneous tumor model was established in immunocompetent C57BL/6 mice (Fig 2L). NK cells were depleted using the anti-asialo GM1 (anti-ASGM1) antibody. Tumors in the MBL2 group exhibited slower growth compared to the control group. However, tumor growth in the MBL2 + anti-ASGM1 group was partially restored relative to the MBL2 group alone (Fig 2M). Consistently, tumor weight in the MBL2 group was reduced compared to the control group, while the MBL2 + anti-ASGM1 group showed a partial reversal of this reduction (Fig 2N). These findings showed that NK cell depletion attenuated the inhibitory effect of MBL2 on HCC progression in vivo, indicating that MBL2 may suppress HCC growth by promoting NK cell activity.

### 2.3. MBL2 activates the PI3K/AKT pathway in NK cells through the integrin β1 receptor

**Fig 3:**
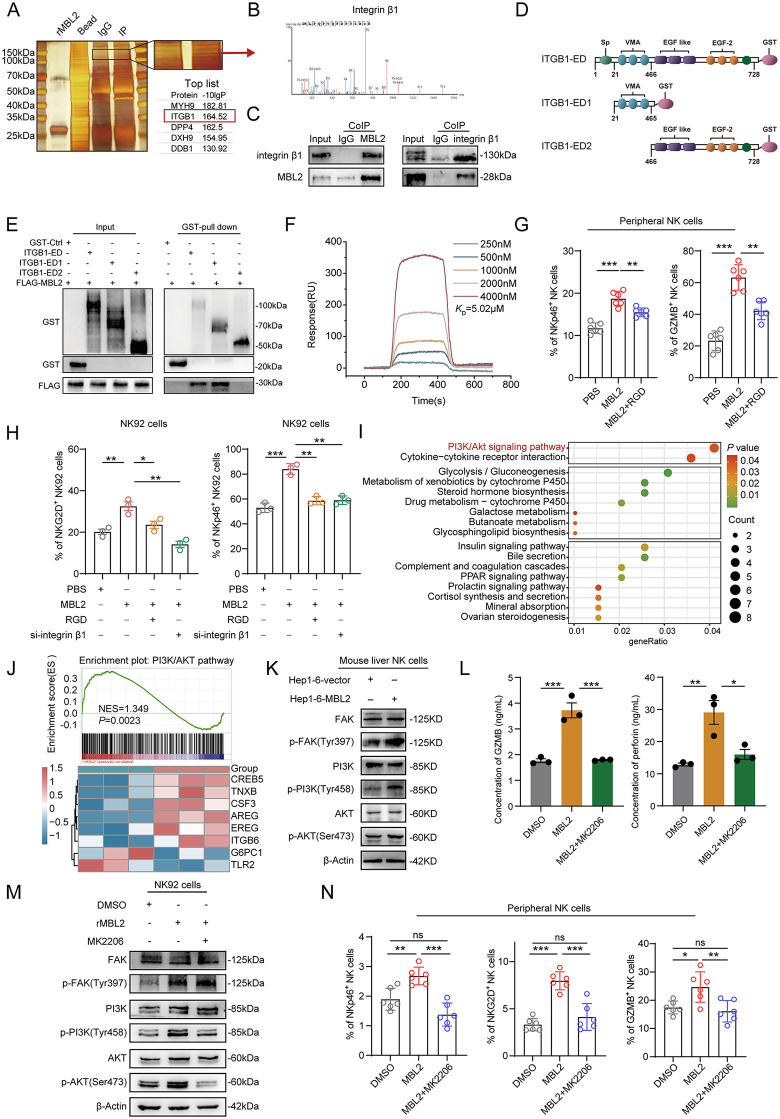
MBL2 activates the PI3K/AKT pathway in NK cells through the integrin β1 receptor. **(A)** NK92 cells co-cultured with rMBL2 were lysed and subjected to immunoprecipitation with anti-MBL2 antibody or IgG, followed by silver staining. Red arrow represents detected band. **(B)** Integrin β1 mass spectrometry analysis. **(C)** CoIP assays were employed to verify the binding of MBL2 protein to the integrin β1 receptor. **(D)** Schematic illustration of the integrin β1 extracellular segment. **(E)** GST pull-down was conducted to validate mutual binding between MBL2 and the ITGB1-ED1 domain. **(F)** Surface plasmon resonance was used to assess the affinity between MBL2 and the ITGB1-ED1 domain. **(G)** Flow cytometry indicated that Arg-Gly-Asp (RGD) reversed MBL2-induced upregulation of the NKp46 receptor and GZMB secretion in NK cells. **(H)** Flow cytometry showed that MBL2 upregulated NKp46 and NKG2D receptors in NK92 cells, which was reversed by RGD and si-ITGB1. **(I)** Enriched differential gene pathways in NK92 cells. **(J)** GSEA results indicated enrichment of gene sets related to the PI3K-AKT signaling pathway in MBL2-stimulated NK92 cells. **(K)** WB demonstrated that MBL2 stimulation activated the PI3K/AKT pathway in mouse hepatic NK cells. **(L)** ELISA revealed that MK2206 inhibited MBL2-induced upregulation of GZMB and perforin secretion in NK92 cells. **(M)** WB confirmed that MK2206 reversed the phosphorylation of AKT caused by MBL2 in NK92 cells. **(N)** Flow cytometry showed that MK2206 completely abrogated MBL2-induced upregulation of NKp46 and NKG2D expression, as well as GZMB production, in peripheral NK cells. All values are shown as mean ± SD. *p < 0.05, **p < 0.01, ***p < 0.001. ns indicates no significance. Source data are available in S1 Data. Abbreviations: ITGB1-ED, integrin β1 extracellular domain; RGD, Arg-Gly-Asp peptide; NES, normalized enrichment score; p-FAK, phosphorylated focal adhesion kinase; p-PI3K, phosphorylated phosphatidylinositol 3-kinase; p-AKT, phosphorylated protein kinase B; MK2206, MK-2206 2HCl, a pan-AKT inhibitor.

To identify the specific receptor with which MBL2 interacts on the NK cell membrane, NK92 cells were co-cultured with rMBL2 for 48 hours. Rapid silver staining revealed a distinct band at 100–150 kDa in the immunoprecipitation (IP) group, which was absent in the IgG control (Fig 3A). Subsequent combined mass spectrometry analysis identified integrin β1 as an MBL2 receptor (Fig 3B). Co-IP assays in NK cells further demonstrated the mutual interaction between MBL2 and integrin β1 (Fig 3C). Gene Ontology pathway enrichment analysis was conducted for membrane proteins binding to MBL2, and the results were visualized through gene-concept networks and clustered dendrograms (S4A–S4C Fig). To clarify the specific domain of the integrin β1 receptor that interacts with MBL2, the extracellular domain (ED, a.a. 1–728) of integrin β1 was divided into two GST-tagged truncated fragments: ITGB1-ED1 (a.a. 21–465), which comprises the VMA domain, and ITGB1-ED2 (a.a. 466–728), which contains the EGF-like and EGF2 domains (Fig 3D). The GST pull-down assay demonstrated that Flag-MBL2 was pulled down by both ITGB1-ED1 and ITGB1-ED, but not by ITGB1-ED2 (Fig 3E). Furthermore, surface plasmon resonance illustrated the binding affinity of MBL2 to the VMA domain with K_D_ = 5.02 μM (Fig 3F). This suggests that MBL2 may interact with integrin β1 through the VMA domain, thereby regulating NK cell function.

To determine whether MBL2 activated NK cells via the integrin β1 receptor, rMBL2 was supplemented to NK cells, with Arg-Gly-Asp (RGD), a specific integrin β1–blocking peptide, used for inhibition. The results indicated the enhancement of NKp46 and GZMB expression on NK cells by MBL2, which was reversed by RGD (Figs 3G and S4D, S4E). Moreover, suppression of NK92 cell activity by RGD and si-ITGB1 (small interfering RNA targeting integrin β1) reversed the MBL2-induced increase in the proportions of NKG2D^+^ and NKp46^+^ NK cells, confirming the MBL2-induced activation of NK cells via the integrin β1 receptor (Fig 3H). We performed high-throughput transcriptome sequencing on NK92 cells stimulated with rMBL2 or PBS control to explore the mechanism underlying MBL2-induced activation of NK cells (S5A Fig). Enrichment analysis revealed differential gene enrichment in the PI3K/AKT pathway (Fig 3I). Meanwhile, gene set enrichment analysis (GSEA) showed that the PI3K/AKT pathway (NES = 1.349) was upregulated in NK92 cells after MBL2 stimulation (Fig 3J). The GSE183349 dataset, which profiles gene expression in tumor-infiltrating NK cells from patients with HCC, was used to perform correlation analysis between integrin β1 and canonical pathways within HCC-infiltrating NK cells. The results showed a strong positive correlation (R = 0.62) between integrin β1 expression and the PI3K/AKT pathway in HCC-infiltrating NK cells (S5B Fig).

To validate MBL2-induced activation of the PI3K/AKT pathway in NK cells, mouse hepatic NK cells were co-cultured with Hepa1–6-MBL2 cells. WB analysis revealed increased phosphorylation of FAK, PI3K, and AKT in NK cells following co-culture with Hepa1–6-MBL2 cells (Fig 3K). Furthermore, ELISA results showed that MK2206, a pan-AKT inhibitor, counteracted the MBL2-induced secretion of GZMB and perforin in NK92 cells, suggesting that MBL2 enhanced NK cell cytotoxicity via the PI3K/AKT pathway (Fig 3L). In parallel, WB analysis of MK2206-treated NK92 cells confirmed that MK2206 reversed the MBL2-induced increase in phosphorylated AKT (p-AKT) levels (Fig 3M). In human peripheral NK cells treated with MBL2, the addition of MK2206 reversed the MBL2-induced increase in the proportions of NKG2D⁺ and NKp46⁺ NK cells, as revealed by flow cytometry. Similarly, we found that MK2206 completely abrogated the intracellular production of GZMB induced by MBL2 in peripheral NK cells following cytokine secretion blockade (Figs 3N and S5D, S5E). These results indicated that MBL2 promoted NK cell activation and cytotoxicity through integrin β1-mediated activation of the PI3K/AKT pathway, thereby enhancing immunosurveillance in HCC.

### 2.4. MBL2 regulates NK-secreted cytokines to inhibit HCC cell progression and reduce the exhaustion of cytotoxic T lymphocytes

**Fig 4:**
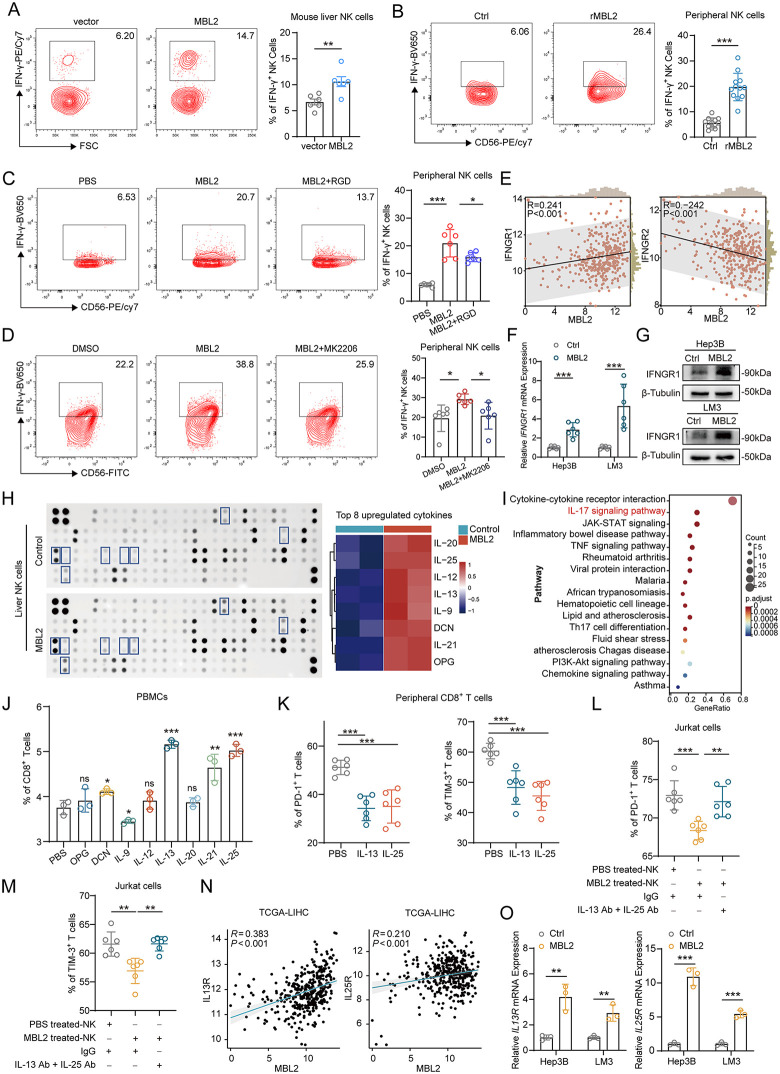
MBL2 regulates NK-secreted cytokines to inhibit HCC progression and reduce the accumulation of exhausted cytotoxic T lymphocytes (CTLs). Flow cytometry was employed to **(A)** assess the secretion of interferon (IFN)-γ in mouse liver NK cells after cultivation with MBL2; (B) assess the MBL2-induced secretion of IFN-γ in peripheral NK cells; and (C, D) reveal that RGD and MK2206 reversed the upregulation of IFN-γ secretion induced by MBL2 in NK cells. **(E)** Correlation analysis between MBL2 and IFNGRs (IFNGR1 and IFNGR2) in the TCGA-LIHC dataset was performed using the Linkedomics portal. (F, G) qPCR and WB were used to assess IFNGR1 expression in Hep3B and LM3 cells overexpressing MBL2 at both the transcriptional and translational levels. **(H)** Mouse cytokine array analysis was conducted to explore secreted cytokines in NK cells upon stimulation with MBL2; the top eight most significant cytokines are listed. **(I)** The IL-17 signaling pathway was enriched by KEGG pathway analysis. Flow cytometry was employed to assess (J) the proportion of CTLs within CD3^+^ T cells stimulated with eight cytokines in NK cell-conditioned media. (K) The inhibitory effect of IL-13 and IL-25 on exhausted CTLs; (L, M) the expression of PD-1 (L) and TIM-3 (M) on Jurkat cells cultured with concentrated supernatants from MBL2-treated NK92 cells, with or without IL-13 and IL-25 neutralizing antibodies (IL-13 Ab and IL-25 Ab). **(N)** The Linkedomics portal was used to perform correlation analysis between MBL2 and IL-13R or IL-25R in the TCGA-LIHC dataset. (O) qPCR was conducted to evaluate IL-13R and IL-25R mRNA expression at the transcriptional level in Hep3B and LM3 cells overexpressing MBL2. All values are shown as mean ± SD. *p < 0.05, **p < 0.01, ***p < 0.001. ns indicates no significance. Source data are available in S1 Data. Abbreviations: PBMCs, peripheral blood mononuclear cells; TCGA-LIHC, The Cancer Genome Atlas–liver HCC dataset.

IFN-γ, a cytokine discharged by NK cells, plays a pivotal role in TME regulation. After co-culture with the Hepa1–6-MBL2 group, the IFN-γ secretion of mouse liver NK cells was significantly increased relative to that after co-culture with the Hepa1–6-vector group (Fig 4A). Likewise, peripheral NK cells were cultured with rMBL2. Flow cytometry showed that MBL2 notably increased IFN-γ secretion in NK cells (Fig 4B). To investigate whether the PI3K/AKT pathway was involved in the promotion of IFN-γ secretion by MBL2 in NK cells, IFN-γ expression was detected in MBL2-stimulated NK cells after RGD blockade and MK2206 inhibition. Flow cytometry revealed that MBL2 increased the frequency of IFN-γ⁺ NK cells within the CD3^–^CD56^+^ NK cell population, whereas both RGD (Fig 4C) and MK2206 (Fig 4D) partially or completely counteracted this upregulation, indicating that MBL2 promoted IFN-γ production through the integrin β1-PI3K/AKT pathway.

Crucially, IFN-γ interacts with the IFNGR1 and IFNGR2 receptors in the HCC microenvironment to exert anti-tumor effects. Here, the Linkedomics platform revealed a positive correlation between MBL2 and IFNGR1 but a negative correlation between MBL2 and IFNGR2 in the TCGA-LIHC dataset (Fig 4E). qRT-PCR and WB were performed to evaluate the expression of IFNGRs facilitated by MBL2 in HCC cells. These results demonstrated that MBL2 upregulated the expression of IFNGR1 in HCC both transcriptionally and translationally (Fig 4F and 4G), whereas *IFNGR2* mRNA expression in HCC was decreased by MBL2 (S5C Fig). In addition to promoting IFN-γ secretion from NK cells, MBL2 enhanced the expression of the IFNGR1 receptor on the membrane of HCC cells, thereby increasing their sensitivity to IFN-γ and establishing a positive feedback loop within the TME that amplifies antitumor immunity.

To investigate the regulation of MBL2 on NK cell–derived cytokines involved in remodeling the immune-activated TME, a cytokine array was performed to detect cytokines released into the supernatants of MBL2-stimulated mouse liver NK cells. In addition to promoting IFN-γ expression, MBL2 was found to enhance the secretion of multiple cytokines from NK cells. The eight most prominently upregulated cytokines were osteoprotegerin (OPG), IL-21, decorin (DCN), IL-9, IL-13, IL-12, IL-25, and IL-20 (Fig 4H). KEGG pathway analysis showed that differentially secreted cytokines were enriched in the IL-17 signaling pathway (Fig 4I). Subsequently, the eight upregulated cytokines were assessed for their regulatory effects on cytotoxic T lymphocytes (CTLs) cultured in NK cell-conditioned media. Among these, IL-13 and IL-25 (also known as IL-17E) exhibited the most pronounced effect in augmenting the proportion of CTLs within the CD3^+^ T cell population (Fig 4J). To further characterize the effects of IL-13 and IL-25 on CTL exhaustion, we cultured CTLs with IL-13 and IL-25 and examined the expression of inhibitory receptors PD-1 and TIM-3 on CTLs. As shown in Figs 4K and S5F, S5G, PD-1^+^ CTLs and TIM-3^+^ CTLs were significantly decreased in the IL-13 and IL-25 groups compared with the PBS group, suggesting that IL-13 and IL-25 secreted by NK cells suppressed the infiltration of exhausted CTLs. To verify the necessity of IL-13 and IL-25 in maintaining low exhaustion levels of CTLs under MBL2-activated NK cell conditions, Jurkat cells, a human T lymphocyte cell line, were cultured with concentrated supernatants from MBL2-treated NK92 cells. Flow cytometry was used to assess PD-1 and TIM-3 expression on Jurkat cells to evaluate whether neutralizing antibodies against IL-13 (IL-13 Ab) and IL-25 (IL-25 Ab) could mitigate the inhibitory effect of MBL2-activated NK cells on exhausted T cells. The results revealed that NK92 cells activated by MBL2 reduced the expression of PD-1 (Figs 4L and S5H) and TIM-3 (Figs 4M and S5I) on Jurkat cells, whereas the addition of IL-13 Ab and IL-25 Ab led to a marked restoration of PD-1 and TIM-3 expression. The Linkedomics platform further displayed a positive correlation between MBL2, IL13R, and IL25R (IL17RB) in the TCGA-LIHC dataset (Fig 4N). Moreover, qPCR of IL13R and IL25R mRNAs in Hep3B and LM3 cells overexpressing MBL2 corroborated that MBL2 upregulated IL13R and IL25R transcriptionally (Fig 4O). These findings suggested that MBL2 amplified IFN-γ secretion by NK cells, activating the inhibitory IFN-γ–IFNGR1 axis against HCC. Furthermore, MBL2 indirectly alleviated CTL exhaustion by promoting IL-13 and IL-25 production in NK cells, thereby facilitating the transition of HCC from an immunosuppressive to an immune-activated state.

### 2.5. MBL2 increases PD-L1 expression in the HCC microenvironment and enhances the effectiveness of anti-PD-L1 immunotherapy

**Fig 5:**
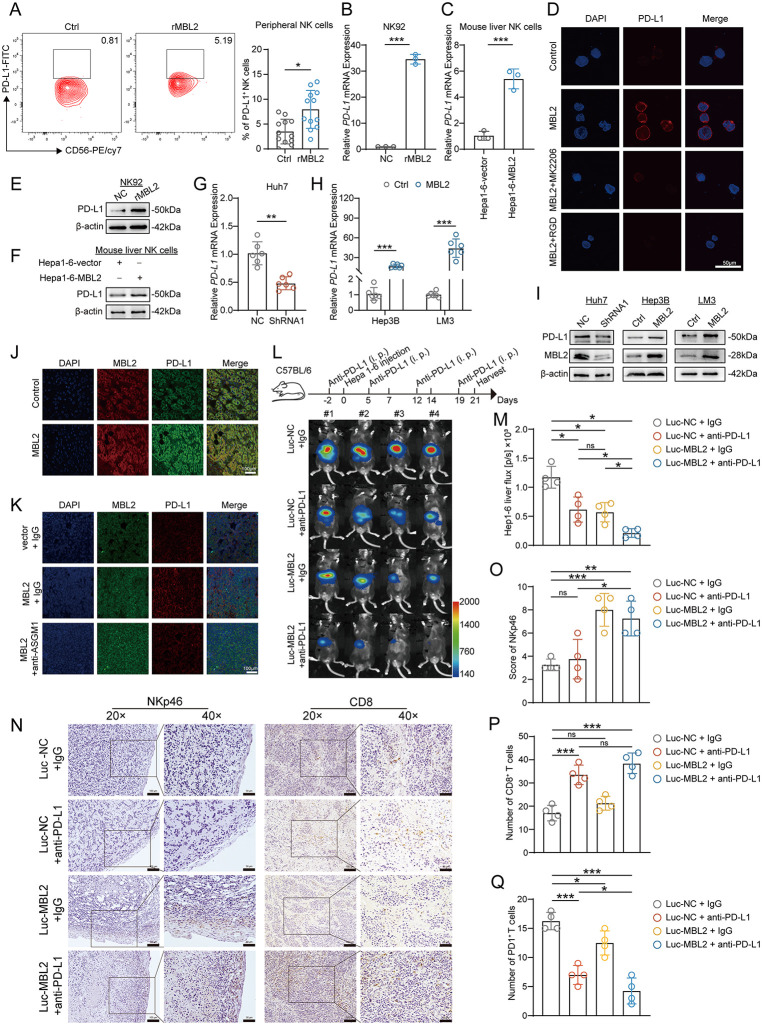
MBL2 increases PD-L1 expression in the HCC microenvironment and enhances the effectiveness of anti-PD-L1 immunotherapy. **(A)** Flow cytometry was used to evaluate PD-L1 expression in peripheral NK cells following cultivation with rMBL2. **(B, C)** qPCR was used to evaluate PD-L1 expression at the transcriptional level in NK92 cells and mouse liver NK cells following treatment with human rMBL2 or co-culture with murine Hepa1-6-MBL2 cells, respectively. **(D)** Immunofluorescence results indicated that MBL2 promoted PD-L1 expression on the membrane of NK92 cells, whereas RGD and MK2206 inhibited PD-L1 expression. The scale bar represents 50 μm. **(E, F)** WB was performed to determine PD-L1 expression in NK92 cells (E) and mouse liver NK cells (F) after cultivation with human rMBL2 or co-culture with murine Hepa1-6-MBL2 cells, respectively. **(G, H)** qPCR was employed to assess PD-L1 mRNA expression in MBL2-knockdown Huh7-shRNA1 cells (G) and in MBL2-overexpressing Hep3B and LM3 cells (H). **(I)** WB was performed to evaluate PD-L1 expression in Huh7 cells with MBL2 knockdown and in Hep3B and LM3 cells overexpressing MBL2. **(J)** Immunofluorescence analysis of PD-L1 expression in clinical HCC tissues with high (MBL2-High) and low (MBL2-Low) MBL2 expression. Scale bar: 100 μm. **(K)** Immunofluorescence analysis of PD-L1 expression in mouse subcutaneous HCC tissues following NK cell depletion using an anti-asialo GM1 (anti-ASGM1) antibody. Scale bar: 100 μm. **(L)** A mouse orthotopic HCC model demonstrated the synergistic effect between MBL2 and anti-PD-L1 therapy. The schematic representation of the experimental procedure is shown at the top. **(M)** Statistical chart of tumor size, examined by a small animal live imaging system. **(N)** Representative NKp46 and CD8 staining images of mouse HCC tissues. Scale bars represent 100 μm at 20 × magnification and 50 μm at 40× magnification. **(O–Q)** IHC was used to assess (O) the infiltration score of NKp46⁺ NK cells, (P) the number of CD8⁺ T cells, and (Q) the expression of PD-1 in the mouse orthotopic HCC model. All values are shown as mean ± SD. *p < 0.05, **p < 0.01, ***p < 0.001. ns indicates no significance. Source data are available in S1 Data. Abbreviations: RGD, Arg-Gly-Asp peptide; MK2206, MK-2206 2HCl, a pan-AKT inhibitor.

PD-L1^+^ NK cells in patients with PD-L1^−^ HCC may enhance the efficacy of anti-PD-L1 therapy and exhibit stronger anti-tumor activity than PD-L1^−^ NK cells. Surprisingly, flow cytometry revealed that rMBL2 upregulated PD-L1 expression on the surface of peripheral NK cells (Fig 5A). Afterwards, NK92 cells were cultured with human rMBL2, and subsequent qPCR and WB analyses showed an upregulation of PD-L1 expression on NK92 cells at both the transcriptional and translational levels (Fig 5B and 5E). Likewise, we examined PD-L1 expression in mouse liver NK cells within the murine HCC-NK co-culture system. qPCR and WB analyses revealed significantly elevated PD-L1 expression in NK cells co-cultured with Hepa1–6-MBL2 cells compared to those co-cultured with Hepa1–6-vector cells (Fig 5C and 5F). To further explore whether MBL2 also mediated PD-L1 expression on NK cells through triggering activation of the PI3K/AKT pathway, immunofluorescence assays were conducted to estimate the inhibitory effects of MK2206 and RGD on the upregulation of the PD-L1 receptor on NK cells by MBL2. The results showed that the expression of PD-L1 in the MBL2 group was significantly increased, whereas PD-L1 was markedly repressed by the addition of RGD peptide. Similarly, PD-L1 on NK cell membranes was downregulated after the inclusion of MK2206 (Fig 5D). Thus, MBL2 can enhance the proportion of PD-L1^+^ NK cells, improving the efficacy of anti-PD-L1 treatment in HCC.

The IFN-γ–IFNGR1 axis is involved in the induction of PD-L1 expression in tumor cells. To investigate whether secreted MBL2 regulated PD-L1 expression in HCC cells via the IFN-γ–IFNGR1 axis, we examined PD-L1 expression in MBL2-knockdown Huh7-shRNA1 cells (Fig 5G), following co-culture with NK cells, as well as in Hep3B and LM3 cells overexpressing MBL2 (Fig 5H). WB analysis demonstrated that PD-L1 expression was reduced in MBL2-knockdown Huh7-shRNA1 cells in parallel with decreased MBL2 levels, whereas PD-L1 expression was elevated in Hep3B-MBL2 and LM3-MBL2 cells (Fig 5I). From a histological perspective, we performed immunofluorescence staining on clinical HCC specimens with high (MBL2-High) and low (MBL2-Low) MBL2 expression. PD-L1 expression was significantly elevated in the MBL2-High group compared to the MBL2-Low group (Fig 5J). Considering the importance of NK cell-derived IFN-γ in PD-L1 upregulation, we assessed PD-L1 expression in mouse subcutaneous HCC tissues following NK cell depletion, as shown in Fig 2L. Immunofluorescence assays uncovered that NK cell depletion reduced PD-L1 expression in HCC tissues (Fig 5K), indicating that NK cell–derived IFN-γ plays an essential role in MBL2-mediated promotion of PD-L1 expression.

A mouse orthotopic HCC model was established, which was administered with an anti-PD-L1 monoclonal antibody intraperitoneally to assess the synergistic therapeutic potential of MBL2 and anti-PD-L1 therapy (Fig 5L). In vivo bioluminescence imaging results showed that both anti-PD-L1 monotherapy (Luc-NC + anti-PD-L1) and high expression of MBL2 (Luc-MBL2 + IgG) could effectively inhibit the growth of HCC. However, compared with anti-PD-L1 monotherapy, the group with high expression of MBL2 in combination with anti-PD-L1 treatment (Luc-MBL2 + anti-PD-L1) showed a significant decrease in tumor volume (Fig 5M). IHC assays were utilized to evaluate the expression of NKp46 and CD8 in mouse HCC tissues (Fig 5N). The results showed no difference in NKp46^+^ NK cells between the Luc-NC + anti-PD-L1 group and the control group. However, the infiltration of NKp46^+^ NK cells was significantly increased in the Luc-MBL2 + anti-PD-L1 group compared to the Luc-NC + anti-PD-L1 group. This suggested that elevated expression of MBL2 increased the infiltration of NK cells during anti-PD-L1 treatment (Fig 5O). CD8^+^ T cell infiltration was increased in the Luc-MBL2 + anti-PD-L1 group compared with the control group (Fig 5P). Although there was no significant difference in CD8⁺ T cell infiltration between the Luc-MBL2 + anti-PD-L1 and Luc-NC + anti-PD-L1 groups, PD-1 expression was markedly diminished in the Luc-MBL2 + anti-PD-L1 group (Figs 5Q and S6A). These findings demonstrated a synergistic effect between MBL2 restoration and anti-PD-L1 treatment, which suggested that clinical supplementation of MBL2 may slow down the progression of HCC, and HCC patients with high MBL2 expression may derive greater therapeutic benefit from anti-PD-L1 therapy.

### 2.6. Hepatitis B virus X protein downregulates MBL2 expression in HCC

**Fig 6:**
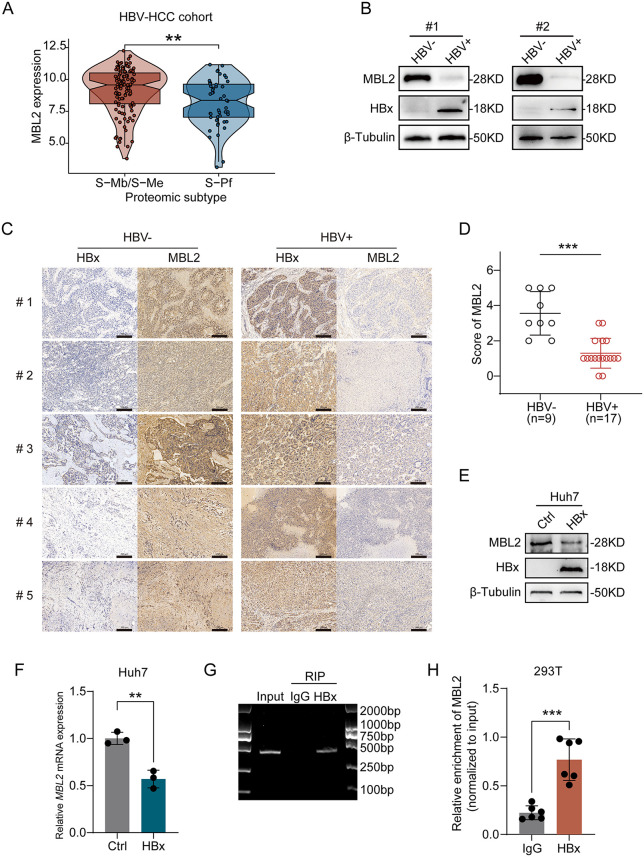
Hepatitis B virus X protein downregulates MBL2 expression in HCC. **(A)** Differential expression analysis of MBL2 in S-Mb/S-Me and S-Pf subtypes was conducted in the HBV-HCC (OEP000321) dataset. **(B)** WB analysis was performed to measure MBL2 expression in clinical HBV(+) HCC tissues compared to paired HBV(–) HCC tissues. **(C)** IHC staining was performed to evaluate the expression of MBL2 in clinical HCC tissues, comparing HBV(+) and HBV(–) HCC tissues. The scale bar represents 200 μm. **(D)** Quantitative analysis of IHC staining intensity was conducted to illustrate the relative IHC scores for MBL2 expression. **(E, F)** WB (E) and qPCR (F) were used to evaluate the expression of MBL2 in Huh7 cells following HBx-His plasmid transfection. **(G, H)** PCR (G) and qPCR (H) were employed to validate the RIP assay, confirming the binding of HBx to MBL2 mRNA. All values are shown as mean ± SD. **p < 0.01, ***p < 0.001. Source data are available in S1 Data. Abbreviations: HBV-HCC, hepatitis B virus-related HCC; S-Mb, the metabolism subgroup; S-Me, the microenvironment-dysregulated subgroup; S-Pf, the proliferation subgroup; HBx, hepatitis B virus X protein; RIP, RNA immunoprecipitation.

An integrated proteogenomic analysis of an HBV-associated HCC cohort identified three molecular subgroups: the metabolism subgroup (S-Mb), the microenvironment-dysregulated subgroup (S-Me), and the proliferation subgroup (S-Pf). These subtypes exhibited distinct molecular features related to cell proliferation, metabolic reprogramming, microenvironmental dysregulation, and potential therapeutic vulnerabilities. Among them, S-Pf was distinguished by elevated expression of proliferative proteins and enrichment of therapeutic targets, in contrast to S-Mb and S-Me. Bioinformatic analysis revealed that MBL2 expression in S-Pf was significantly downregulated compared to that in S-Mb and S-Me (Fig 6A). WB analysis indicated that MBL2 expression in the HBV(+) group was downregulated compared to that in the HBV(−) group (Fig 6B). MBL2 expression in HBV(−) and HBV(+) HCC tissues was assessed using IHC (Fig 6C). Statistical analysis using the rank-sum test revealed a significant (*p* < 0.001) decrease in MBL2 protein expression in the HBV(+) group (Fig 6D). Hepatitis B virus X protein (HBx), a multifunctional regulatory protein encoded by HBV, has been extensively implicated in the initiation and progression of HCC. To further elucidate the relationship between HBx and MBL2, an HBx-His plasmid was constructed and transfected into Huh7 cells. WB and qPCR analyses consistently demonstrated that overexpression of HBx markedly reduced MBL2 levels in Huh7 cells (Fig 6E–6F), suggesting that HBx negatively regulates MBL2 expression at the post-transcriptional level. To address the binding of HBx protein to MBL2 mRNA, RNA immunoprecipitation (RIP) assays were performed in 293T cells. Using HBx protein as the immunoprecipitating target, both PCR (Fig 6G) and qPCR (Fig 6H) analyses confirmed the enrichment of MBL2 mRNA in HBx-containing complexes, thereby providing direct evidence that HBx physically associates with MBL2 transcripts. Collectively, these findings indicated that HBx may suppress MBL2 expression by binding to MBL2 mRNA, highlighting that HBV does not merely repress MBL2 expression at the genomic or transcriptional stage, but also promotes MBL2 degradation or destabilization at the post-transcriptional level. Importantly, restoring MBL2 expression may provide synergistic benefits when combined with immune checkpoint blockade, offering a promising approach to improve the efficacy of anti-PD-L1–based immunotherapies in patients with HBV-related HCC.

### 2.7. MBL2 expression is markedly decreased in HCC and correlated with HCC prognosis

**Fig 7:**
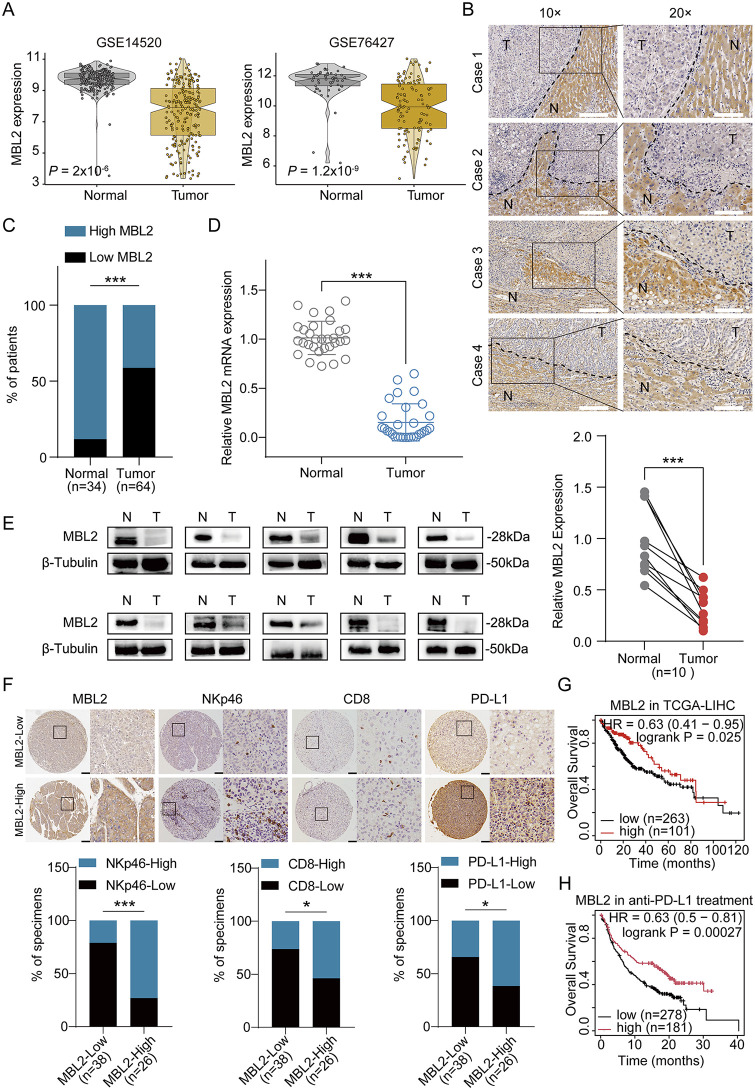
MBL2 expression is markedly decreased in HCC and correlates with the prognosis of HCC. **(A)** Analysis of MBL2 expression in HCC tissues compared with adjacent normal tissues in the HCC microarray profiles GSE14520 and GSE76427. **(B)** Representative MBL2 IHC staining images of adjacent normal tissues and tumor tissues. **(C)** Percentages of samples with high and low expression of MBL2 in the HCC tissue array. **(D)** qPCR analysis of MBL2 mRNA expression in 10 human HCC tissues and matched adjacent normal tissues from the same patients. **(E)** Relative MBL2 protein expression in 10 primary HCC tissues and adjacent normal tissues was determined using WB. **(F)** IHC was performed to examine HCC tissues from the MBL2-High group and MBL2-Low group on HCC tissue microarrays. The expression and correlation of NKp46, CD8, and PD-L1 in human HCC tissues were detected. Two representative cases are shown. The scale bar represents 100 μm. Percentages of patients with HCC exhibiting high or low expression of the indicated factors are shown below. **(G, H)** Kaplan–Meier survival analyses of overall survival (OS) based on MBL2 expression in the TCGA-LIHC dataset (G) and an anti-PD-L1 treatment cohort (H), split according to the auto-selected optimal cutoff value. All values are shown as mean ± SD. *p < 0.05, ***p < 0.001. Source data are available in S1 Data.

Based on the GEO database, two microarray datasets were selected to juxtapose the expression of MBL2 in HCC and paired normal tissues (Fig 7A). To provide a comprehensive histological perspective, IHC was performed on an HCC tissue microarray to evaluate MBL2 expression in normal and HCC tissues (Fig 7B). IHC analysis of HCC tissues paired with adjacent normal liver tissues revealed significantly elevated expression of MBL2 in liver tissues compared with that in HCC tissues (Fig 7C). The outcome of this analysis confirmed a substantial downregulation of MBL2 expression in HCC tissues compared with normal counterparts. These findings underscored the visible reduction in patient samples exhibiting high MBL2 expression in tumor tissues. Subsequently, we recruited a cohort of 10 patients with HCC who underwent resection surgery. Both HCC and adjacent normal tissues were collected. The specimens were subjected to qPCR and WB to assess MBL2 expression at the transcriptional and translational levels, respectively. The qPCR results displayed that *MBL2* mRNA expression was significantly reduced in HCC tissues compared with the adjacent normal tissues (Fig 7D). The WB results demonstrated a significant decrease in MBL2 expression in HCC tissues compared with that in adjacent normal tissues (Fig 7E). IHC staining of the tissue microarray of clinical patients with HCC further indicated that the expression of MBL2 was positively correlated with the expression of NKp46 and PD-L1, and further correlated with CD8^+^ T cell infiltration (Fig 7F). Kaplan–Meier survival analysis revealed that high expression of MBL2 was associated with a favorable prognosis for HCC in the TCGA-LIHC dataset (Fig 7G). Analogous differences in progression-free survival (PFS), disease-specific survival (DSS), and recurrence-free survival (RFS) were also observed (S6B–S6D Fig). Concurrently, elevated MBL2 expression exhibited a substantial augmentation in the overall survival (OS) of patients diagnosed with tumors who had undergone anti-PD-L1 treatment (Fig 7H). In conclusion, the reduction of MBL2 in HCC tissues and its association with a favorable prognosis suggest its potential as a prognostic biomarker and therapeutic target in HCC. Furthermore, MBL2-induced NK cell activation combined with anti-PD-L1 antibody therapy can greatly enhance antitumor efficacy and provide a promising strategy for improving the efficacy of immunotherapy in HCC.

## 3. Discussion

**Fig 8:**
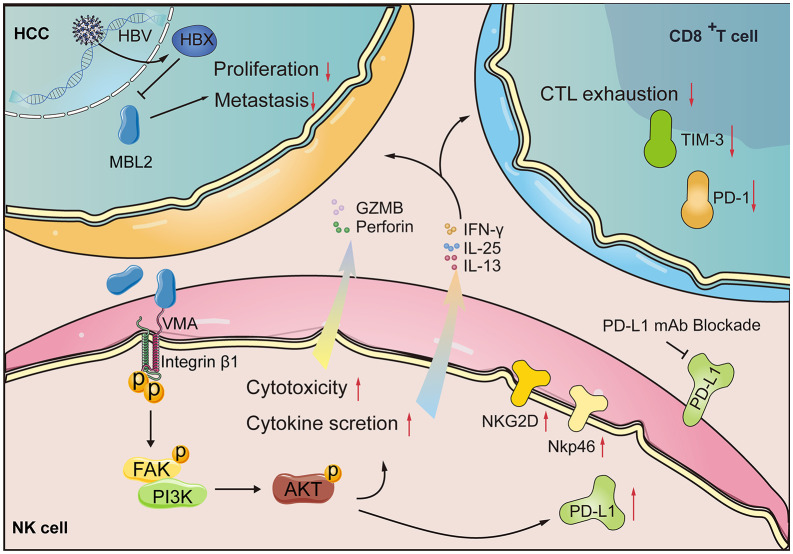
Schematic illustration of the antitumor mechanism of MBL2 in the HCC TME. Endogenous MBL2 can inherently suppress the proliferation and migration of HCC cells. Secreted MBL2 activates the PI3K/AKT pathway through the integrin β1 receptor, leading to the recruitment and activation of NK cells in the HCC microenvironment. Concurrently, MBL2 stimulates the secretion of cytokines such as IL-13, IL-25, and IFN-γ by NK cells, diminishing T cell exhaustion and remodeling the immunosuppressive HCC microenvironment into an immunostimulatory state. Furthermore, MBL2 elevates the expression of PD-L1 on both HCC and NK cells.

Tumor-secreted proteins significantly affect the TME [10]. Therefore, comprehensive analyses of the interactions between tumor cells and immune cells within the TME, as well as the regulatory mechanisms governing these secreted proteins, are crucial for identifying critical components of the tumor immune evasion process [23–25]. In this study, we found that hepatocyte-derived MBL2 recruited and activated NK cells through the PI3K/AKT pathway and upregulated the expression of PD-L1 on NK cells within HCC (Fig 8). These findings suggest that restoring MBL2 may be a promising therapeutic approach for HCC that could enhance the effectiveness of anti-PD-L1 immunotherapy.

Crucially, MBL2 polymorphisms can affect viral clearance and suppress viral replication in individuals with viral infections [26,27]. Furthermore, MBL2 variations that reduce protein expression or functionality may increase the risk of HCC [28–30]. Previous studies have focused on the clinical impact of *MBL2* polymorphisms in HBV infection [31]. However, we addressed the molecular mechanism by which HBx protein downregulates MBL2 post-transcriptionally, demonstrating a multifaceted regulatory effect of HBV on HCC. HBx has been shown to interact with and relocalize the m⁶A writer complex (METTL3/METTL14 with WTAP), guiding co-transcriptional m⁶A deposition on HBV RNAs from cccDNA [32]. Importantly, HBx can also increase m⁶A on host transcripts, exemplified by PTEN, resulting in reduced RNA stability and protein levels [33]. HBx likely recruits the m⁶A writer complex to deposit site-specific m⁶A within the MBL2 3′UTR, thereby reducing MBL2 mRNA stability, protein abundance, and secretion. Once the precise methylation landscape of MBL2 has been delineated, combinatorial regimens incorporating MBL2 supplementation may offer therapeutic benefit in HBV-related HCC.

NK cells play crucial immunoregulatory roles in the HCC microenvironment [34]. However, inhibitory factors produced by HCC and other immune cells, such as tumor-associated growth factors and immunosuppressive cytokines, can compromise NK cell functionality [35]. To robustly model this pathological state in vitro, we subjected isolated NK cells to a 72-hour *ex vivo* culture maintained with a restricted dose of IL-15 (20 ng/mL). This prolonged, low-cytokine conditioning intentionally suppresses basal NK cell activation, thereby establishing a low-expression baseline that partially recapitulates HCC-induced exhaustion. This specific baseline was methodologically essential for our functional assays, enabling us to distinctly quantify the restorative effects of MBL2 on NK cell activity, which would otherwise be obscured in freshly isolated, highly reactive cells. Interestingly, given the high structural conservation of MBL2 between humans and mice [36], we observed that secreted MBL2 successfully recruited NK cells in the nude mouse subcutaneous tumor model. To address any potential interspecies discrepancies regarding NK cell functionality, we further validated MBL2-induced NK cell activation utilizing both human and murine hepatic NK cell co-culture systems. Collectively, these findings demonstrate that MBL2 not only enhances NK cell recruitment but also promotes their subsequent activation and maturation.

Recognition of major histocompatibility complex class I (MHC-I) molecules mediated by their specific inhibitory receptors is critical for the acquisition of functional effector NK cells [37]. The loss of interaction between MHC-I and the inhibitory Ly49 receptor on NK cells restores the antitumor activity of NK cells [38]. Tumor cells with low expression of MHC-I molecules become highly sensitive to cytotoxicity from NK cells. However, HCC-derived MBL2 regulates NK cell infiltration and activation in an MHC-I-independent manner. MYH9 in NK cells is closely involved in intracellular transport of lytic granules, immunological synapse dynamics, and degranulation, but is not a prototypical extracellular receptor [39]. The immunoprecipitation between MYH9 and MBL2 in Fig 3A more likely reflects intracellular complex formation or indirect association. DHX9 functions as a cytosolic nucleic-acid sensor [40]. DPP4 can be shed to yield soluble DPP4 and is a serine dipeptidyl peptidase that frequently modifies microenvironmental signaling by trimming bioactive peptides [41]. Consequently, the functional outcomes of binding are complex and readily confounded by its soluble form and enzymatic activity. By contrast, integrin β1 is a canonical cell-surface transmembrane receptor responsible for extracellular ligand engagement and transmembrane signaling, which mediate processes such as cell adhesion, extracellular matrix remodeling, immune cell activation, and tumor progression [42,43]. This property naturally positions integrin β1 as an optimal membrane binding target and signaling entry point for HCC-derived MBL2.

In addition to conventional NK cells that originate in the bone marrow and migrate through the circulation, liver-resident NK (Lr-NK) cells show distinct phenotypic and functional characteristics compared to circulating and splenic NK cells [44,45]. Lr-NK cells are characterized by integrin α1 (CD49a, VLA-1), which binds basement-membrane collagen IV and laminin to regulate localization and differentiation [46]. Within an integrin α1-paired context, the engagement of integrin β1 by MBL2 is poised to enhance Lr-NK cell recruitment and response strength within HCC. From this perspective, sufficient MBL2 would be expected to efficiently sustain the residency and activation of Lr-NK cells, whereas diminished MBL2 (e.g., during HBV infection) could attenuate CD49a-dependent residency and weaken hepatic immune surveillance. Thus, a deeper investigation into how MBL2 regulates Lr-NK cell localization and activation is warranted, as this may provide new therapeutic avenues to enhance antitumor immunity and improve immunotherapeutic outcomes in HCC.

While previous scRNA-seq identified a subpopulation of integrin β1^+^ Tregs with increased sensitivity to α-PD-1 treatment [47], our results confirmed that MBL2 activates NK cells through the integrin β1 receptor. After MBL2 treatment, genes related to the PI3K/AKT pathway were enriched in NK cells. The PI3K/AKT pathway regulates NK cell activation and cytotoxicity [48]. Activation of the PI3K/AKT pathway enhances NK cell activity, promoting the ability of NK cells to recognize and kill HCC cells [49]. Moreover, AKT activation results in the release of effector cytokines, including IFN-γ, which exert potent antitumor effects [50]. The AKT inhibitor MK2206 suppressed the expression of effector and secreted proteins in MBL2-activated NK cells, leading to an immunosuppressive state in the immune microenvironment. Ultimately, activation of the integrin β1/PI3K/AKT pathway in NK cells plays a crucial role in reshaping an immune-activated microenvironment.

IFNs exert powerful anti-tumor effects by regulating tumor cell proliferation, inhibiting tumor metastasis and neovascularization, and activating immune responses [8,51]. Furthermore, they have demonstrated significant clinical efficacy in the treatment of melanoma, malignant lymphoma, and renal cancer [8,52]. We found that MBL2 significantly promotes IFN-γ secretion from NK cells and IFNGR1 expression in HCC cells. Consequently, apoptosis of HCC cells is promoted through the IFN-γ–IFNGR axis. Additionally, recent studies have reported that the IFN-γ–IFNGR1 axis contributes to the induction of PD-L1 expression in tumor cells [53,54]. Notably, MBL2 promotes PD-L1 expression on the membrane of HCC cells in an IFN-γ-dependent manner. Therefore, the molecular mechanisms by which MBL2 promotes PD-L1 expression in HCC cells require further exploration.

NKp46^+^ ILC1s in humans and mice displayed more robust cytokine secretion and cytotoxicity than NKp46^−^ ILC1s [55]. The presence of NKp46 on NK cells and ecto-CRT on the tumor boosted the functionality of tumor-infiltrating NK cells. High expression of NKp46 on liver NK cells has been associated with a better outcome [56]. The first-in-class anti-NKp46 mAb was more potent than anti-NKG2D mAb in activating NK effector functions in vitro [57]. Our study also demonstrated a more pronounced upregulation of NKp46 on MBL2-activated NK cells, suggesting that NKp46 is a promising marker of NK cell activation. Future studies can be focused on elevating the infiltration of NKp46^+^ NK cells to enhance the antitumor effect in HCC.

Although PD-L1 antibody therapy, such as atezolizumab and durvalumab, has demonstrated positive therapeutic effects in certain patients with HCC, its overall efficacy requires further improvement [58]. As demonstrated by Zhu and colleagues, PD-L1-targeted therapy is not universally applicable to all patients with HCC, as only a proportion have HCC cells that express PD-L1 [59]. Recent studies have suggested that PD-L1^+^ NK cells in patients with PD-L1^–^ HCC may enhance anti-PD-L1 therapy and exhibit stronger anti-tumor effects [49]. Increasing PD-L1^+^ NK cell infiltration in HCC may potentially improve the response to anti-PD-L1 therapy. However, the approach to increase PD-L1^+^ NK cell infiltration remains to be investigated. Our results revealed that MBL2 increases PD-L1 expression in both HCC cells and NK cells, synergistically enhancing the sensitivity to anti-PD-L1 therapy in HCC.

Exogenous MBL2 addition did not directly inhibit HCC progression but modulated the extracellular matrix to inhibit HCC growth. However, endogenous elevation of MBL2 showed a direct inhibitory effect on the proliferation and metastasis of HCC cells intracellularly instead of by an autocrine mode. Although the attenuated proliferative capacity of HCC with high expression of MBL2 suggests a better prognosis, restoration of MBL2 indirectly suppresses HCC progression by recruiting and activating infiltrating NK cells, whilst improving the response to anti-PD-L1 therapy in HCC.

In conclusion, endogenous MBL2 suppresses the proliferation and migration of HCC cells, whereas secreted MBL2 activates the PI3K/AKT pathway through the integrin β1 receptor, driving the recruitment and activation of NK cells in the HCC microenvironment. MBL2 concurrently stimulates the secretion of cytokines such as IL-13, IL-25, and IFN-γ by NK cells, which reduces T cell exhaustion and restructures the immunosuppressive HCC microenvironment into an immunostimulatory state, increasing the expression of PD-L1 on NK cells. Therefore, targeted supplementation with MBL2 represents a potential therapeutic strategy for modulating the infiltration and activation status of immune cells, thereby enhancing the antitumor capability of NK cells when combined with anti-PD-L1 immunotherapy.

## 4. Experimental section/methods

### Cell lines and cell culture

The human HCC cell lines (Hep3B, HepG2, Huh7, MHCC97-L (97-L), MHCC97-H (97-H), LM3), NK92 (a human natural killer cell line), Jurkat (a human T lymphocyte leukemia cell line), HEK293T (293T) cell lines and the mouse HCC cell line Hepa1–6, were obtained from the National Collection of Authenticated Cell Cultures (Shanghai, China) with short tandem repeats certifications. The cells were maintained as previously described [60]. All HCC cell lines and 293T cells were cultured in DMEM (#SH30022; HyClone) supplemented with 10% fetal bovine serum (FBS; #FSD500; ExCell Bio) at 37 °C with a humidity of 5% CO_2_. Jurkat cells were maintained in RPMI-1640 medium (#SH30027, HyClone) supplemented with 10% FBS under the same incubation conditions. NK92 cells were cultured in NK-92 cell complete medium (#CM-0530; Procell) supplemented with IL-2 (1 × 10^5^ U/mL) at 37 °C in a 5% CO_2_ incubator.

### Separation and culture of NK cells and HCC-NK cell co-culture

Peripheral blood samples were collected from healthy donors and diluted in 1× PBS. PBMCs were isolated with Ficoll Plus 1.077 (#P4350; Solarbio) and cultured in RPMI-1640 (#SH30027; HyClone) with 10% FBS. Peripheral NK cells were purified from PBMCs with a human NK cell isolation kit (#17955; STEMCELL). Peripheral NK cells were activated with IL-2 (100 U/mL; #200-02, PeproTech) and IL-15 (20 ng/mL; #200-15, PeproTech), while peripheral cytotoxic T lymphocytes (CTLs) were stimulated with IL-2 (100 U/mL) and phytohemagglutinin-P (PHA-P; 2 μg/mL; #CS0007, MultiSciences). Both cell types were seeded into 12-well plates at a density of 1 × 10⁶ cells/mL and cultured for 72 hours. Peripheral NK cells were cultured with rMBL2 (250 ng/mL; Sino Biological) in the experimental group, and with an equal volume of PBS in the control group.

Tumor tissues were minced and digested with collagenase IV (2 mg/mL, Sigma) and DNase I (50 μg/mL, Sigma) for 30 min at 37°C. Single-cell suspensions were filtered through a 70 μm strainer and incubated using mouse NK cell isolation kit (#19855; Stemcell) according to the manufacturer’s instructions. Isolated mouse liver NK cells were cultured in RPMI-1640 medium supplemented with 10% FBS, mouse IL-15 (50 ng/mL; #210-15, PeproTech), and 1% penicillin-streptomycin solution (#P1400; Solarbio).

Human NK92 cells (seeded in the upper chamber) were co-cultured with stably transfected Hep3B, LM3, or Huh7 cells (in the lower chamber) at a 1:3 ratio in NK-92 complete medium supplemented with 10% FBS, 1% penicillin-streptomycin and IL-2 (1 × 10^5^ U/mL) for 48 hours. For the murine co-culture system, mouse liver NK cells (in the lower chamber) were co-cultured with Hepa1−6 cells (in the upper chamber) at a 1:1 ratio in RPMI-1640 medium supplemented with 10% FBS, 1% penicillin-streptomycin and mouse IL-15 (50 ng/mL) for 24 hours. RGD peptides (20 μM, s8008, Selleck) were administered to block the integrin β1 receptor. Inhibition of the PI3K/AKT pathway was achieved using MK2206−2HCl (MK2206, 1 μM, S1078, Selleck) for 48 hours.

### Animals

All animal experiments were approved by the Laboratory Animal Ethics Committee of Zhujiang Hospital of Southern Medical University (Ethics code: LAEC-2023-141) according to the guidelines for the ethical treatment of animals. Animal-related research protocols are in accordance with the U.S. Public Health Service Policy on Humane Care and Use of Laboratory Animals. Male C57BL/6 mice (aged 5–6 weeks, weighing 20–22 g) and male BALB/c nude mice (aged 5–6 weeks, weighing 16–20 g) purchased from the Southern Medical University Experimental Animal Center were housed in a specific-pathogen-free facility with free access to sterilized food and water.

### Subcutaneous tumor measurements and orthotopic HCC cell injections

BALB/c nude mice were randomly divided into three groups. The experimental mouse groups received subcutaneous injections of 1 × 10^6^ LM3 cells overexpressing MBL2 or MBL2^ΔSP^, and the control group received subcutaneous injections of LM3 cells carrying the control vector in equal amounts. On day 16, all subcutaneous tumors were collected, and their volumes and masses were measured.

A total of 1 × 10⁶ Hepa1–6-luc-MBL2 or Hepa1–6-vector cells were subcutaneously injected into C57BL/6 mice to establish a subcutaneous HCC model. For NK cell depletion, mice received intraperitoneal injections of 50 μg anti-asialo GM1 antibody (anti-ASGM1; #146002, BioLegend) twice weekly, starting 1 week prior to tumor implantation. Tumor size was monitored every 3 days until tumor endpoint.

C57BL/6 mice were orthotopically implanted with 5 × 10^5^ luciferase-labeled Hepa1–6-luc-MBL2 or Hepa1–6-vector cells. Blocking antibodies against PD-L1 (#a2115; Selleck) and an IgG isotype control (#a2116; Selleck) were administered once weekly for three weeks at a dose of 12.5 mg/kg. Tumor size was monitored every 7 days using a small animal in vivo imaging system.

### Construction of lentivirus, plasmids and siRNA

A stable lentiviral vector with an overexpression or shRNA sequence of *MBL2* was constructed based on human full-length cDNA by HanBio. To prevent the secretion of MBL2 into the extracellular compartment, we constructed a lentivirus harboring an *MBL2* mutant lacking the signal peptide (SP) sequence. A stable lentiviral vector encoding luciferase-tagged MBL2 was constructed using full-length murine MBL2 cDNA. Transduced HCC cells were selected using 4 μg/mL puromycin for two weeks cultivation. Plasmids encoding HBx-His and MBL2-Flag were constructed using the pcDNA3.1(+) vector and purchased from Kidan Biosciences. Small interfering RNA targeting integrin β1 (si-ITGB1) was synthesized by Kidan Biosciences. The knockdown efficiency of si-ITGB1 has been previously validated [42]. Transfections were performed with Lipofectamine 3000 (#L3000015; Invitrogen) according to the manufacturer’s protocol. The pGEX-4T-1 cloning vectors containing integrin β1 extracellular domain (ED) genes, ITGB1-ED1 (a.a. 21–465) and ITGB1-ED2 (a.a, 465–728) truncations were constructed by Kidan Biosciences. Transfection efficiency was confirmed using qPCR and western blot.

### Western blot analysis

Cell lysates were analyzed by immunoblotting in radioimmunoprecipitation assay buffer (RIPA; #P0013E; Beyotime) containing 1% protease inhibitor to assess protein expression. Protein concentrations were determined using a bicinchoninic acid protein quantification kit (#P0010; Beyotime). Polyvinylidene fluoride membranes (PVDF; #IPVH00010; Merck Millipore) were blocked with 5% bovine serum albumin and incubated for 1 hour at room temperature (RT). The primary antibodies used are listed in S3 Data. Following overnight incubation at 4 °C, the PVDF membranes were rinsed with PBS-Tween 20 and exposed to horseradish peroxidase-conjugated secondary antibodies (1:1000, #7074ᮤ CST) for 1 hour at RT. Images were acquired using a Tanon-5200 chemiluminescent imaging system (Tanon). Three independent experiments were conducted. Western blot band intensities were quantified using ImageJ software (NIH). The raw quantification data and statistical analyses are provided in S1 Data and S4 Data, respectively. Briefly, images were converted to an inverted 8-bit grayscale format, and the ‘Analyze > Gels’ function was utilized to plot lane intensity histograms. To subtract background noise, profile peaks were enclosed with straight baselines. The Wand tool was then used to calculate the integrated density (area) of each peak. Final relative expression levels were determined by normalizing these values against their corresponding loading controls (β-Actin or β-Tubulin). Source data are available in S1 Raw Images.

### Flow cytometry

Flow cytometry was employed to quantify the expression of cell surface markers (NKG2D, NKp46, PD-L1, PD-1, TIM-3) and intracellular cytokines (GZMB, perforin, IFN-γ) in the indicated cell populations. Briefly, cells were washed twice with flow cytometry wash buffer (PBS containing 1% FBS and 0.5 mM EDTA) and incubated with Fc Block (#553,142; BD Pharmingen) at room temperature (RT) for 10 min to prevent nonspecific antibody binding. Approximately 1 × 10⁶ cells were resuspended in 100 μL of staining buffer and stained with Fixable Viability Stain 700 (1:1000; #564997; BD Biosciences) to exclude dead cells. NK92 cell degranulation was assessed by adding anti-CD107a and BFA/Monensin Mixture (5 μL/mL; #CS1002, MultiSciences) at the onset of a 6-hour stimulation period, followed by surface staining. For surface antigen detection, cells were incubated with fluorochrome-conjugated monoclonal antibodies (listed in S3 Data) at 4 °C for 30 min in the dark. For intracellular cytokine detection, NK cell-derived cytokines were stimulated with PMA/Ionomycin Mixture (5 μL/mL; #CS1001, MultiSciences) and their intracellular accumulation was blocked using BFA/Monensin Mixture (5 μL/mL) for 6 hours at 37 °C. After surface staining, cells were fixed and permeabilized using BD Cytofix/Cytoperm Fixation/Permeabilization Kit (#554714, BD Pharmingen) according to the manufacturer’s protocol, followed by intracellular staining with relevant antibodies (listed in S3 Data) at RT for 30 min. All staining steps were performed in staining buffer under light-protected conditions. Data acquisition was conducted using a BD LSRFortessa flow cytometer (BD Biosciences), and data analysis was performed with FlowJo software (Tree star). Fluorescence minus one (FMO) controls and isotype controls were included to ensure gating accuracy and control for nonspecific staining.

### Immunohistochemistry (IHC)

IHC was performed on 2.5 μm sections of formalin-fixed, paraffin-embedded HCC and adjacent noncancerous tissues. Antigen retrieval was conducted using citrate buffer (pH 6.0) or EDTA buffer (pH 8.0). After blocking endogenous peroxidase activity and nonspecific binding, sections were incubated overnight at 4 °C with primary antibodies against MBL2 (1:1000, DF4152; Affinity, Shanghai, China). Detection was performed using an HRP-conjugated secondary antibody and DAB staining kit (Zsbio), followed by nuclear counterstaining with Mayer’s hematoxylin. Slides were independently evaluated by three pathologists to assess the expression of specific markers in tumor-infiltrating lymphocytes within both the tumor core and the tumor margin. The tumor margin was defined as a region extending 200 μm inward from the histologically identifiable invasive edge of HCC tissues. Protein expression was scored by multiplying the percentage of positive cells (0–4: 0% = 0; 1%–25% = 1; 26%–50% = 2; 51%–75% = 3; > 75% = 4) by staining intensity (0 = none; 1 = weak; 2 = moderate; 3 = strong). A total score ≤5 was classified as low expression, while >5 indicated high expression. Discrepancies were resolved by joint review.

### Enzyme-linked immunosorbent assay (ELISA)

A total of 5 × 10⁵ HCC cells or NK cells were cultured and subjected to the indicated treatments for 48 hours. After incubation, cell culture supernatants were collected and concentrated using Amicon Ultra centrifugal filters by centrifugation at 4,000 × g for 30 min at 4 °C. The concentrations of MBL2, GZMB, and perforin in the supernatants were quantified using ELISA kits from Cloud-Clone Corporation, following the manufacturer’s protocols. Briefly, samples and standards were added to 96-well plates pre-coated with specific capture antibodies and incubated. After washing to remove unbound substances, biotin-labeled detection antibodies were applied, followed by HRP-conjugated streptavidin. Substrate solution was added for color development, and the reaction was terminated with a stop solution. The optical density (OD) was measured at 450 nm. Sample concentrations were calculated by interpolation from a standard curve generated with known concentrations of the respective proteins.

### Immunoprecipitation (IP), co-IP and RNA-IP (RIP)

Cells were lysed in RIPA buffer supplemented with protease inhibitors (1:100; Epizyme). Equal amounts of total cell lysates (500 μg of protein) were incubated with the indicated antibodies and Protein A/G agarose beads (sc-2003; Santa Cruz) overnight at 4 °C. Following incubation, the beads were washed three times with lysis buffer to remove non-specifically bound proteins.

Co-IP was conducted to evaluate the interaction between MBL2 and integrin β1. Protein complexes were resolved on a 10% SDS-PAGE gel and transferred onto PVDF membranes. Membranes were blocked with 5% nonfat milk in Tris-buffered saline and then incubated with primary antibodies, followed by HRP-conjugated secondary antibodies. Protein signals were detected using an ECL chemiluminescence reagent (FD8030; FDbio) and visualized with a chemiluminescence detection system (Bio-Rad).

To investigate the binding of HBx protein to *MBL2* mRNA, RIP was performed using the EZ-Magna RIP Kit (#17-10086; Merck) in conjunction with qPCR. Briefly, 293T cells were transfected with the HBx plasmid for 48 hours, lysed in RIP lysis buffer, and incubated with magnetic beads conjugated with anti-HBx or control IgG antibodies. After three washes with the wash buffer, RNA bound to the magnetic beads was eluted and used as a template for qPCR analysis. The relative abundance of *MBL2* mRNA in the eluates was quantified to assess the HBx-*MBL2* mRNA interaction.

### Statistical analysis

Statistical analyses were performed using SPSS 22.0 (IBM). Data are presented as mean ± SD unless otherwise specified. Normality and variance homogeneity were evaluated using Shapiro–Wilk and Levene’s tests, respectively. Student *t* test or one-way ANOVA followed by Tukey’s post-hoc test was applied for group comparisons in qPCR, flow cytometry, IHC, CCK-8, colony formation, Transwell analyses, LDH assays and ELISA assays. For all in vivo animal experiments, nonparametric counterparts were utilized, specifically the Mann–Whitney *U* test for two-group comparisons and the Kruskal–Wallis test followed by Dunn’s multiple comparisons test for multi-group analyses. Kaplan–Meier curves with the log-rank test were used for survival analysis. Statistical significance was set to *p* < 0.05, whereas “ns” indicated no significance.

## Supporting information

S1 Fig (A) The Human Protein Atlas database showed that the expression of MBL2 was highest in Huh7 cells, whereas the expression in Hep3B cells was relatively low. (B) qPCR was used to confirm the stable construction of MBL2-knockdown cell lines (Huh7-MBL2-shRNA1&2) and the establishment of full-length MBL2 and MBL2^ΔSP^ overexpression cell lines. CCK-8 assays were performed to evaluate the proliferative effects of recombinant MBL2 at three concentrations (0.25 ng/mL, 0.50 ng/mL, and 0.75 ng/mL) on Hep3B (C) and LM3 cells (D), with the PBS-treated group serving as a control. All values are shown as mean ± SD. ***p < 0.001. ns indicates no significance. Source data are available in S1 Data. rMBL2, recombinant mannose-binding lectin 2; MBL2^ΔSP^, MBL2 proteins lacking the signal peptide.
(TIF)

S2 Fig (A) Flow cytometry revealed that the proportion of CD3^+^ CD8^+^ T cells within the CD3^+^ T cell population was not directly altered by rMBL2 treatment. (B) A schematic representation illustrating the flow cytometry gating strategy and corresponding FMO controls (NKG2D, NKp46, PD-L1, GZMB, and IFN-γ) for peripheral CD3^−^ CD56^+^ NK cells. (C) rMBL2 treatment significantly elevated NKG2D expression on peripheral NK cells, as assessed by flow cytometry. (D) Flow cytometry scatter plots demonstrated that rMBL2 treatment enhanced NKp46 expression on peripheral NK cells. All values are shown as mean ± SD. ***p < 0.001. Source data are available in S1 Data. PBMCs, peripheral blood mononuclear cells.
(TIF)

S3 Fig (A and B) Sequential gating workflow and CD107a FMO control for CD107a degranulation assay following (A) stimulation with rMBL2 or (B) co-culture with Hep3B-MBL2 cells. (C) CD107a expression was significantly increased in rMBL2-treated NK cells compared to the PBS control. (D) Co-culture with Hep3B cells overexpressing MBL2 also upregulated CD107a expression on NK92 cells. (E) Flow cytometry scatter plots illustrating GZMB production in the rMBL2-treated peripheral NK cells. (F) Representative scatter plots showing CD3^−^ CD56^bright^ and CD3^−^ CD56^dim^ NK cell subsets in PBMCs treated with PBS or rMBL2 (250 ng/mL) for 48 hour. (G and H) Flow cytometry assays revealed that rMBL2 stimulation significantly increased NKG2D expression on both CD3^−^ CD56^bright^ and CD3^−^ CD56^dim^ NK cell subsets compared to the PBS control group. (I) Schematic diagram illustrating the workflow of flow cytometry for the detection of murine NK cell membrane receptors and intracellular proteins. (J) Schematic illustration of the co-culture model involving Hepa1–6 cells and murine liver NK cells. (K) Schematic representation of the co-culture model of NK92 cells and HCC cells. (L) Transwell migration assays were performed to evaluate the effect of MBL2 on NK cell recruitment. (M) LDH assays demonstrated increased LDH levels in the Hep3B-MBL2 groups after co-culture with NK92 cells. All values are shown as mean ± SD. **p < 0.01, ***p < 0.001. ns indicates no significance. Source data are available in S1 Data. ‌FMO, Fluorescence Minus One; LDH, lactate dehydrogenase.
(TIF)

S4 Fig (A, B) The Gene-concept Networks represented the membrane proteins that interact with MBL2 as identified through mass spectrometry analysis. (C) The heatmaps represented the correlation between integrin β1 and canonical pathways derived from Reactome gene sets within the transcriptional profile of infiltrating NK cells in HCC. (D) Flow cytometry indicated that MBL2 enhanced the proportion of the NKp46⁺ NK cells and the GZMB⁺ NK cells, an effect that was reversed upon RGD peptide treatment. Source data are available in S1 Data. RGD, Arg-Gly-Asp peptide.
(TIF)

S5 Fig (A) The heatmap revealed differential genes in eukaryotic transcriptome sequencing to investigate the molecular mechanisms underlying MBL2 activation in NK cells. (B) The GSE183349 dataset was used to perform correlation analysis between integrin β1 and canonical pathways within infiltrating NK cells in HCC (R = 0.62). (C) qPCR demonstrated that MBL2 downregulated the expression of IFNGR2 in HCC transcriptionally. (D) Flow cytometry revealed that MK2206 could reverse MBL2-induced upregulation of NKG2D^+^ NK and NKp46^+^ NK cells. (E) Sequential gating strategy and corresponding FMO controls (NKp46, NKG2D, GZMB, and IFN-γ) in AKT pathway rescue experiments. Representative flow cytometry scatter plots showing a significant reduction in PD-1⁺ CTLs (F) and TIM-3⁺ CTLs (G) in the IL-13 and IL-25 treatment groups compared with the PBS control group. Neutralization of IL-13 and IL-25 reversed the suppression of PD-1⁺ CTL (H) and TIM-3⁺ CTL (I) populations mediated by MBL2-activated NK cells. All values are shown as mean ± SD. ***p < 0.001. Source data are available in S1 Data. MK2206, MK-2206 2HCl, a pan-AKT inhibitor.
(TIF)

S6 Fig (A) IHC staining revealed a marked reduction in PD-1 expression in the Luc-MBL2 + anti -PD-L1 group compared with the Luc-NC + anti–PD-L1 group. (B–D) Kaplan-Meier survival analysis revealed discernible differences in (B) progression-free survival (PFS), (C) disease-specific survival (DSS) and (D) recurrence-free survival (RFS) associated with high MBL2 expression.
(TIF)

S1 Data Source data for all figures. An Excel spreadsheet containing the underlying numerical data for Figs 1B–1D, 1F–1I, 1K, 1L, 2A–2K, 2M, 2N, 3F–3H, 3K–3M, 4A–4G, 4J–4O, 5A–5C, 5E–5I, 5M, 5O–5Q, 6A, 6B, 6D–6F, 6H, 7A, 7C–7F, S1A–S1C, S2A, S2C, S3C, S3D, S3G, S3H, S3L, S3M, and S5C.
(XLSX)

S2 Data Supplementary Methods. (DOCX)

S3 Data Supplementary Materials. (A) Primary antibodies in western blot assays; (B) Oligonucleotide information; (C) Fluorochrome-conjugated antibodies in flow cytometry.
(DOCX)

S4 Data Western blot quantification and statistical analyses. (PDF)

S1 Raw Images Raw images of all blots and gels. (PDF)

## Abbreviations

anti-ASGM1: anti-asialo GM1
CM: culture medium
CTLs: cytotoxic T lymphocytes
DCN: decorin
DSS: disease-specific survival
ED: extracellular domain
FMO: fluorescence minus one
GSEA: gene set enrichment analysis
GZMB: granzyme B
HBV: hepatitis B virus
HBx: Hepatitis B virus X protein
HCC: hepatocellular carcinoma
IFN: interferons
LDH: lactate dehydrogenase
Lr-NK: liver-resident NK
MBL2: mannose-binding lectin 2
MHC-I: major histocompatibility complex class I
NK: natural killer
OPG: osteoprotegerin
OS: overall survival
p-AKT: phosphorylated AKT
PBMCs: peripheral blood mononuclear cells
PFS: progression-free survival
PVDF: Polyvinylidene fluoride membranes
rMBL2: recombinant MBL2
RFS: recurrence-free survival
RGD: Arg-Gly-Asp peptide
RT: room temperature
SP: signal peptide
TME: tumor micro environment
WB: western blot
