# Supplementary figures and images for: Mannose-binding lectin 2 secreted by hepatocellular carcinoma cells recruits and activates natural killer cells to reshape an immune-activated microenvironment
Source: PLoS Biol. 2026 May 20;24(5):e3003793. doi: 10.1371/journal.pbio.3003793 (PMC13189296; doi:10.1371/journal.pbio.3003793)

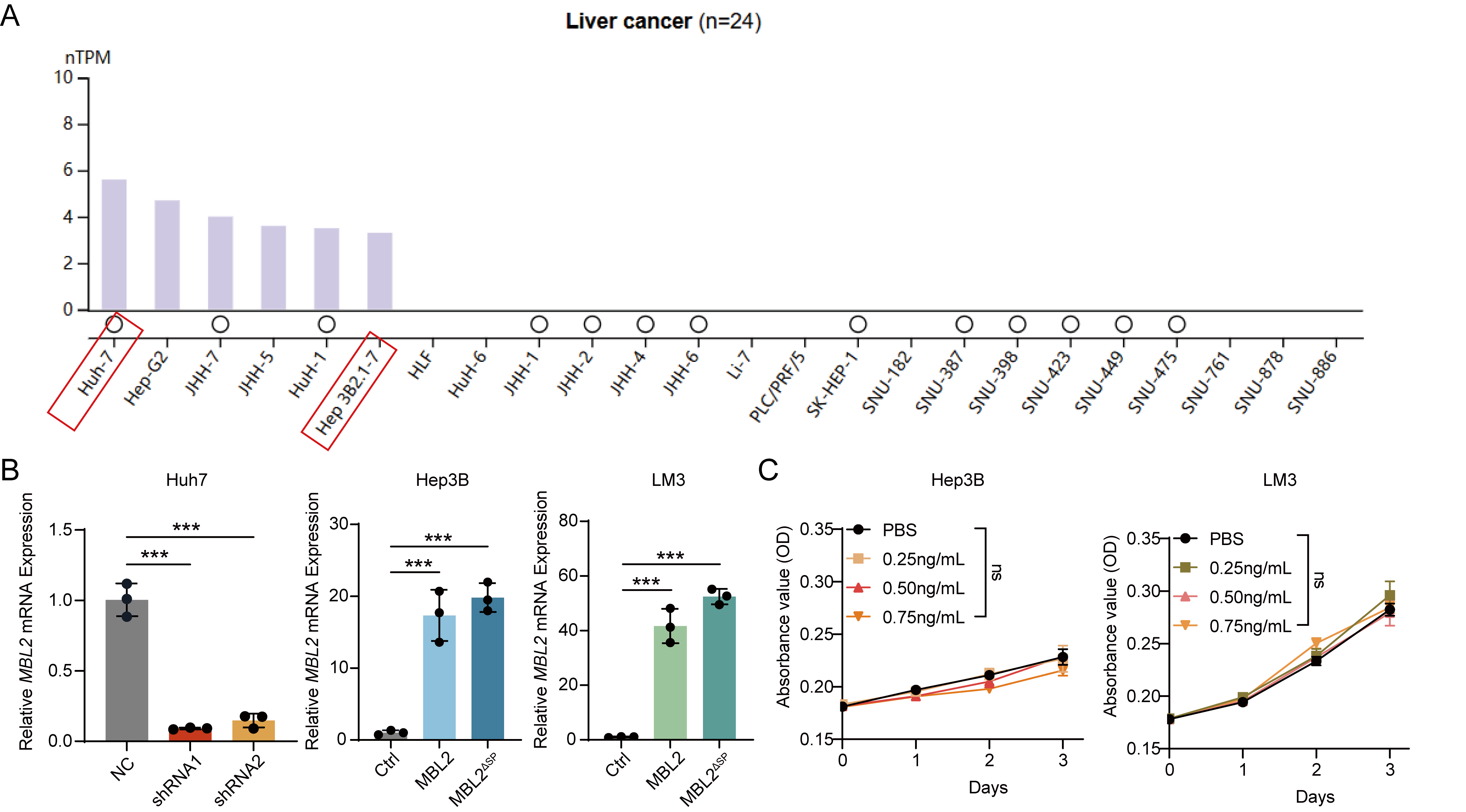

Supplement: S1 Fig — (B) qPCR was used to confirm the stable construction of MBL2-knockdown cell lines (Huh7-MBL2-shRNA1&2) and the establishment of full-length MBL2 and MBL2ΔSP overexpression cell lines. CCK-8 assays were performed to evaluate the proliferative effects of recombinant MBL2 at three concentrations (0.25 ng/mL, 0.50 ng/mL, and 0.75 ng/mL) on Hep3B (C) and LM3 cells (D), with the PBS-treated group serving as a control. All values are shown as mean ± SD. ***p < 0.001. ns indicates no significance. Source data are available in S1 Data. rMBL2, recombinant mannose-binding lectin 2; MBL2ΔSP, MBL2 proteins lacking the signal peptide. (TIF) [file pbio.3003793.s001.tif]

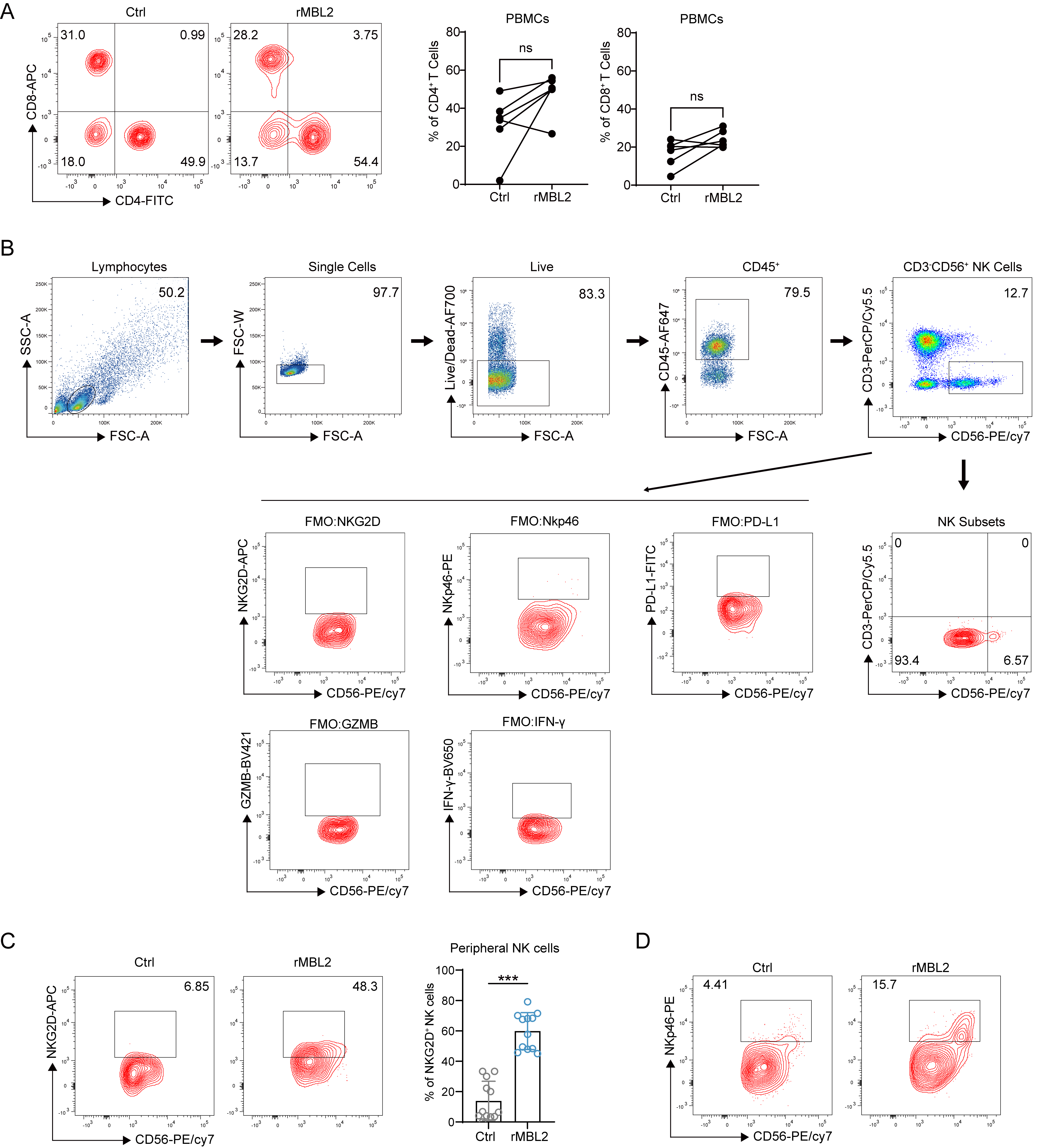

Supplement: S2 Fig — (B) A schematic representation illustrating the flow cytometry gating strategy and corresponding FMO controls (NKG2D, NKp46, PD-L1, GZMB, and IFN-γ) for peripheral CD3− CD56+ NK cells. (C) rMBL2 treatment significantly elevated NKG2D expression on peripheral NK cells, as assessed by flow cytometry. (D) Flow cytometry scatter plots demonstrated that rMBL2 treatment enhanced NKp46 expression on peripheral NK cells. All values are shown as mean ± SD. ***p < 0.001. Source data are available in S1 Data. PBMCs, peripheral blood mononuclear cells. (TIF) [file pbio.3003793.s002.tif]

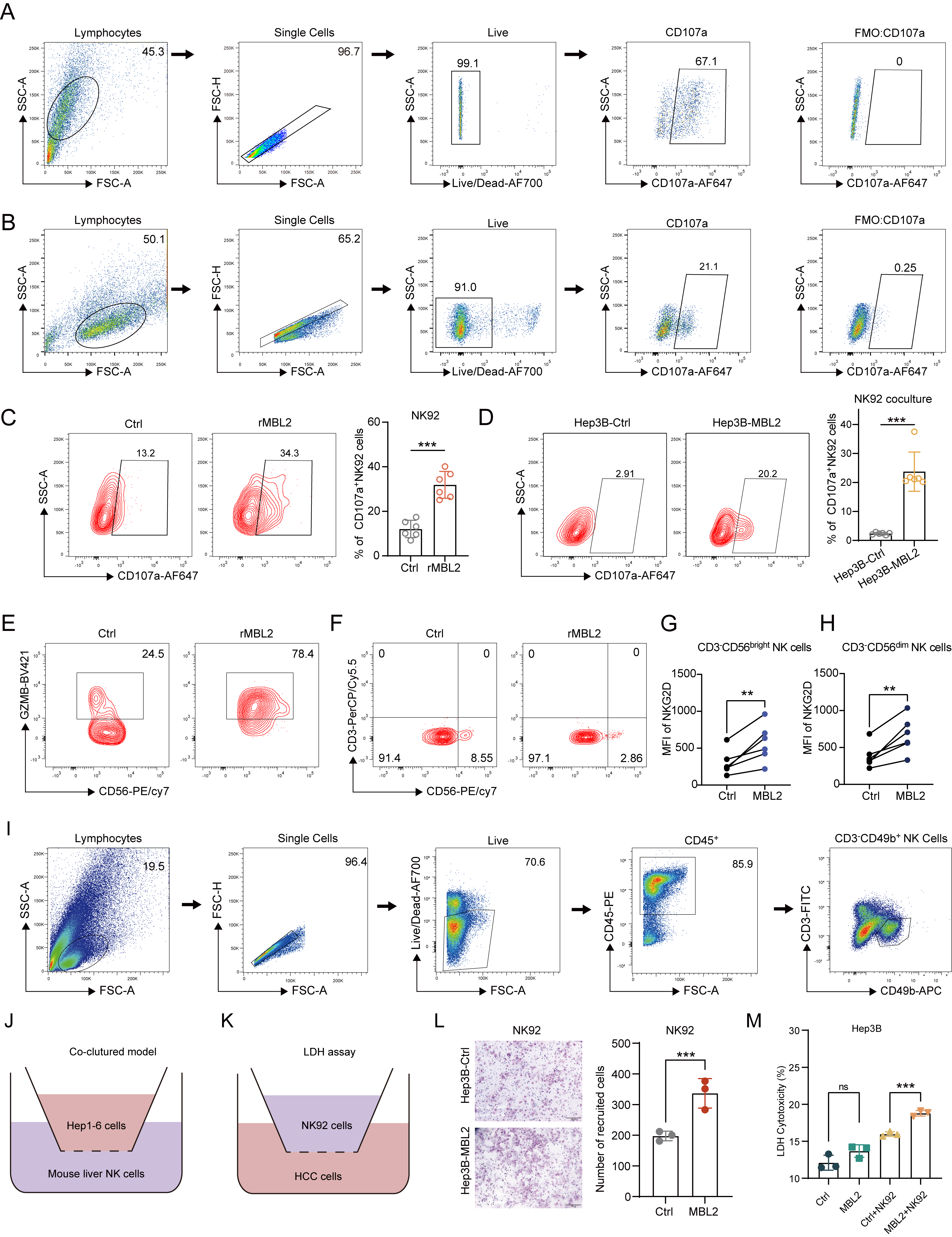

Supplement: S3 Fig — (C) CD107a expression was significantly increased in rMBL2-treated NK cells compared to the PBS control. (D) Co-culture with Hep3B cells overexpressing MBL2 also upregulated CD107a expression on NK92 cells. (E) Flow cytometry scatter plots illustrating GZMB production in the rMBL2-treated peripheral NK cells. (F) Representative scatter plots showing CD3− CD56bright and CD3− CD56dim NK cell subsets in PBMCs treated with PBS or rMBL2 (250 ng/mL) for 48 hour. (G and H) Flow cytometry assays revealed that rMBL2 stimulation significantly increased NKG2D expression on both CD3− CD56bright and CD3− CD56dim NK cell subsets compared to the PBS control group. (I) Schematic diagram illustrating the workflow of flow cytometry for the detection of murine NK cell membrane receptors and intracellular proteins. (J) Schematic illustration of the co-culture model involving Hepa1–6 cells and murine liver NK cells. (K) Schematic representation of the co-culture model of NK92 cells and HCC cells. (L) Transwell migration assays were performed to evaluate the effect of MBL2 on NK cell recruitment. (M) LDH assays demonstrated increased LDH levels in the Hep3B-MBL2 groups after co-culture with NK92 cells. All values are shown as mean ± SD. **p < 0.01, ***p < 0.001. ns indicates no significance. Source data are available in S1 Data. ‌FMO, Fluorescence Minus One; LDH, lactate dehydrogenase. (TIF) [file pbio.3003793.s003.tif]

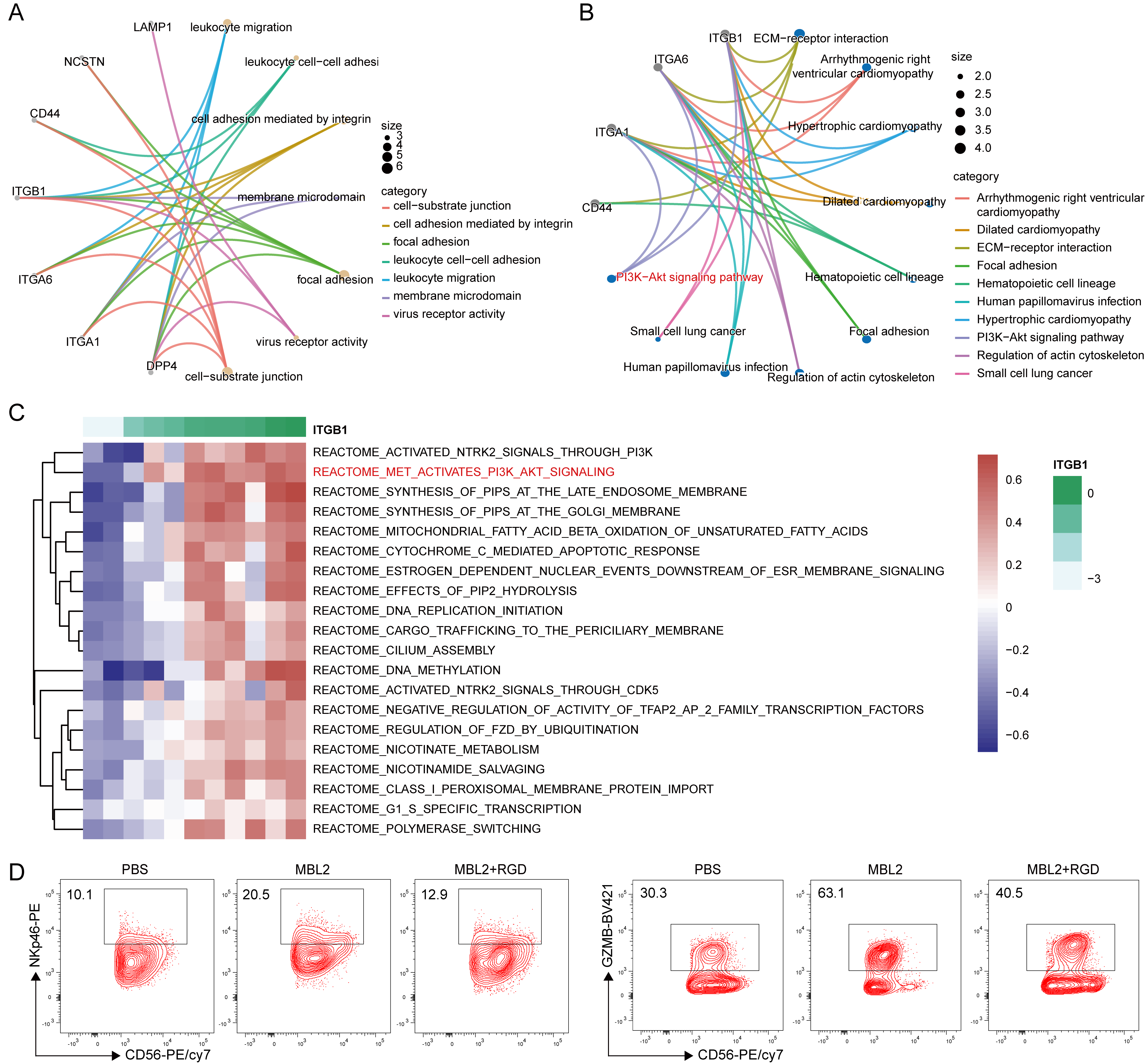

Supplement: S4 Fig — (C) The heatmaps represented the correlation between integrin β1 and canonical pathways derived from Reactome gene sets within the transcriptional profile of infiltrating NK cells in HCC. (D) Flow cytometry indicated that MBL2 enhanced the proportion of the NKp46⁺ NK cells and the GZMB⁺ NK cells, an effect that was reversed upon RGD peptide treatment. Source data are available in S1 Data. RGD, Arg-Gly-Asp peptide. (TIF) [file pbio.3003793.s004.tif]

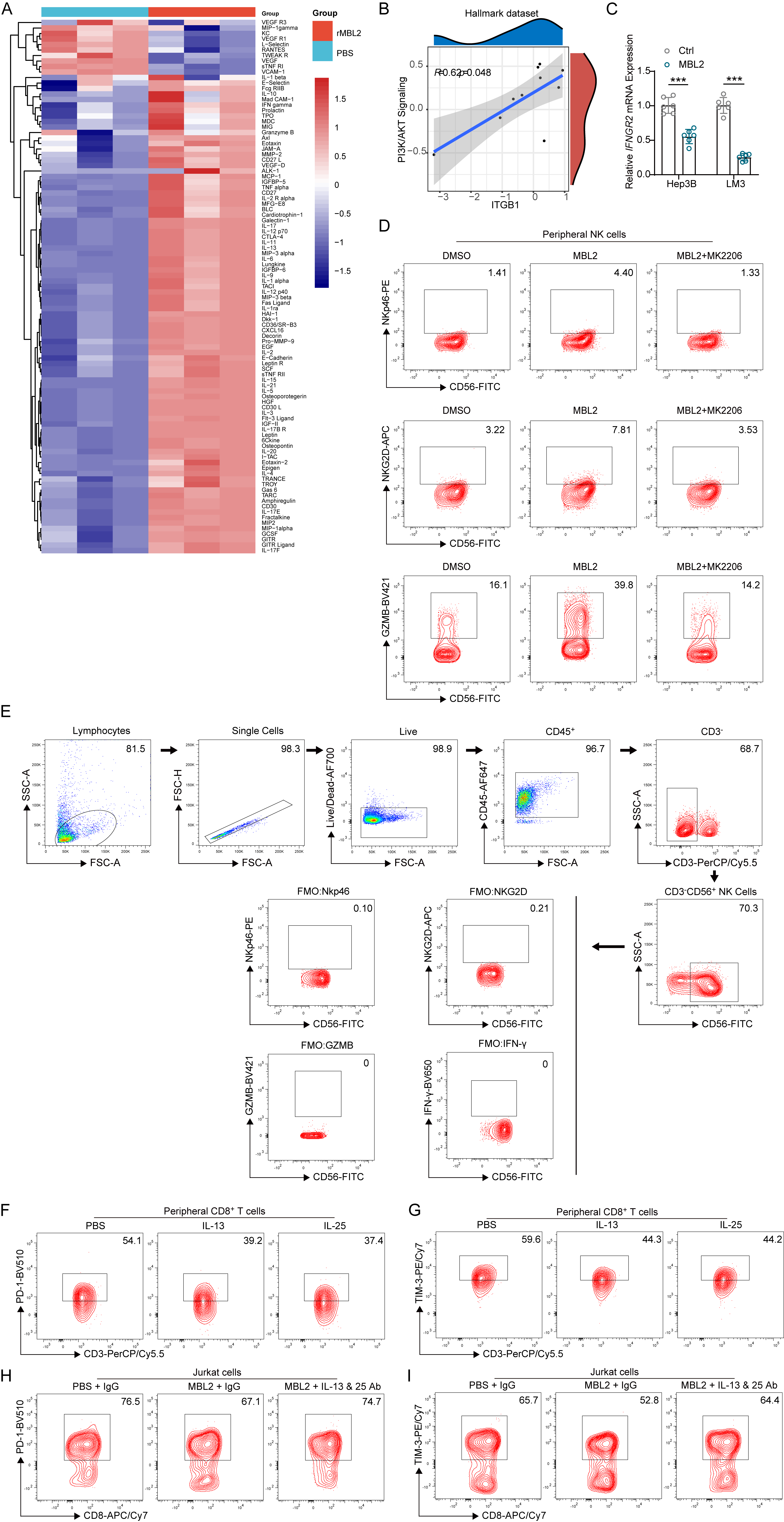

Supplement: S5 Fig — (B) The GSE183349 dataset was used to perform correlation analysis between integrin β1 and canonical pathways within infiltrating NK cells in HCC (R = 0.62). (C) qPCR demonstrated that MBL2 downregulated the expression of IFNGR2 in HCC transcriptionally. (D) Flow cytometry revealed that MK2206 could reverse MBL2-induced upregulation of NKG2D+ NK and NKp46+ NK cells. (E) Sequential gating strategy and corresponding FMO controls (NKp46, NKG2D, GZMB, and IFN-γ) in AKT pathway rescue experiments. Representative flow cytometry scatter plots showing a significant reduction in PD-1⁺ CTLs (F) and TIM-3⁺ CTLs (G) in the IL-13 and IL-25 treatment groups compared with the PBS control group. Neutralization of IL-13 and IL-25 reversed the suppression of PD-1⁺ CTL (H) and TIM-3⁺ CTL (I) populations mediated by MBL2-activated NK cells. All values are shown as mean ± SD. ***p < 0.001. Source data are available in S1 Data. MK2206, MK-2206 2HCl, a pan-AKT inhibitor. (TIF) [file pbio.3003793.s005.tif]

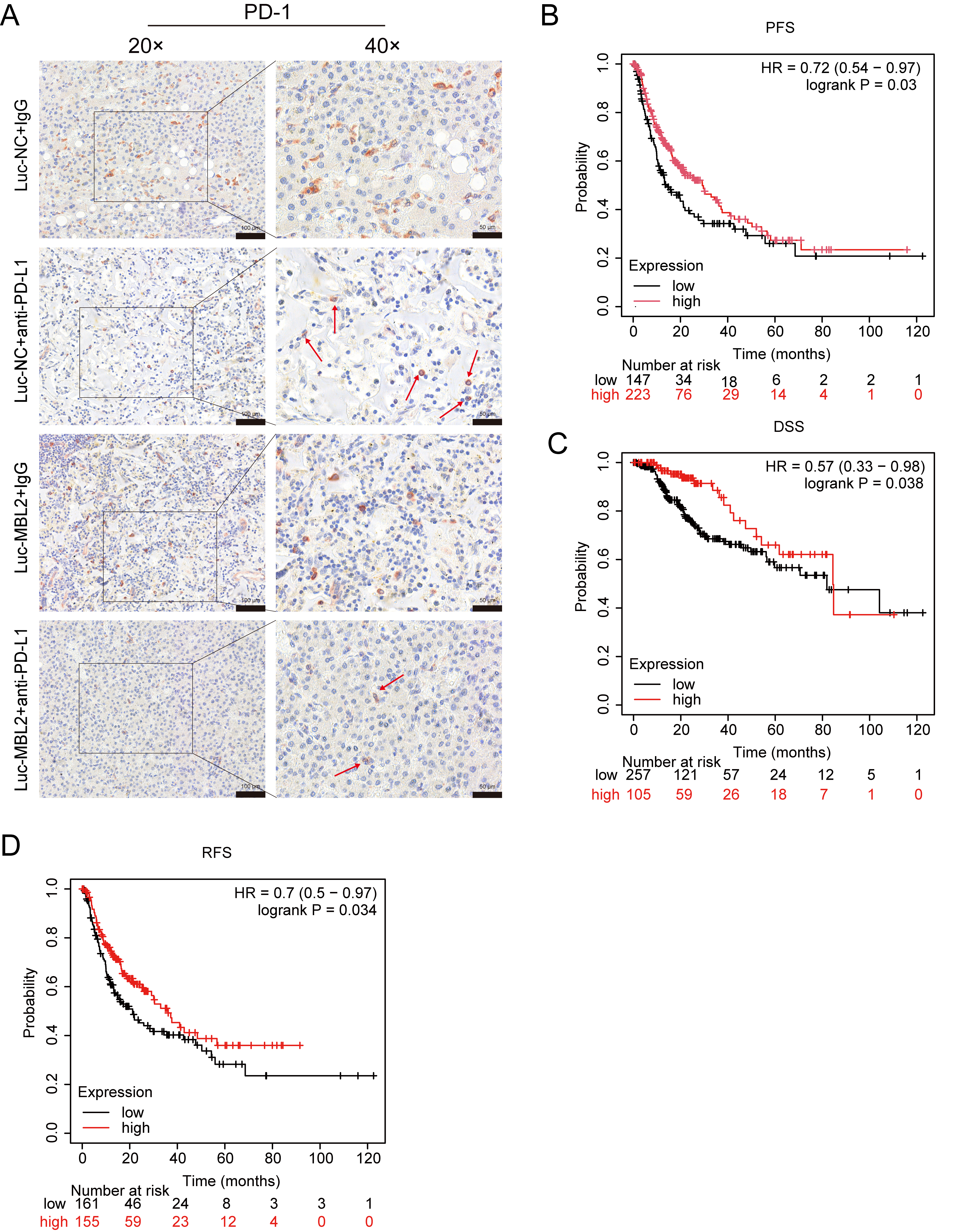

Supplement: S6 Fig — (B–D) Kaplan-Meier survival analysis revealed discernible differences in (B) progression-free survival (PFS), (C) disease-specific survival (DSS) and (D) recurrence-free survival (RFS) associated with high MBL2 expression. (TIF) [file pbio.3003793.s006.tif]
