# Supplementary material for: Mannose-binding lectin 2 secreted by hepatocellular carcinoma cells recruits and activates natural killer cells to reshape an immune-activated microenvironment
Source: PLoS Biol. 2026 May 20;24(5):e3003793. doi: 10.1371/journal.pbio.3003793 (PMC13189296; doi:10.1371/journal.pbio.3003793)
Supplement: S2 Data — (DOCX) [file pbio.3003793.s008.docx]

**Supplementary Materials and Methods:**

## Cell counting kit 8 (CCK-8) assays

Cells were seeded at a density of 1×103 cells/well in 96-well plates containing 100 μL of DMEM supplemented with 10% FBS. The plates were then incubated at 37°C under a 5% CO2 atmosphere for 6 h. Subsequently, 10 μL of CCK-8 solution was added to each well every 24 h. After a 2-h incubation with the CCK-8 solution, the absorbance of each sample was measured at 450 nm using a microplate reader (Varioskan LUX, Thermo Scientific, Waltham, MA, USA).

## Colony formation assay

One thousand viable HCC cells were plated in 6-well plates and incubated in DMEM for seven days to form colonies. The colonies were rinsed three times with PBS, then fixed with 4% formaldehyde for 15 min, and subsequently stained with Giemsa staining solution (Solarbio, Beijing, China) for 30 min. After washing three times with distilled water, images of the colonies on each plate were recorded using a light micro-scope. Colonies were analyzed using ImageJ software, and the results of the three ex-periments were averaged.

## Transwell migration assay

Cells (5×10^4^ cells/300 μL) suspended in serum-free medium were placed in the upper chamber of each 8-um-pore Transwell chamber (Corning Star, Cambridge, MA, USA). The lower chamber was filled with DMEM containing 20% FBS as a chem-oattractant. The cells were allowed to migrate through the porous membrane for 24 h at 37°C. Subsequently, five randomly chosen microscopic fields (×200 magnification) were observed, and the cells were counted. A minimum of four chambers from three independent experiments were analyzed.

## RNA isolation and real-time quantitative PCR

Total RNA was isolated using TRIzol reagent following the recommended protocol and previously published methods.^21^ Polyadenylated mRNA was purified from total RNA and used for reverse transcription. qPCR was performed using SYBR® Green PCR master mix (4309155; Thermo Fisher Scientific, Shanghai, China) on an ABI 7500HT system (Applied Biosystems, CA, USA). *GAPDH* was an endogenous control. All primers were synthesized by Tsingke Biotechnology Co., Ltd (Beijing, China; **S2B**). The expression level of each target gene was presented as a fold change relative to the control group and was calculated using the 2^-ΔΔCT^ method for relative quantification.

## Immunofluorescence

HCC tissue sections and cells were cultured in confocal dishes overnight, fixed with 4% paraformaldehyde for 30 min, and permeabilized with 0.25% Triton X-100 for 10 min. The slides were blocked with 10% fetal bovine serum at RT for 30 min, incubated with indicated antibodies overnight at 4 °C, and washed three times with PBS. The samples were then stained with Coralite488 or Coralite594 conjugated secondary antibodies (SA00013, 1:100; Proteintech) for 1 h at RT and stained with DAPI (Invitrogen, Thermo Fisher Scientific). Cells were visualized using a FluoView FV1000 confocal microscope (Olympus). The acquired images were analyzed using the Olympus FLUOVIEW version 4.2a Viewer.

## GST pull-down assays

Bacteria-expressed GST or GST-tagged ITGB1-ED, ITGB1-ED1, and ITGB1-ED2 were harvested using GST-tag Beaver Beads magnetic beads according to the manufacturer’s instructions (Beaver, 70601-K10). Purified proteins were then added to the lysates of 293T cells transfected with MBL2Flag overnight. The bound proteins were eluted and analyzed using WB.
