# Supplementary material for: Mannose-binding lectin 2 secreted by hepatocellular carcinoma cells recruits and activates natural killer cells to reshape an immune-activated microenvironment
Source: PLoS Biol. 2026 May 20;24(5):e3003793. doi: 10.1371/journal.pbio.3003793 (PMC13189296; doi:10.1371/journal.pbio.3003793)
Supplement: S3 Data — (A) Primary antibodies in western blot assays; (B) Oligonucleotide information; (C) Fluorochrome-conjugated antibodies in flow cytometry. (DOCX) [file pbio.3003793.s009.docx]

**Supplementary Tables**

S2A: Primary antibodies in Western blot.

| Antibodies | Source and identifier |
| --- | --- |
| anti-β-tubulin | 1:1000, TA-10; ZsBio, Beijing, China |
| anti-β-actin | 1:1000, TA-09; ZsBio |
| anti-MBL2 | 1:1000, GTX132722; Genetex, Irvine, CA, USA |
| anti-ITGB1 | 1:1000, GTX128839; Genetex |
| anti-GST | 1:1000, #2625; Cell Signaling Technology(CST), Massachusetts, USA |
| anti-FLAG | 1:1000, F1804; Sigma-Aldrich, Darmstadt, Germany |
| anti-FAK | 1:1000, #3285; CST |
| anti-p-FAK | 1:1000, #8556; CST |
| anti-PI3K | 1:1000, #4249; CST |
| anti-p-PI3K | 1:1000, 60225-1-Ig; Proteintech, Chicago, USA |
| anti-AKT | 1:1000, 60203-2-Ig; Proteintech |
| anti-p-AKT | 1:1000, 66444-1-Ig; Proteintech |
| anti-IFNGR1 | 1:500, 10808-1-AP; Proteintech |
| anti-PD-L1 | 1:1000, 28076-1-AP; Proteintech |
| anti-HBX | 1:200, sc-57760; Santa Cruz, CA, USA |

S2B: Oligonucleotide information

| Primers | Species | Forword primer (5’-3’) | Reverse primer (5’-3’) |
| --- | --- | --- | --- |
| GAPDH | Human | CTCCTCCTGTTCGACAGTCA | TGCAGGAGGCATTGCTGATG |
| MBL2 | Human | GTATGGTGGCAGCGTCTTACTCAG | GAAGCCGTTGATGCCTGGAGAG |
| HBX | Human | CCGTCTGTGCCTTCTCATCTGC | AAGGTCGGTCGTTGACATTGCTG |
| IFNgR1 | Human | AGCAGGAAGTCGATTATGATCCC | CTGGCACTGAATCTCGTCACA |
| IFNgR1 | Human | GCAGGCTTCCCAATGGATTTC | CCCGACAGTCACATTCCGATA |
| IL13RA1 | Human | TGAGTGTCTCTGTTGAAAACCTC | GGGGTACTTCTATTGAACGACGA |
| IL17RB | Human | GGCTGCCTAGACCACATAATG | GCTGTGTTGGATAAGAGCCAT |
| PD-L1 | Human | TGGCATTTGCTGAACGCATTT | TGCAGCCAGGTCTAATTGTTTT |
| GAPDH | Mouse | CATCACTGCCACCCAGAAGACTG | ATGCCAGTGAGCTTCCCGTTCAG |
| MBL2 | Mouse | TGACAGTGGTTTATGCAGAGAC | CGTCACGTCCATCTTTGCC |
| PD-L1 | Mouse | GCTCCAAAGGACTTGTACGTG | TGATCTGAAGGGCAGCATTTC |
| Target | | genes Sequences | |
| hs-ITGB1-siRNA | | GCGAGUGUGAUAAUUUCAATT UUGAAAUUAUCACACUCGCTT | |

Table S3: Fluorochrome-conjugated antibodies in flow cytometry.

| Antibody | Catlog |
| --- | --- |
| Human/ Mouse-live/dead-FSV700 | 564997, 1:500, BD Biosciences |
| Human-CD45-Alexa Fluor647 | 304018, 1:200, Biolegend |
| Human-CD3-PerCP/Cy5.5 | 317336, 1:200, Biolegend |
| Human-CD56-PE/cy7 | A51078, 1:200, Beckman Coulter |
| Human-CD56-FITC | 318304, 1:100, Biolegend |
| Human-CD16-PE/Dazzle540 | 302053, 1:100, Biolegend |
| Human-NKG2D-APC | 320808, 1:200, Biolegend |
| Human-Nkp46-PE | 331908, 1:200, Biolegend |
| Human/ Mouse -GZMB-BV421 | 396414, 1:200, Biolegend |
| Human-IFN-γ-BV650 | 502538, 1:200, Biolegend |
| Human-CD4-FITC | 300505, 1:200, Biolegend |
| Human-CD8-APC | 344721, 1:200, Biolegend |
| Human-TIM-3-PE/Cy7 | 345014, 1:200, Biolegend |
| Human-PD-1-BV510 | 367424, 1:200, Biolegend |
| Human-PD-L1-FITC | 374509, 1:200, Biolegend |
| Human- CD107a-CoraLite Plus 647 | CL647-65051, 1:200, Proteintech |
| Mouse-CD45-PE | 103105, 1:100, Biolegend |
| Mouse-CD3-FITC | 100204, 1:100, Biolegend |
| Mouse-CD49b-APC | 103515, 1:100, Biolegend |
| Mouse-CD11b-PerCP/Cy5.5 | 101227, 1:100, Biolegend |
| Mouse-CD27-APC/Cy7 | 124225, 1:100, Biolegend |
| Mouse-NKG2D- PE/DazzleTM540 | 130214, 1:100, Biolegend |
| Mouse-Nkp46-BV650 | 137635, 1:100, Biolegend |
| Mouse-IFN-γ-PE/Cy7 | 505825, 1:100, Biolegend |
