# Supplementary material for: Mannose-binding lectin 2 secreted by hepatocellular carcinoma cells recruits and activates natural killer cells to reshape an immune-activated microenvironment
Source: PLoS Biol. 2026 May 20;24(5):e3003793. doi: 10.1371/journal.pbio.3003793 (PMC13189296; doi:10.1371/journal.pbio.3003793)

Figure 1-B

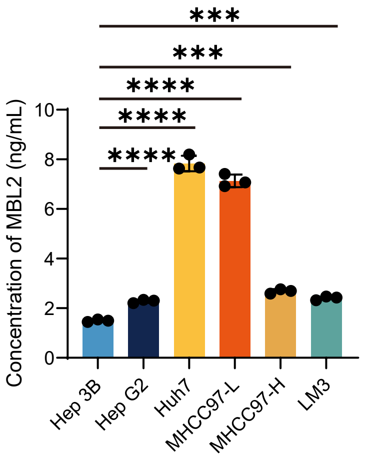

Figure 1-C

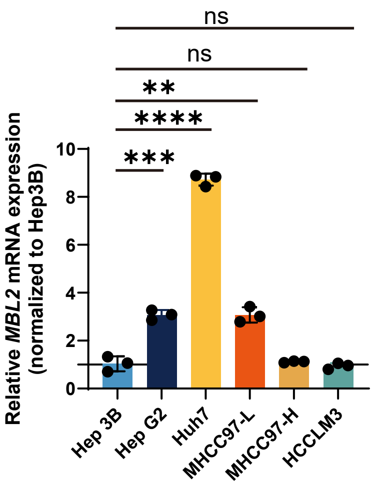

Figure 1-D

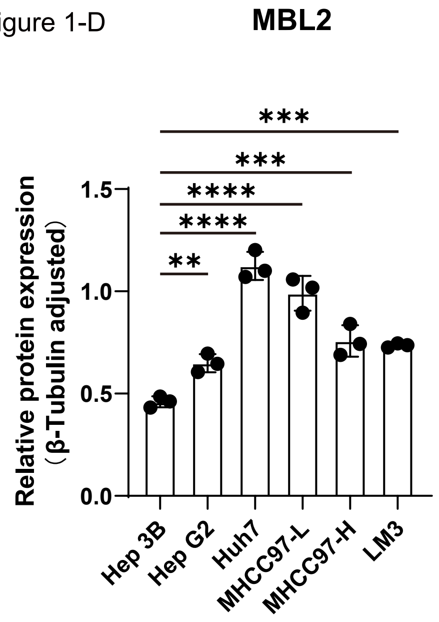

Figure 1-F

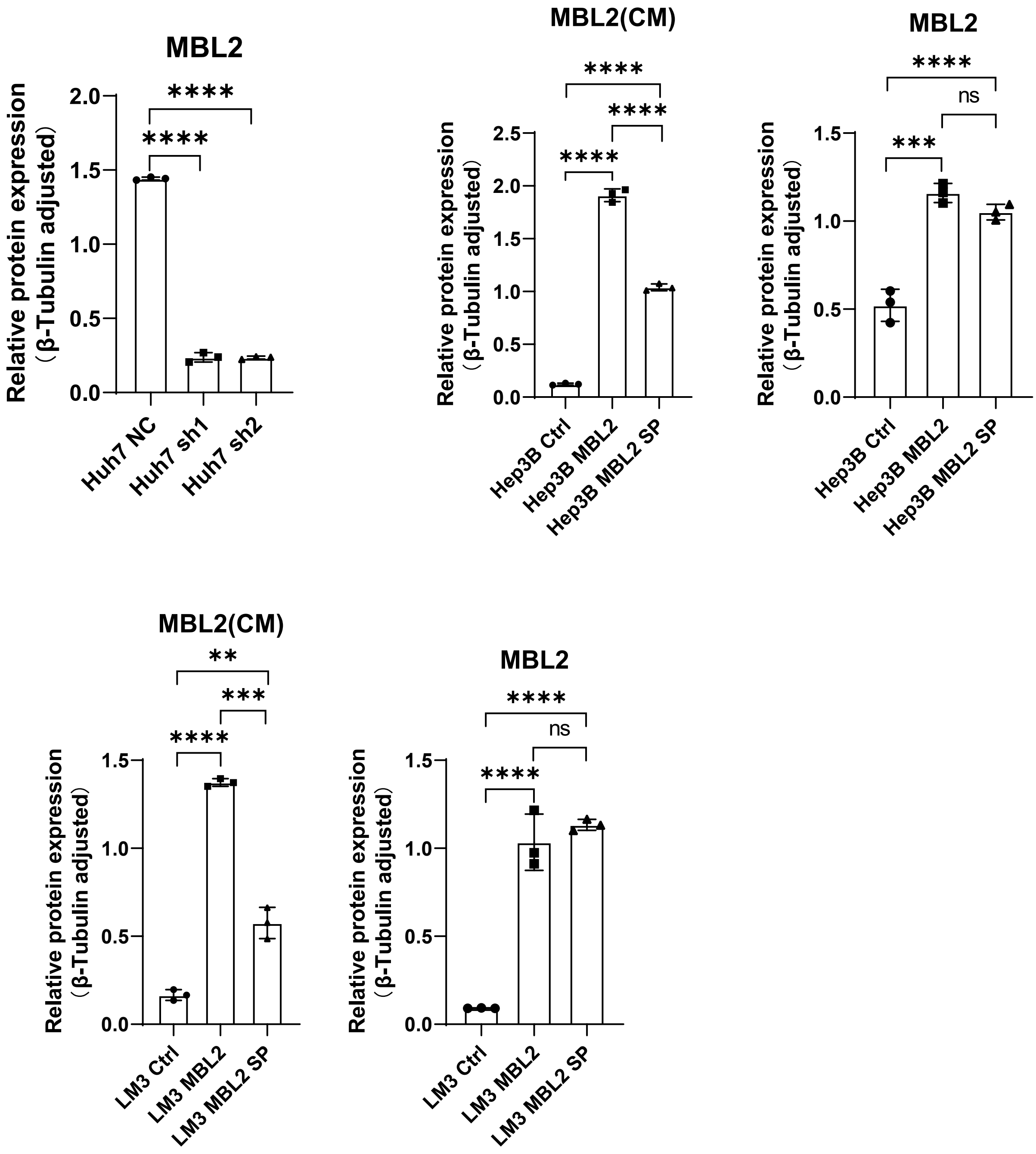

Figure 3-K

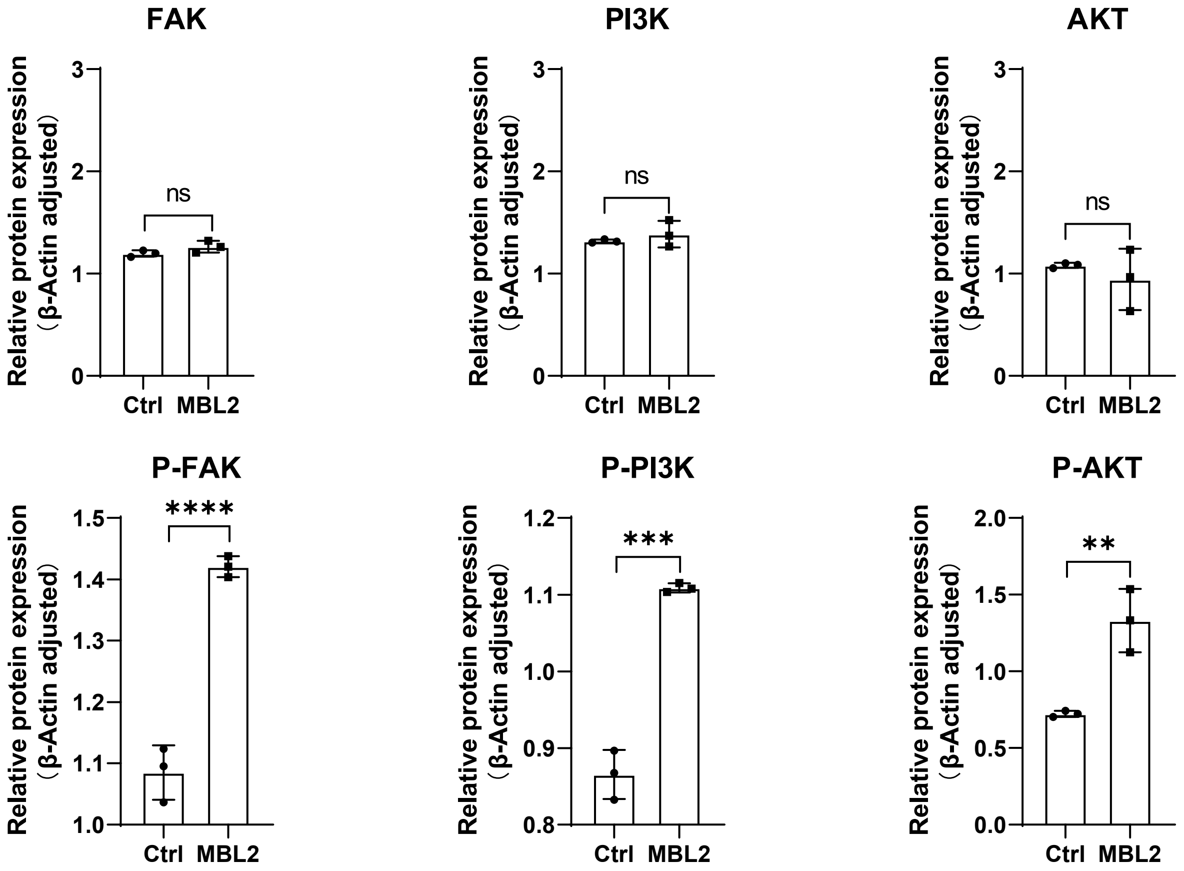

Figure 3-M

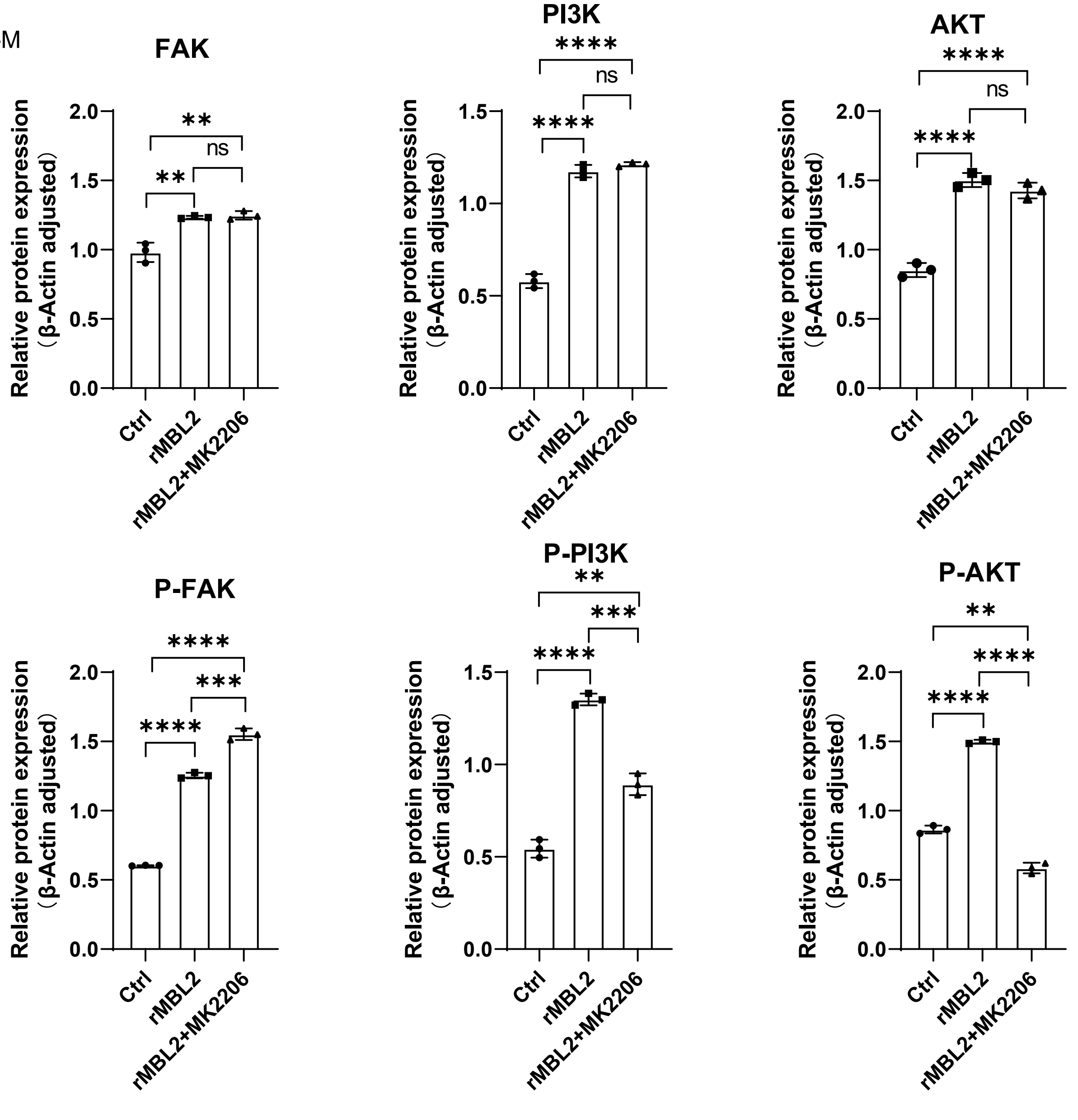

Figure 4-G

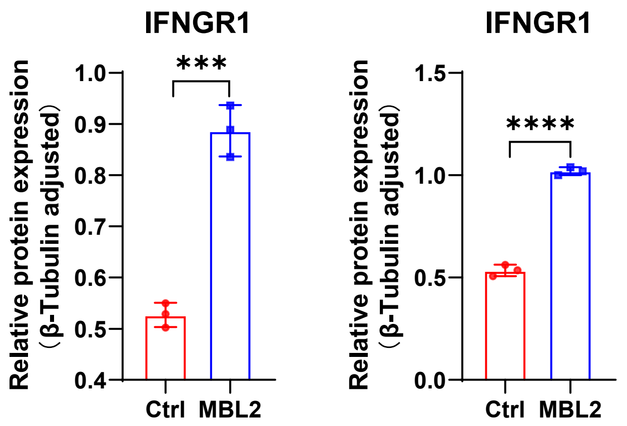

Figure 5-E

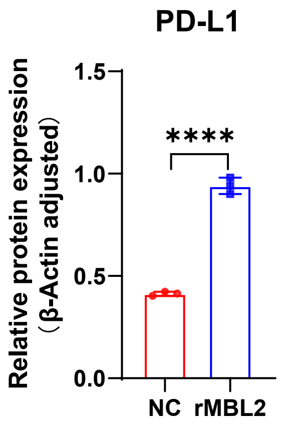

Figure 5-F

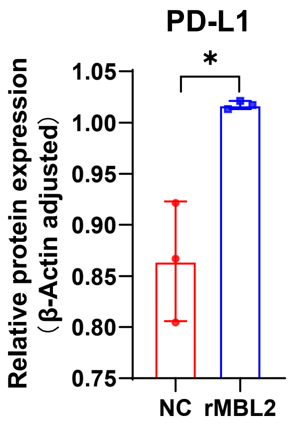

Figure 5-I

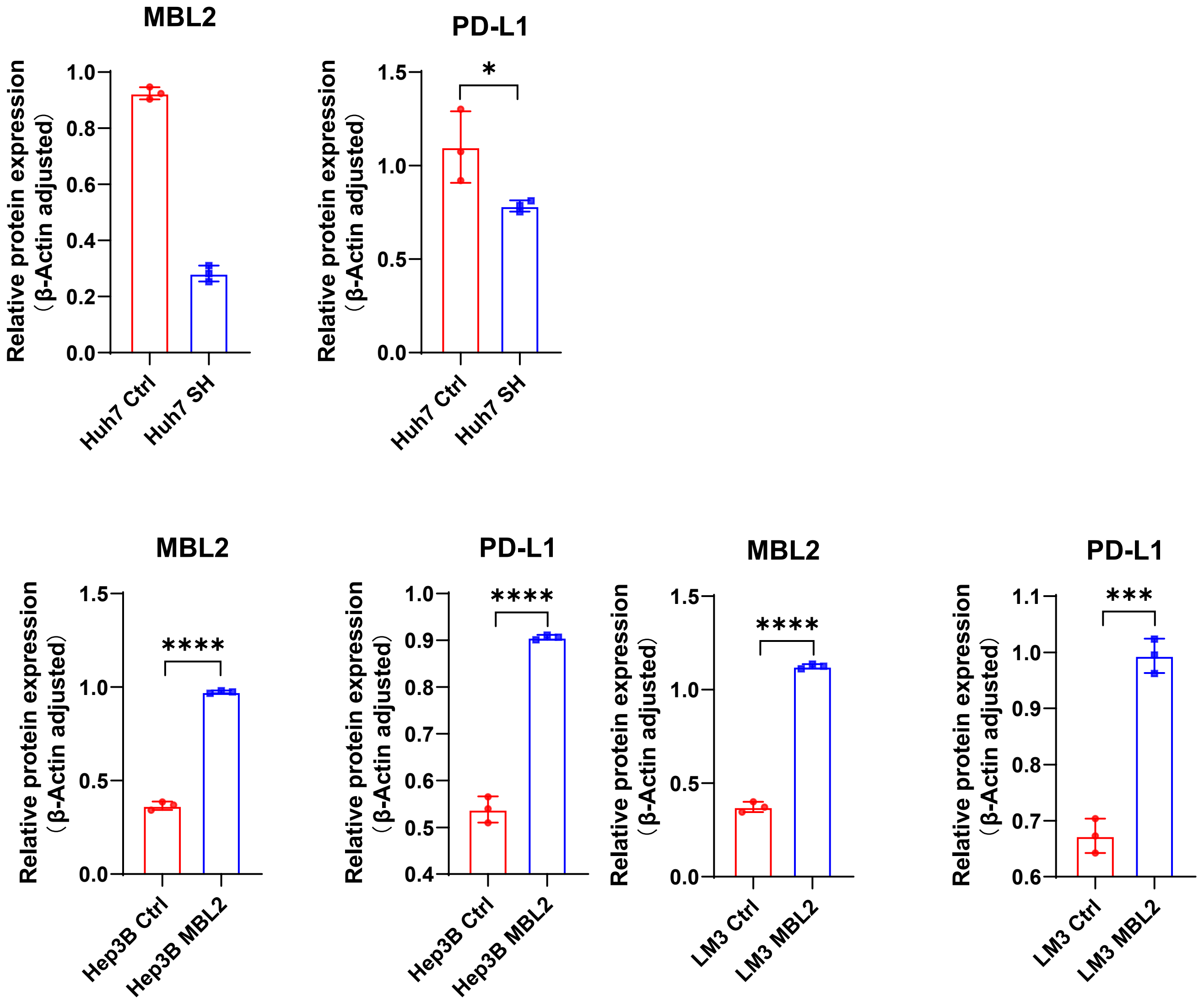

Figure 5-D

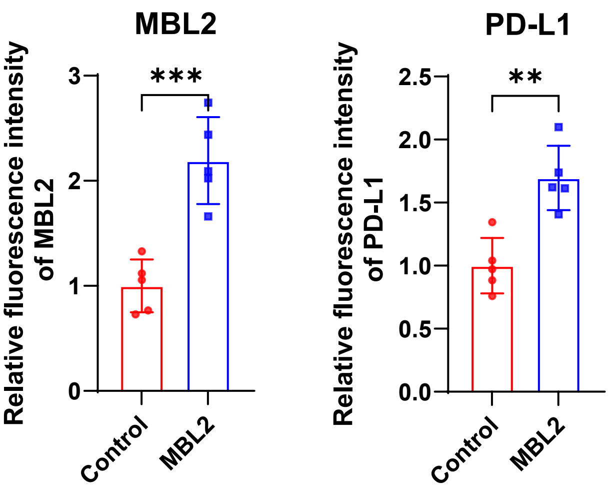

Figure 5-K

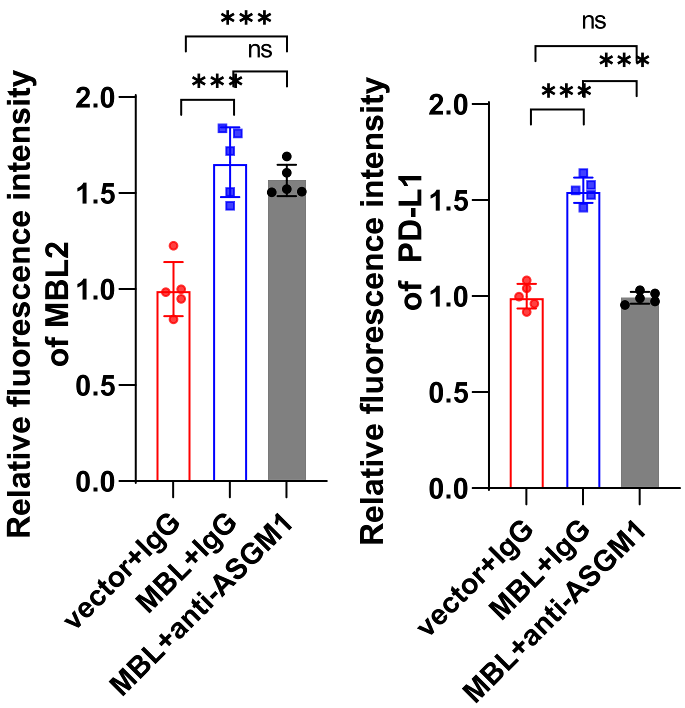

Figure 6-B

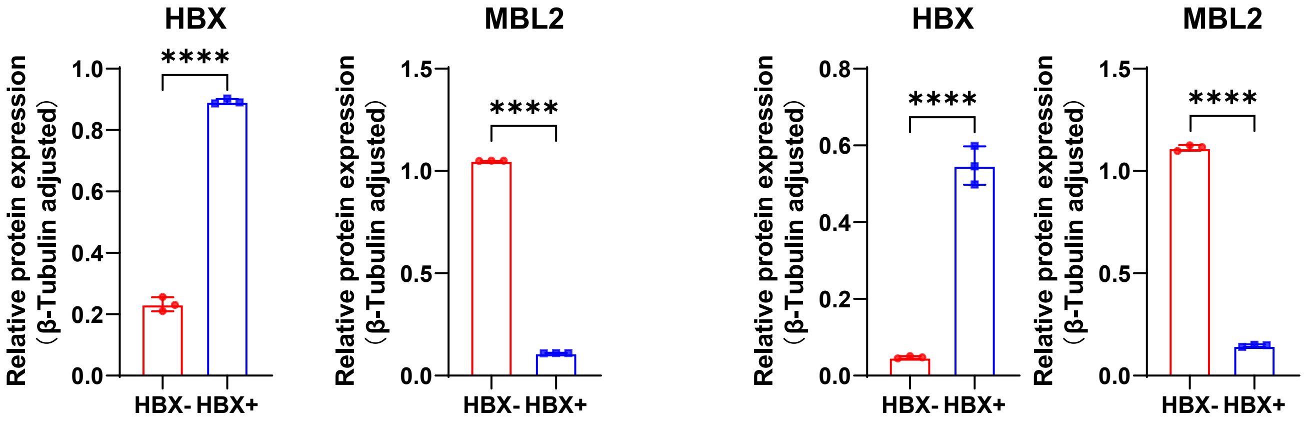

Figure 6-E

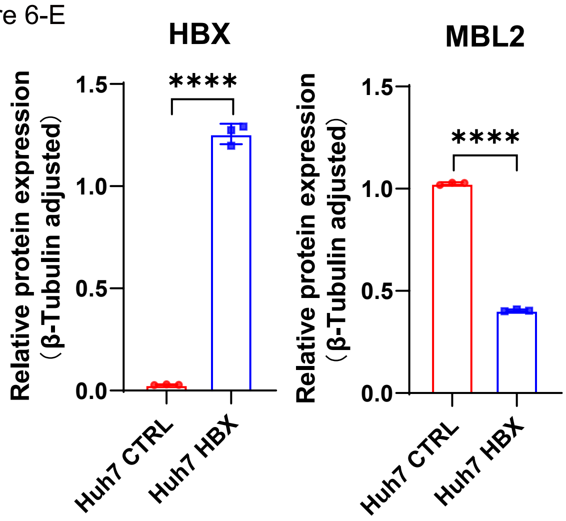

Supplement: S4 Data — (PDF) [file pbio.3003793.s010.pdf]
