# Supplementary material for: Mannose-binding lectin 2 secreted by hepatocellular carcinoma cells recruits and activates natural killer cells to reshape an immune-activated microenvironment
Source: PLoS Biol. 2026 May 20;24(5):e3003793. doi: 10.1371/journal.pbio.3003793 (PMC13189296; doi:10.1371/journal.pbio.3003793)

Figure 1-D

β-Tubulin

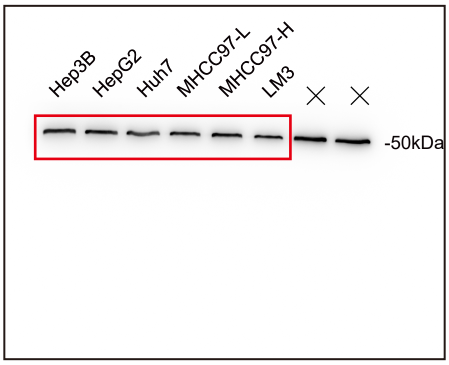

MBL2

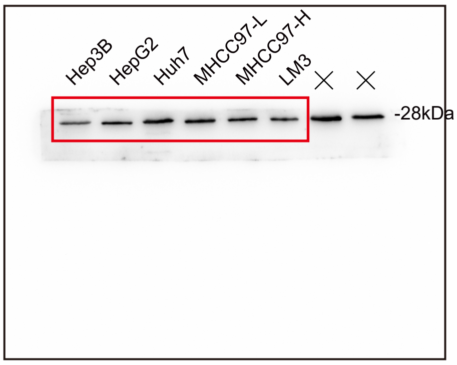

Figure 1-F

β-Tubulin

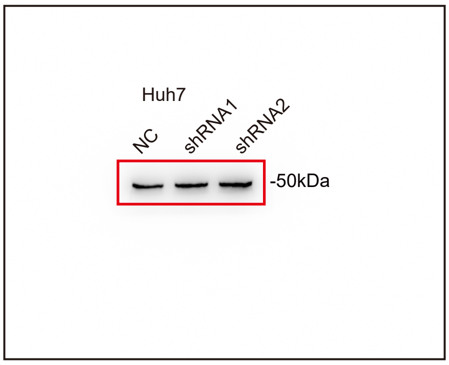

MBL2

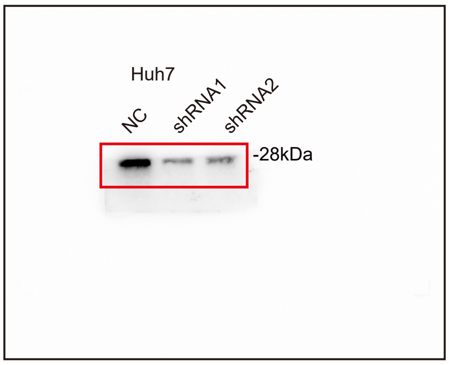

β-Tubulin

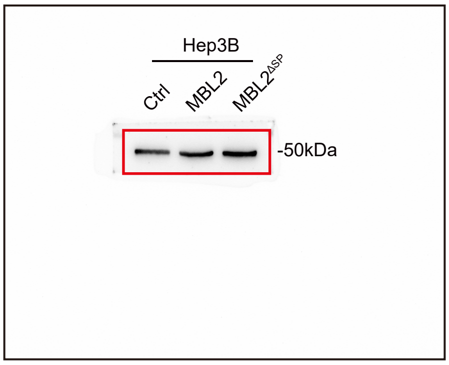

MBL2

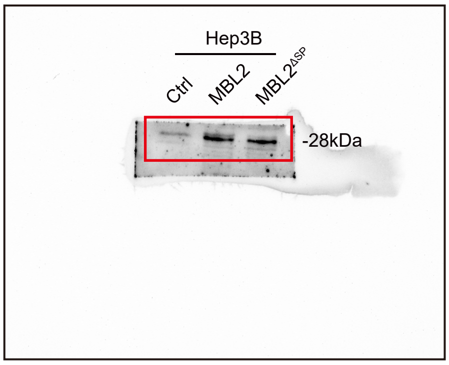

β-Tubulin

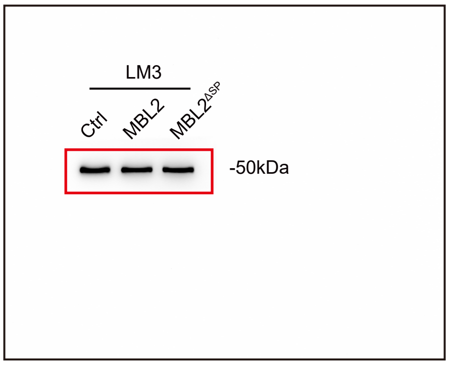

MBL2

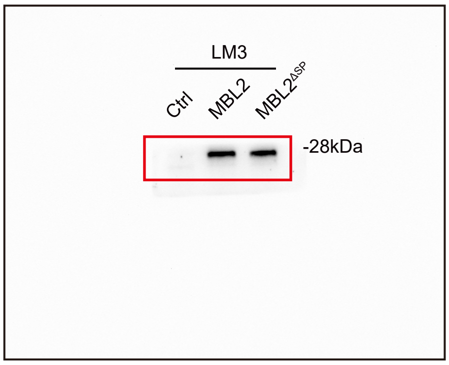

MBL2（CM）

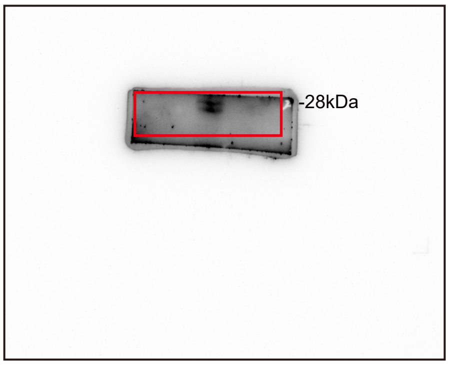

MBL2（CM）

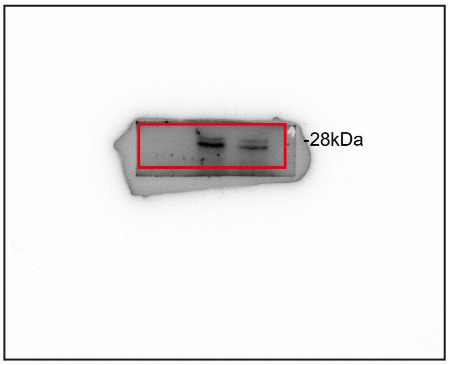

Figure 3-A

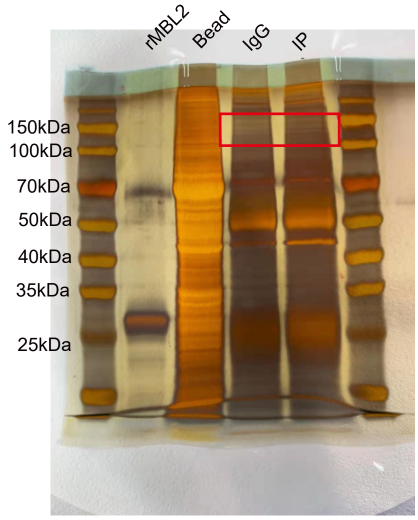

Figure 3-C

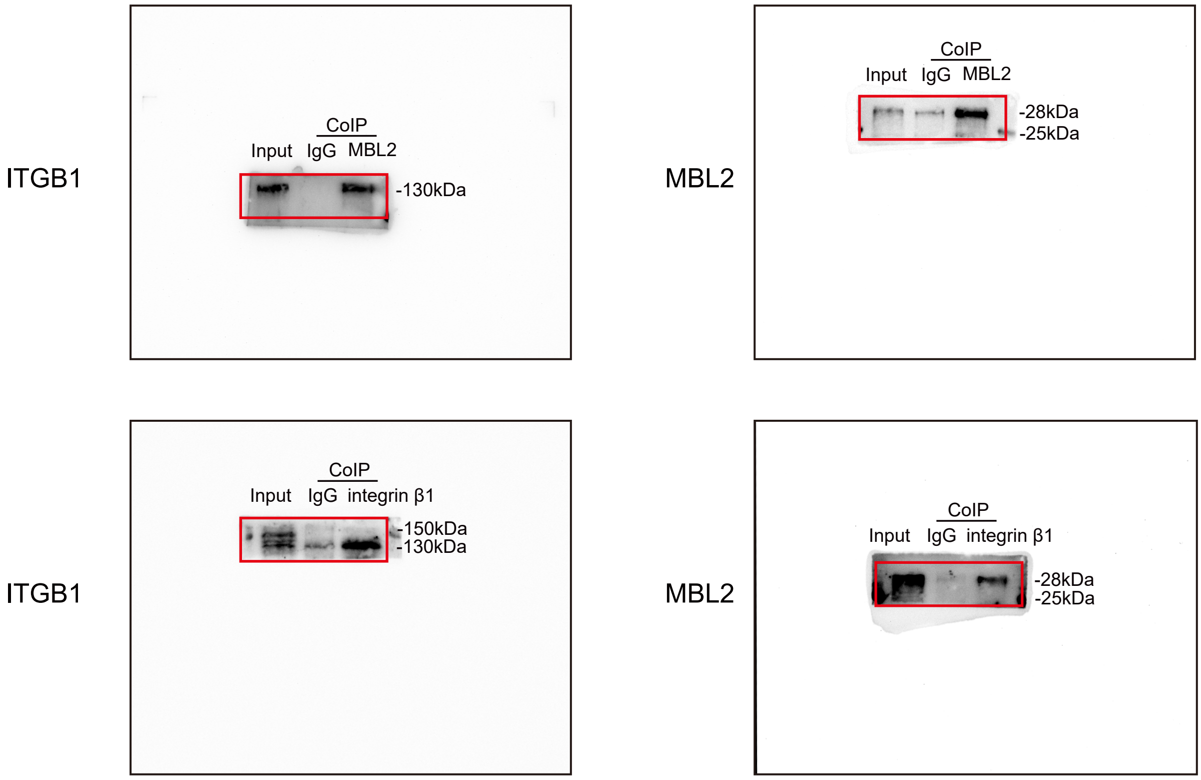

Figure 3-E

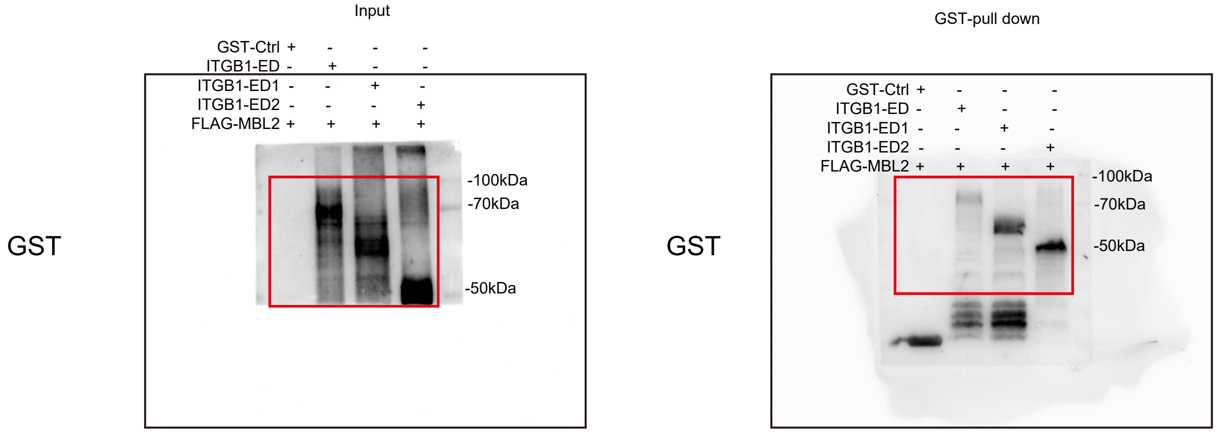

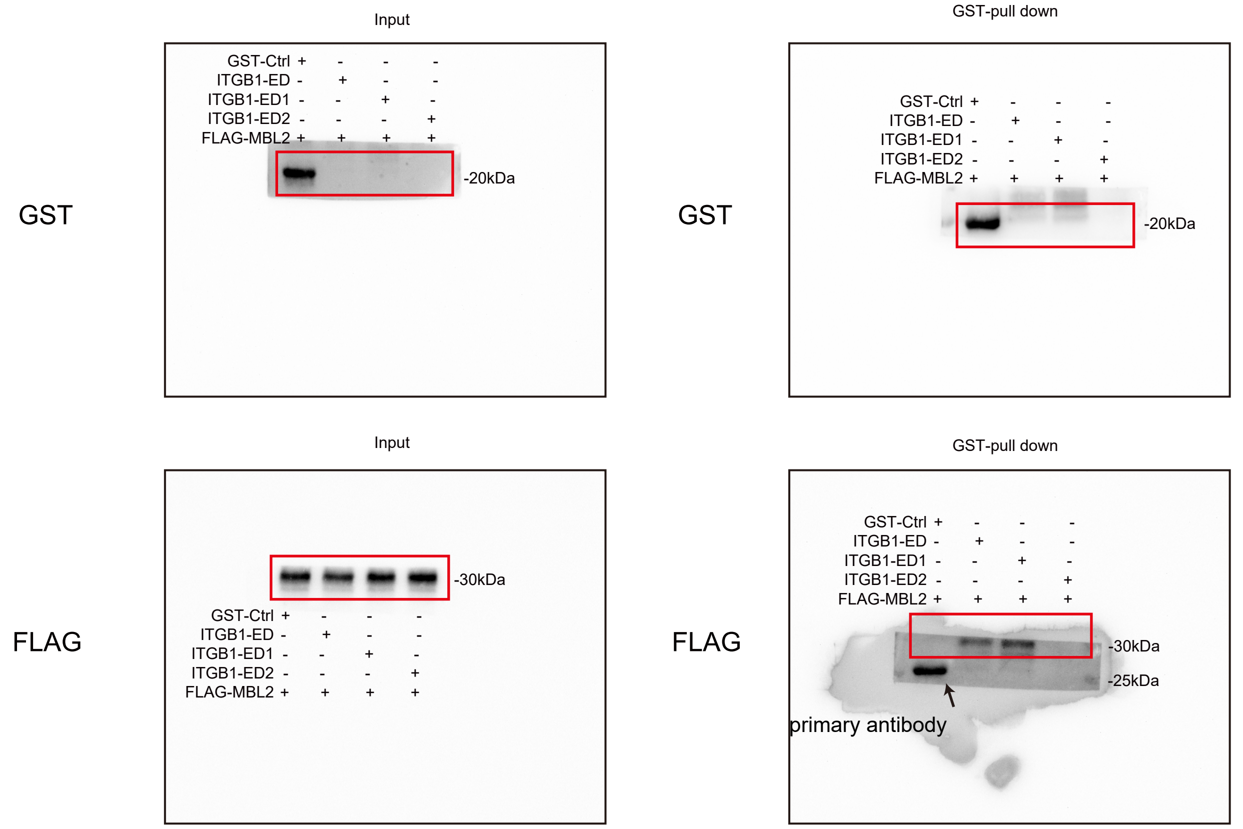
Input
GST-pull down
GST
GST-Ctrl
ITGB1-ED
ITGB1-ED1
ITGB1-ED2
FLAG-MBL2
-20kDa
FLAG
-30kDa
-25kDa
primary antibody

Figure 3-K

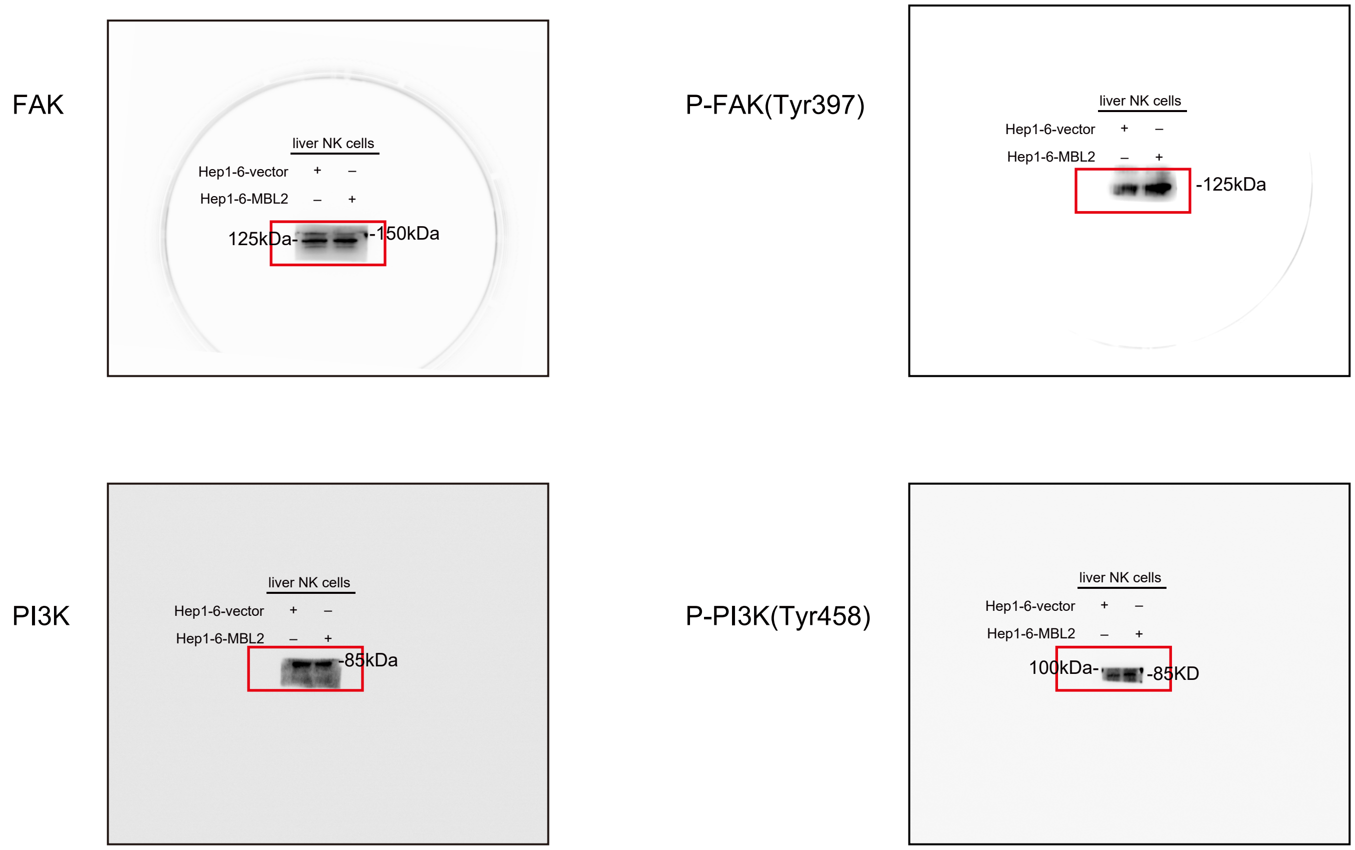
FAK
P-FAK(Tyr397)
PI3K
P-PI3K(Tyr458)
liver NK cells
Hep1-6-vector
Hep1-6-MBL2
125kDa-
-150kDa
-125kDa
-85kDa
100kDa-
-85KD

AKT

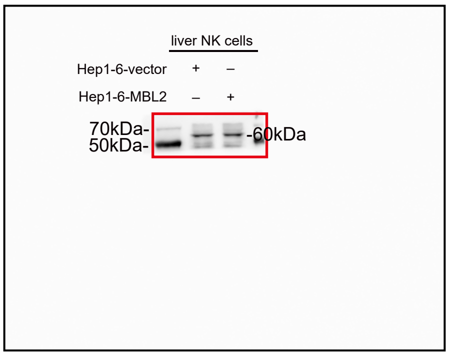

P-AKT(Ser473)

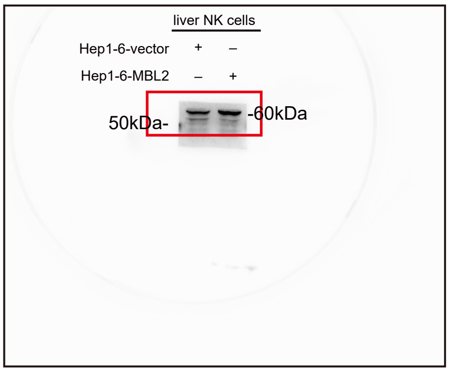

β-Actin

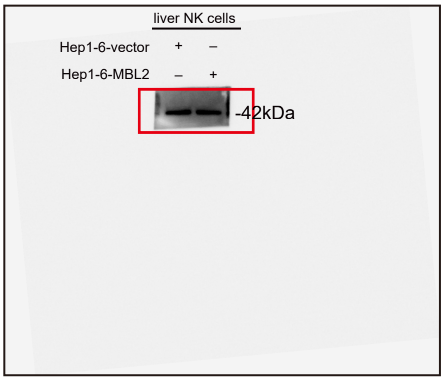

β-Actin

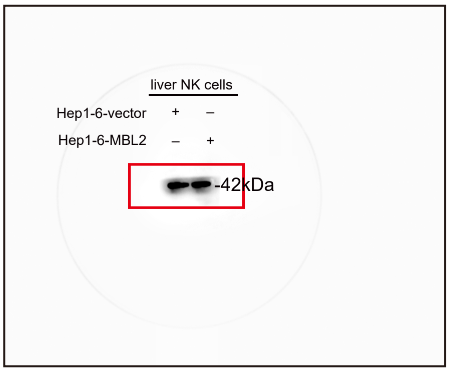

Figure 3-M

FAK

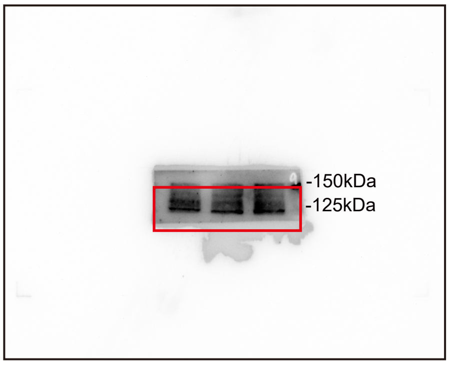

P-FAK(Tyr397)

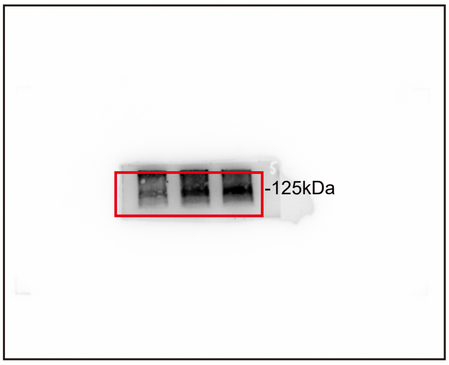

PI3K

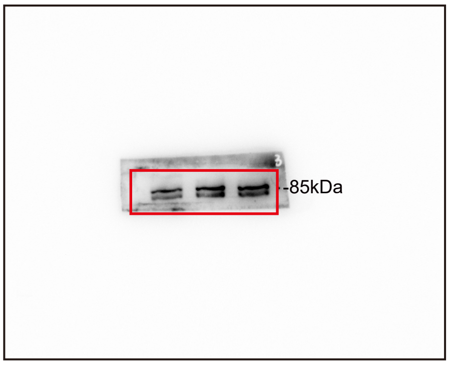

P-PI3K(Tyr458)

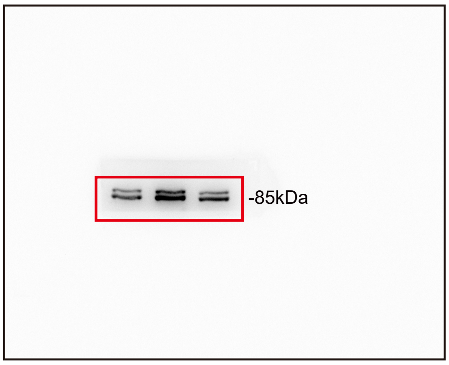

AKT

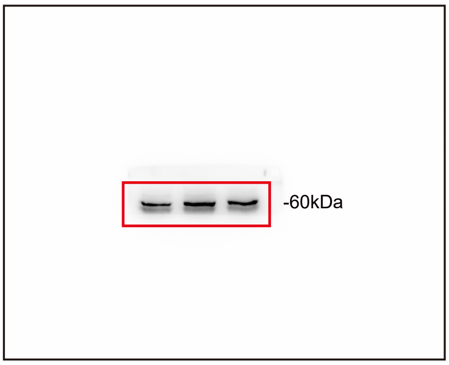

P-AKT(Ser473)

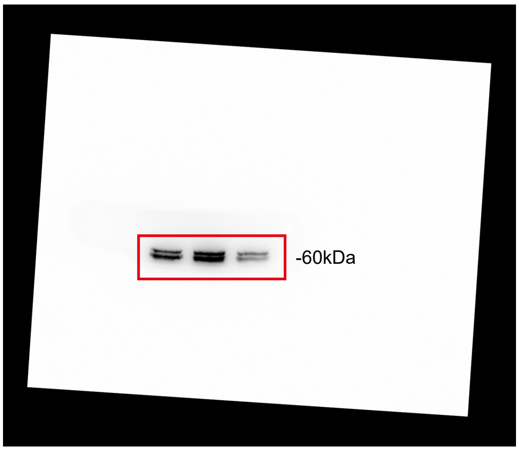

β-Actin

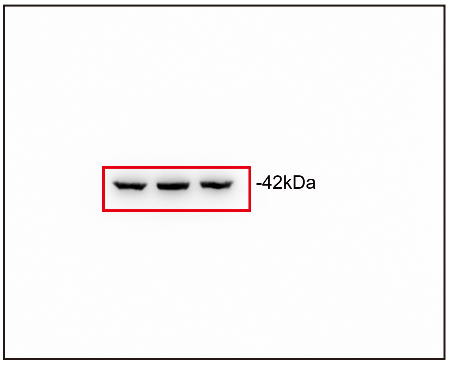

β-Actin

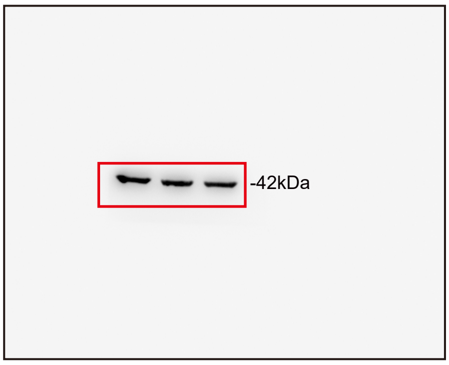

Figure 4-G

β-Tubulin

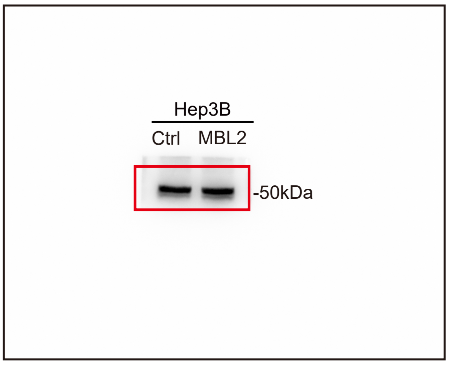

IFNGR1

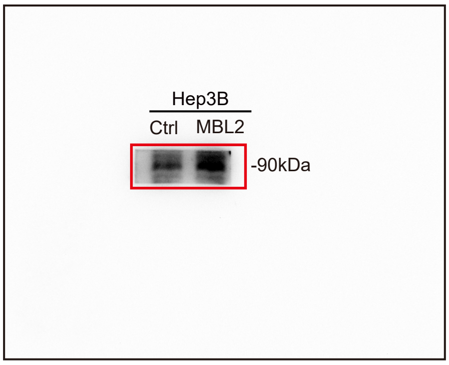

β-Tubulin

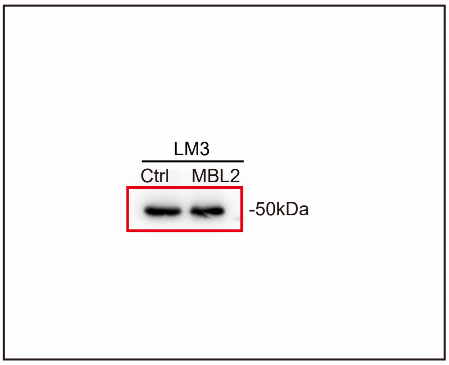

IFNGR1

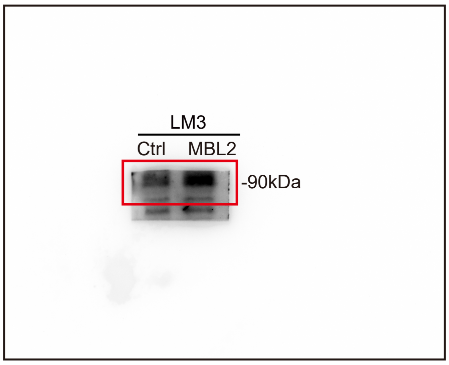

Figure 4-H

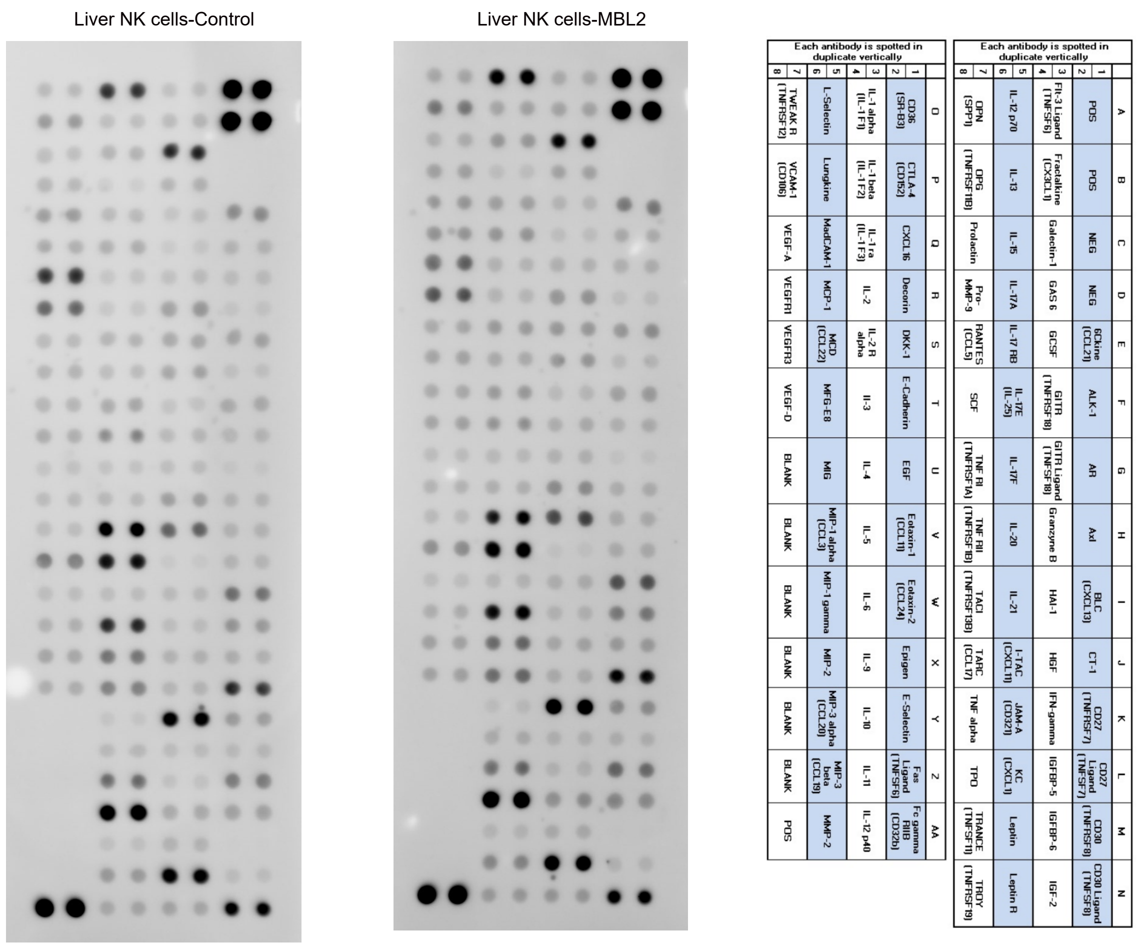

Figure 5-E

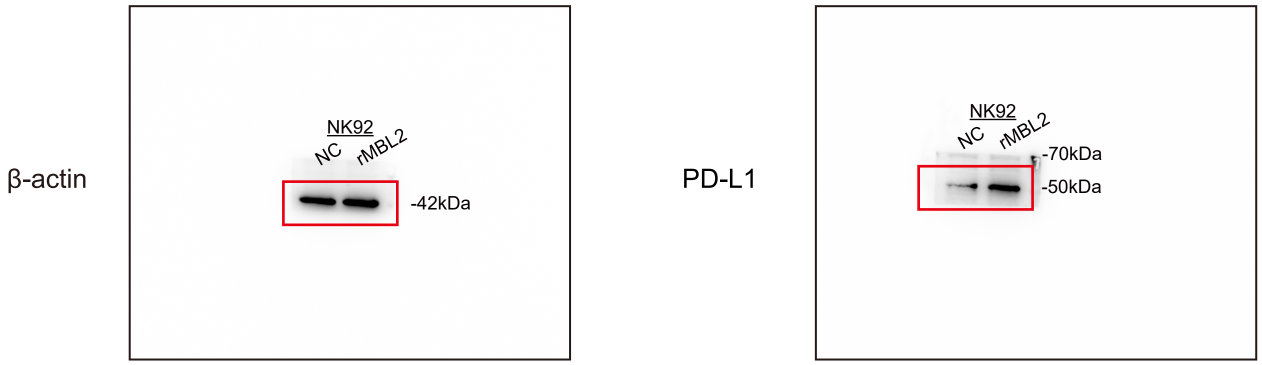

Figure 5-F

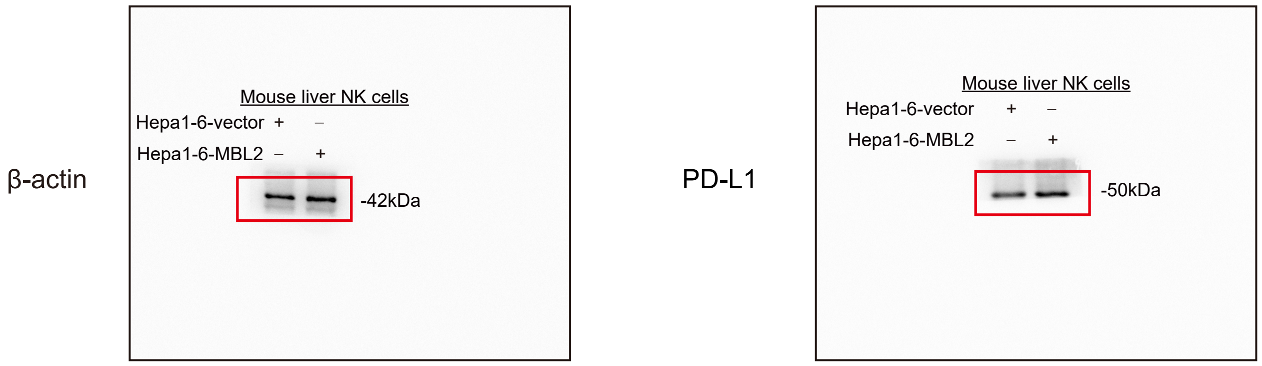

Figure 5-I

-28kDa

β-actin

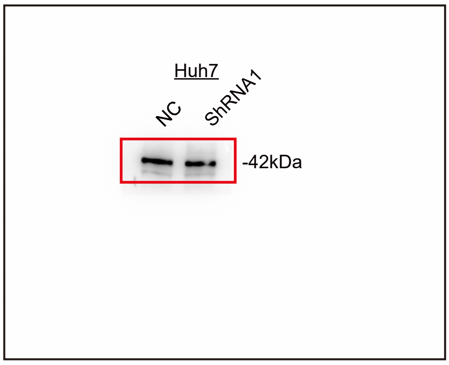

MBL2

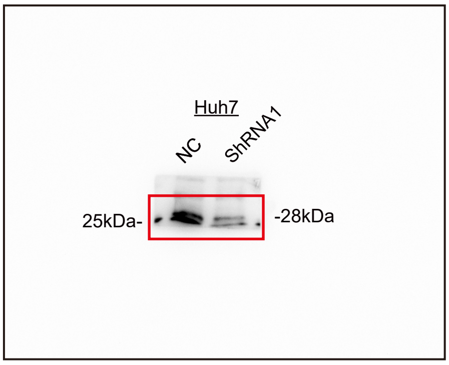

PD-L1

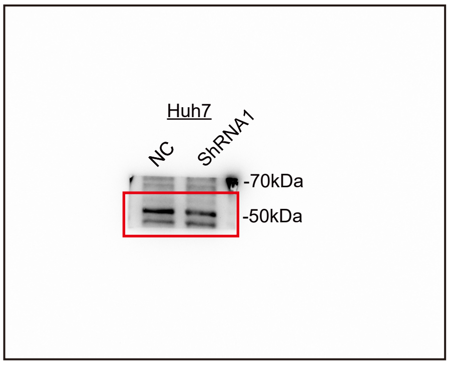

β-actin

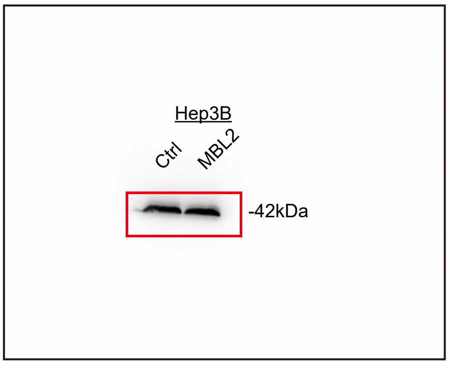

MBL2

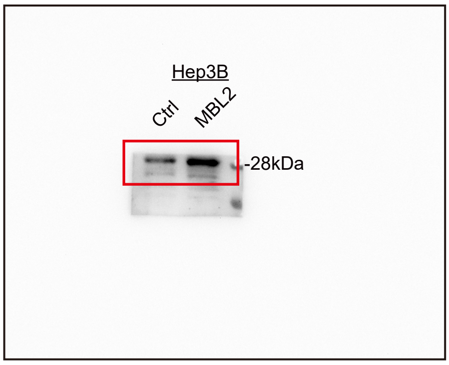

PD-L1

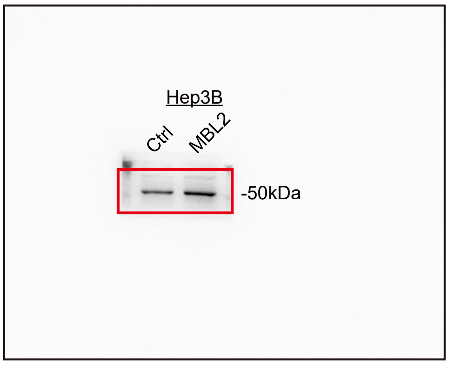

β-actin

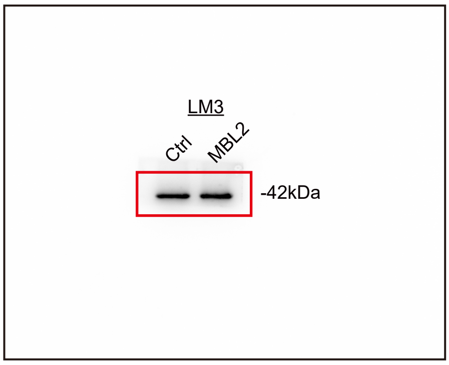

MBL2

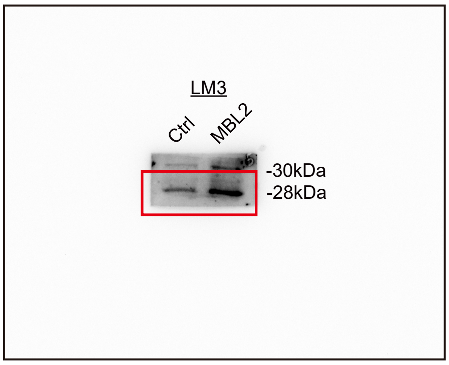

PD-L1

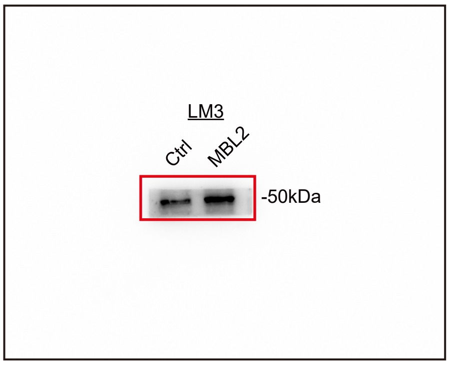

Figure 6-B

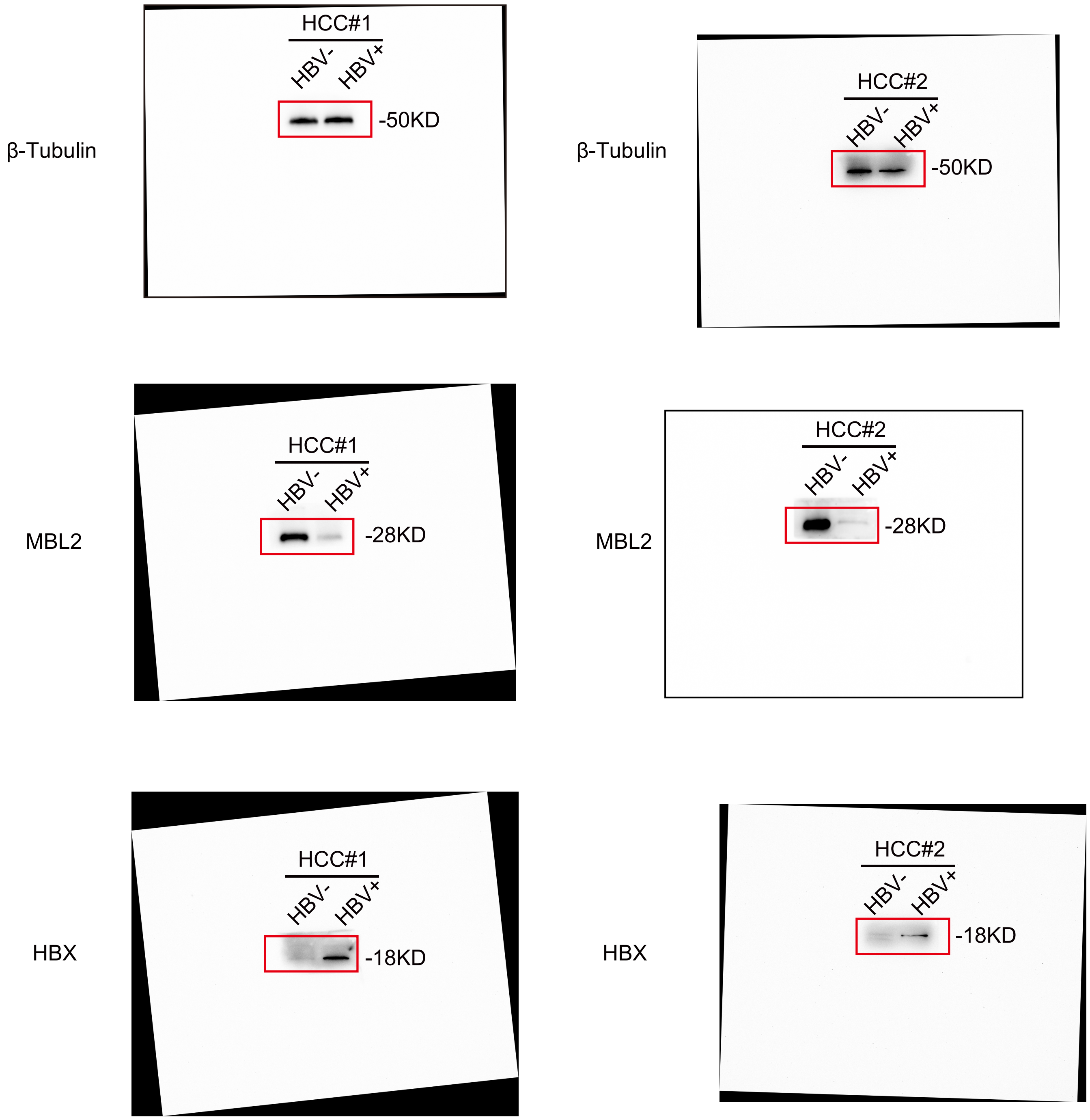
β-Tubulin
HCC#1
HBV-
HBV+
-50KD
β-Tubulin
HCC#2
HBV-
HBV+
-50KD
MBL2
HCC#1
HBV-
HBV+
-28KD
MBL2
HCC#2
HBV-
HBV+
-28KD
HBX
HCC#1
HBV-
HBV+
-18KD
HBX
HCC#2
HBV-
HBV+
-18KD

Figure 6-E

β-Tubulin

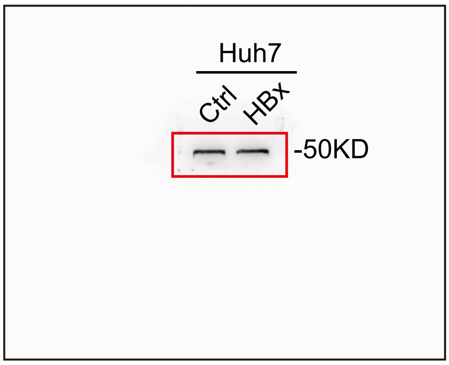

HBX

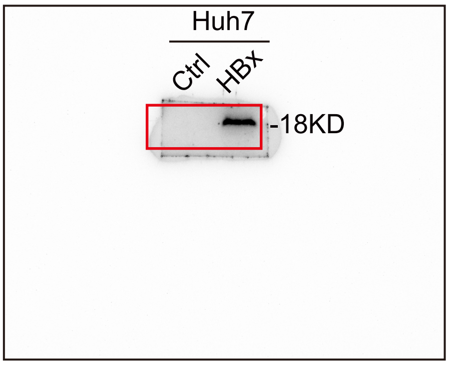

MBL2

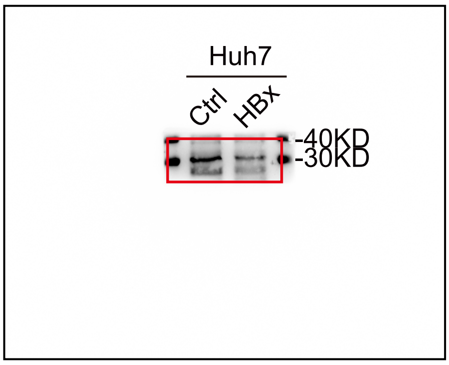

Figure 6-G

293T

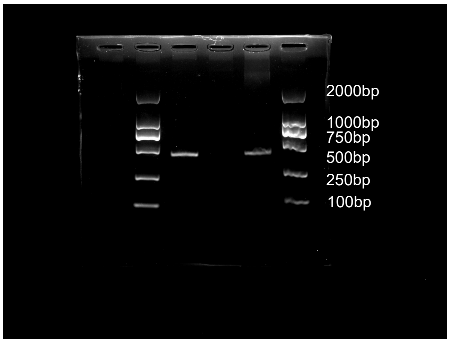

Figure 7-E

β-Tubulin

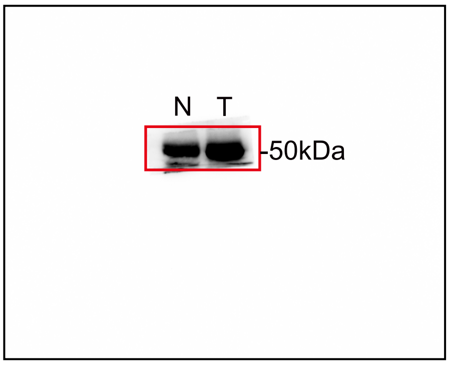

MBL2

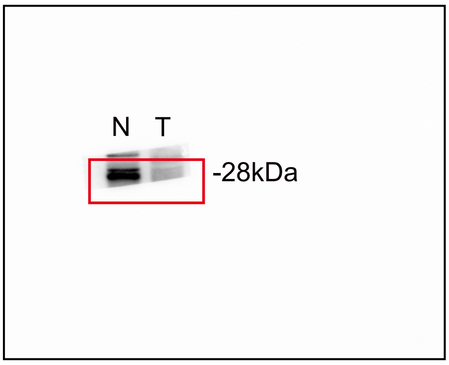

β-Tubulin

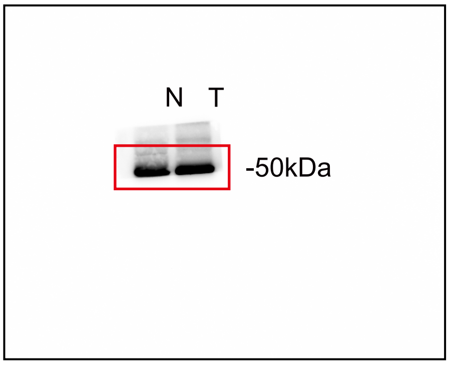

MBL2

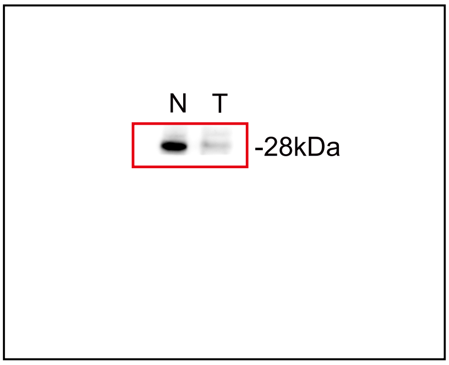

β-Tubulin

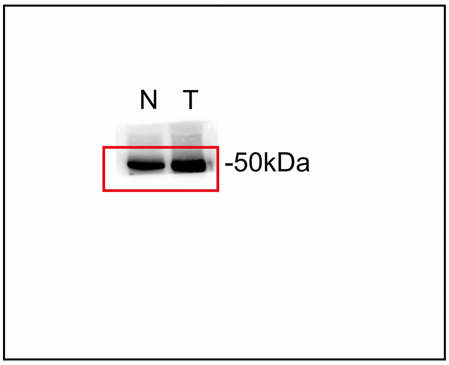

MBL2

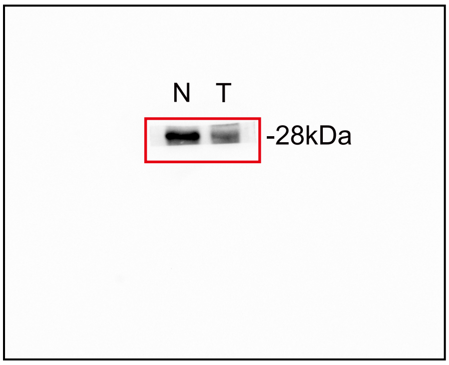

β-Tubulin

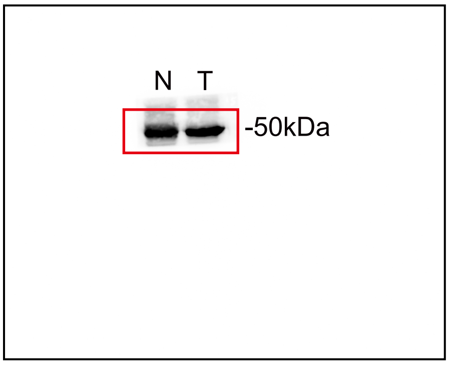

MBL2

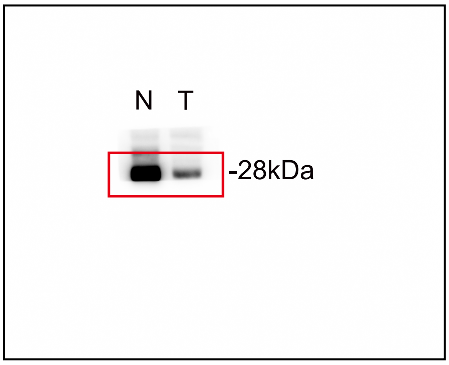

β-Tubulin

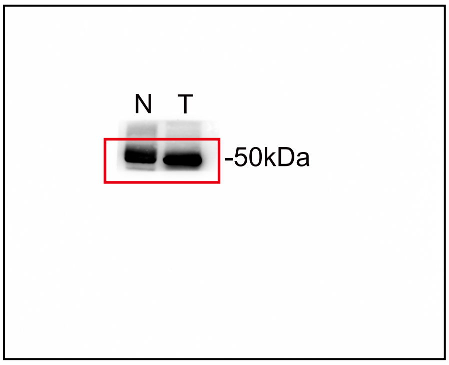

MBL2

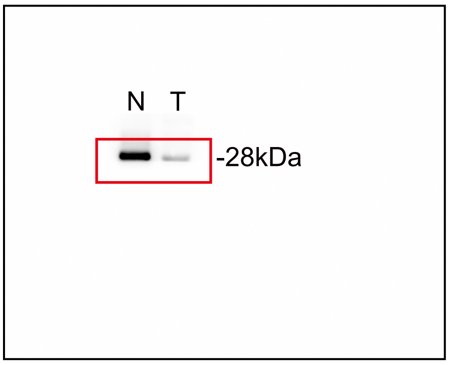

β-Tubulin

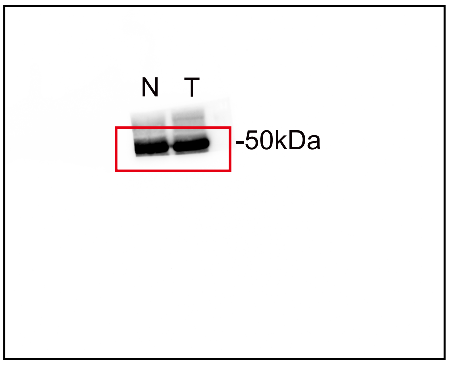

MBL2

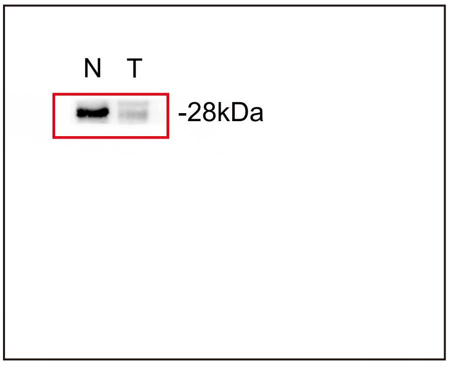

β-Tubulin

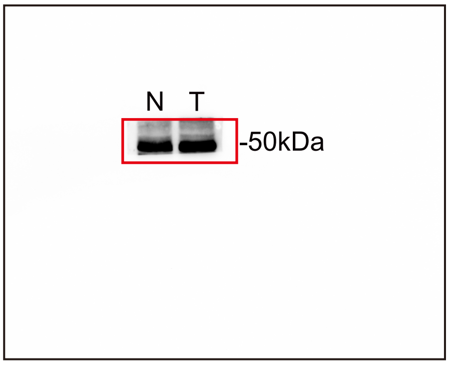

MBL2

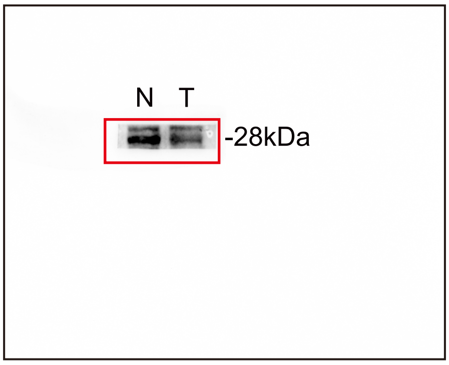

β-Tubulin

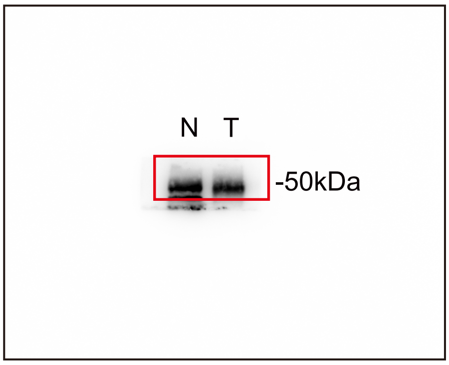

MBL2

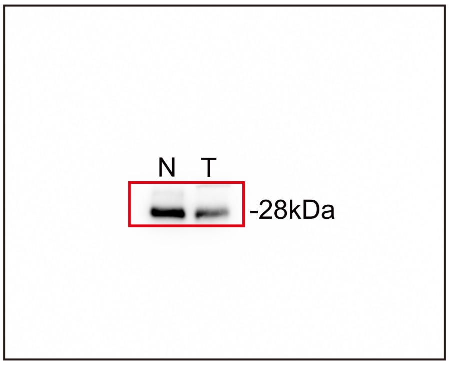

β-Tubulin

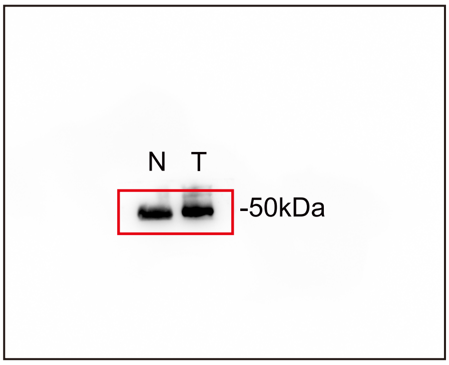

MBL2

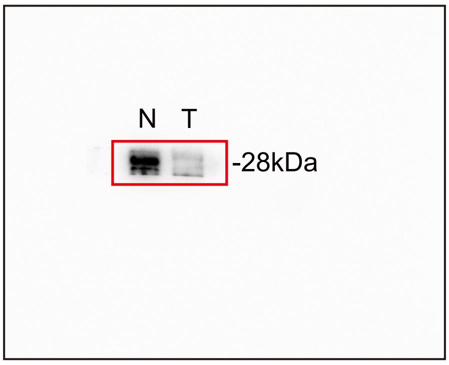

β-Tubulin

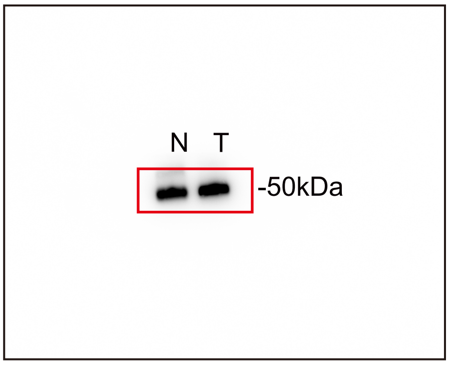

MBL2

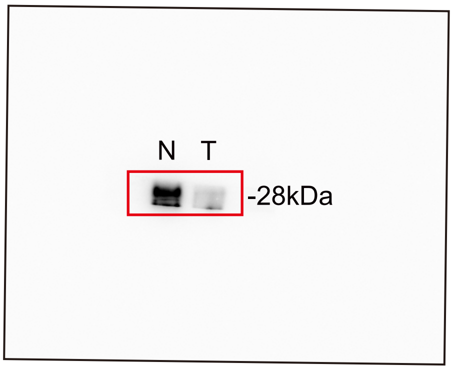

β-Tubulin

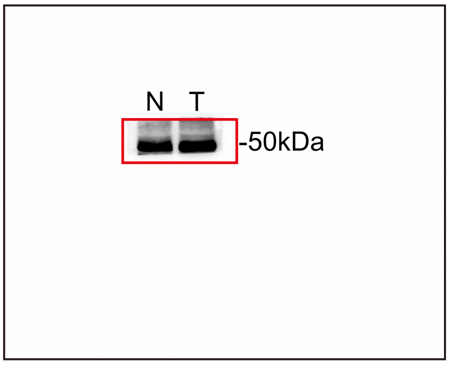

MBL2

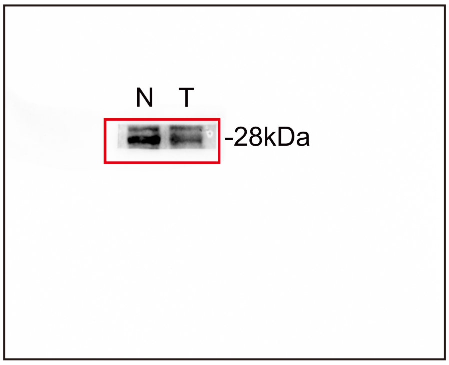

β-Tubulin

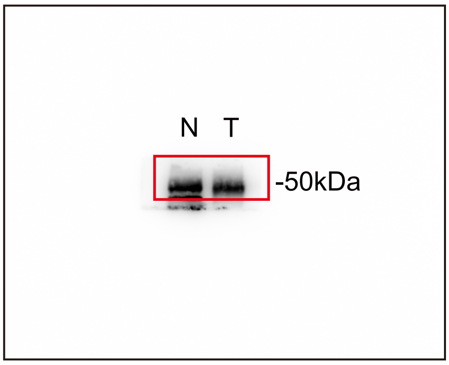

MBL2

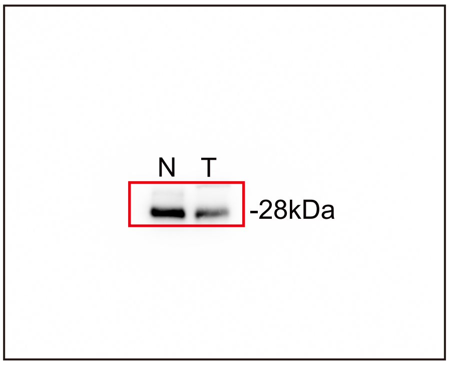

β-Tubulin

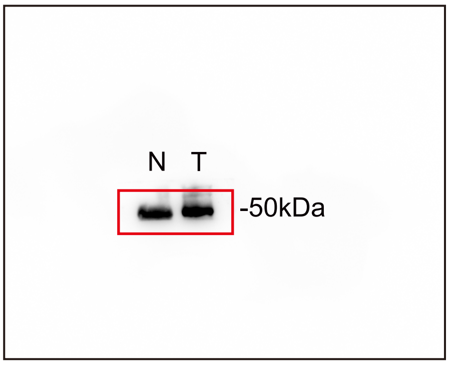

MBL2

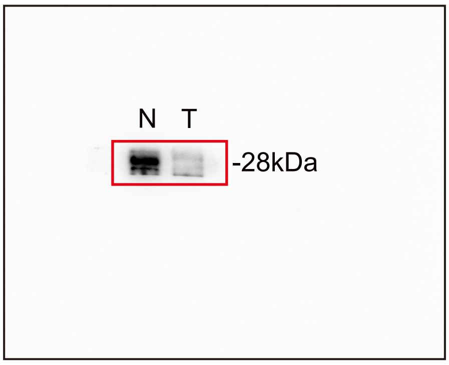

β-Tubulin

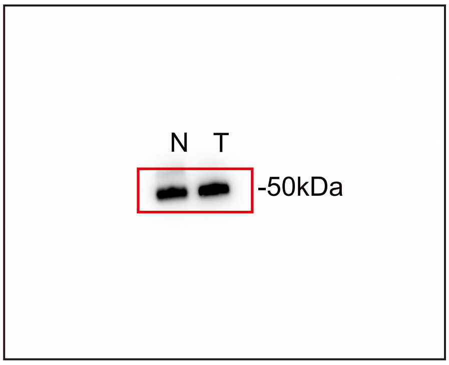

MBL2

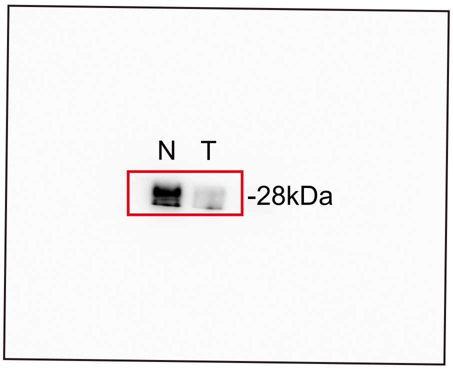

Supplement: S1 Raw Images — (PDF) [file pbio.3003793.s011.pdf]
